# Metal Complexes with Naphthalene-Based Acetic Acids as Ligands: Structure and Biological Activity

**DOI:** 10.3390/molecules28052171

**Published:** 2023-02-26

**Authors:** Marialena Lazou, Spyros Perontsis, George Psomas

**Affiliations:** Laboratory of Inorganic Chemistry, Department of Chemistry, Aristotle University of Thessaloniki, GR-54124 Thessaloniki, Greece

**Keywords:** naphthylacetic acids, naproxen, pyreneacetic acid, coordination compounds, structures, spectroscopic properties, biological activity

## Abstract

Naproxen (6–methoxy–α–methyl–2–naphthaleneacetic acid), 1–naphthylacetic acid, 2–naphthylacetic acid and 1–pyreneacetic acid are derivatives of acetic acid bearing a naphthalene-based ring. In the present review, the coordination compounds of naproxen, 1– or 2–naphthylacetato and 1–pyreneacetato ligands are discussed in regard to their structural features (nature and nuclearity of metal ions and coordination mode of ligands), their spectroscopic and physicochemical properties and their biological activities.

## 1. Introduction

Carboxylates are intriguing ligands since they may offer two carboxylato oxygen atoms to form up to four metal–oxygen bonds resulting in interesting structures. Depending on the nature of the carboxylato ligands and in combination with the choice of metal ions, the resultant metal complexes may present interesting properties from many points of view, such as magnetic, photochemical, and biological properties [1,2,3,4,5,6,7,8]. Among the carboxylato ligands, the acetato ligands and their derivatives are the most studied ones, leading to a variety of metal complexes, nuclearities and properties [7,8,9].

**Figure 1:**
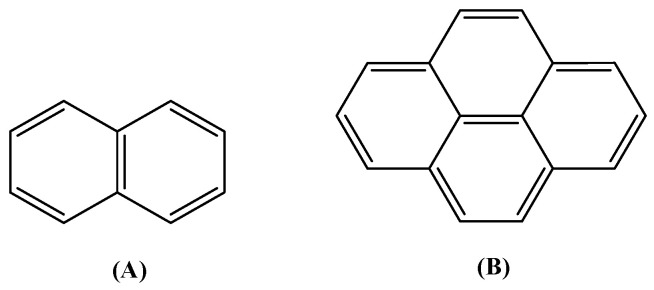
(**A**) The naphthalene ring. (**B**) The pyrene ring.

**Figure 2:**
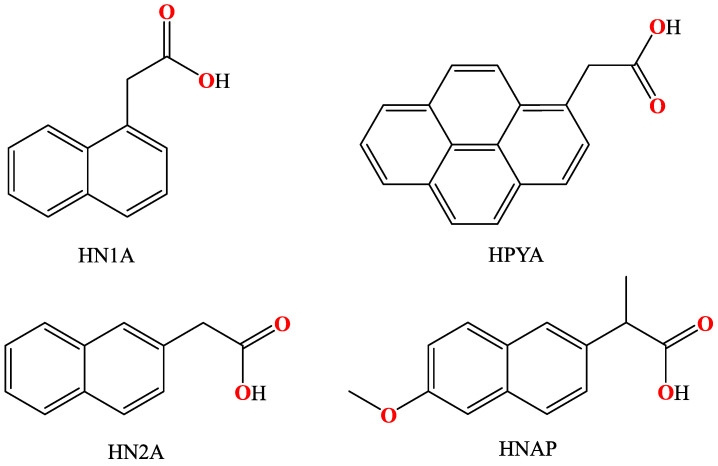
The syntax formula of 1–naphthylacetic acid (=HN1A), 2–naphthylacetic acid (=HN2A), naproxen (=HNAP) and 1–pyreneacetic acid (=HPYA).

As a continuation of our previous perspective and review articles [10,11] concerning the structures and biological properties of metal complexes with carboxylato antimicrobial and anti-inflammatory drugs as ligands, a search of the Cambridge Crystallographic Data Centre (CCDC) database regarding the structures of metal complexes with acetato ligands was performed. Our attention was drawn by the presence of the fused polycyclic aromatic hydrocarbon ring on the acetato derivatives which gave a series of interesting metal complexes. Therefore, a thorough search of the CCDC database [12] was recorded and revealed a series of metal complexes with acetato ligands which are attached to a naphthalene or a pyrene ring (Figure 1). From the point of view of the acetato ligands, only four compounds used as ligands were found, i.e., 1–naphthylacetic acid, 2–naphthylacetic acid, naproxen and 1–pyreneacetic acid (Figure 2).

In the present review, the structural features (nature and number of metal ions, coordination mode of ligands and nature and coordination of co-ligands) and the physicochemical and spectroscopic characterization as well as the properties and potential (mainly biological) applications of the reported metal complexes of the 1–naphthylacetato, 2–naphthylacetato, 1–pyreneacetato and naproxen ligands will be presented and discussed.

## 2. Information on the Acids, Co-Ligands and Metal Ions

### 2.1. General Considerations for the Acids

Naphthylacetic acids (naphthoic acids or naphthaleneacetic acids, HNA) are compounds containing a planar naphthalene ring bound to an acetic group. Based on the position of the aromatic ring where the acetic group is located, they are characterized either as 1–naphthylacetic acid (α–naphthylacetic acid, HN1A) or 2–naphthylacetic acid (β–naphthylacetic acid, HN2A) (Figure 2). Both HNAs are white amorphous solids and are mainly used as plant growth regulators [13]. They are synthetic plant auxin hormones and are commercially used in agriculture and house-gardening as rooting agents [14]. As typical compounds of the auxin family, their use and concentration should be controlled since they may become toxic to plants at higher concentrations and to animals at lower concentrations [15]. 1–naphthylacetic acid has a more extended use than 2–naphthylacetic acid. An important advantage of 1–naphthylacetic is its stability, since, before its entrance in the tissue, it is not destructed by light or by oxidation [16]. The main actions of 1–naphthylacetic acid comprise the activation of cell division, cell elongation, photosynthesis, RNA synthesis, membrane permeability and water uptake in plants [13]. Therefore, 1–naphthylacetic acid is often used to increase the production by lowering and delaying the preharvest drop of crops and hanging fruits and improving their quality (e.g., apples, olives, oranges and hazelnuts [17,18]). Another function of both HNAs is their involvement in the formation of flower buds of tobacco [16] and pistachio plants [19]. In addition, 2–naphthylacetic acid is the main initial metabolite of anaerobic degradation of naphthalene and 2–methylnaphthalene from sulfate-producing bacteria [20].

Naproxen (6–methoxy–α–methyl–2–naphthaleneacetic acid, HNAP, Figure 2) is an established non-steroidal anti-inflammatory drug (NSAID) [21]. It is a common anti-inflammatory, analgesic and antipyretic medicament and is administered for the treatment of painful dysmenorrheal [22], chronic migraine, kidney stones, rheumatoid arthritis and osteoarthritis [23]. The use of naproxen induces milder side effects regarding increased blood pressure compared to ibuprofen [24] or celecoxib [25] and stomach ulcers compared to indomethacin [26] and other NSAIDs [27]. Naproxen may produce analgesic and anti-inflammatory effects by blocking non-selectively both cyclooxygenase (COX) enzymes (COX–1 and COX–2) [28,29] and subsequently decreasing the synthesis of prostaglandins [21]. The absorption of naproxen is rapid and complete upon oral or rectal administration [21,30]. Recently, a carbon nanotube functionalized by cationic hyperbranched polyethyleneimine was examined as a potential effective carrier of naproxen [31].

1–Pyreneacetic acid (HPYA, Figure 2) is a pyrene derivative. The pyrene ring is the smallest peri-fused polycyclic aromatic hydrocarbon [32] and, because of its 16 π–electrons [33], it may provide interesting electronic properties [34] exploitable for various applications such as the preparation of chemosensors [35]. In particular, the reaction of 1–pyreneacetic acid with *N*–hydroxysuccinimide resulted in the formation of a Pd(II)–thioether amide chemosensor having high selectivity over Pd(0) [36]. The photoluminescence of 1–pyreneacetic acid (emission band with λ_max_ ~400 nm, when excited at 345 nm) was also taken into consideration for the fabrication of dual-functionalized polymer nanotubes which could act as substrates for molecular probes and DNA carriers [37]. Recently, 1–pyreneacetic acid was used for the functionalization of a graphene/self-assembled monolayer modified gold electrode which could be used for the study of electron transfer of cytochrome *c* by electrochemical techniques [38]. 1–Pyreneacetic acid has also been proposed as a titrating reagent for the titration of organolithium and Grignard reagents [39] since the end point of such titrations is significantly accurate [40].

### 2.2. The Co-Ligands

Besides the four acids, i.e., 1–naphthylacetic acid, 2–naphthylacetic acid, naproxen and 1–pyreneacetic acid, used as the main ligands in the complexes under study, another thing that is significant is the role of co-ligands in these complexes, since the number of binary complexes is very limited and most of them are mixed ligand complexes. Most of the co-ligands found in the reported complexes were either O–donor ligands or N–donor ligands, and, in few cases, P–, B– or C–donors were also found.

**Figure 3:**
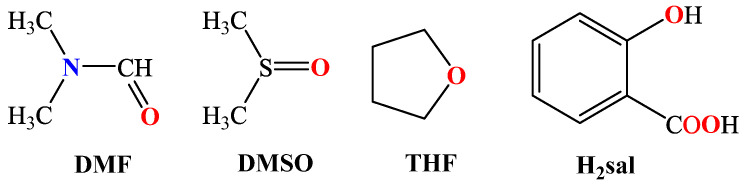
The syntax formula of oxygen donors: DMF = *N,N*–dimethylformamide; DMSO = dimethylsulfoxide; THF = tetrahydrofuran and H_2_sal = salicylic acid.

**Figure 4:**
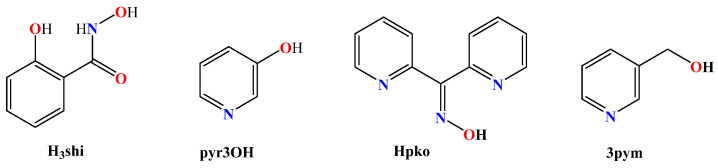
The syntax formula of the N^O–donors: H_3_shi = salicylhydroxamic acid; pyr3OH = pyridin–3–ol; Hpko = di(2–pyridyl)ketone oxime and 3pym = 3–pyridylmethanol.

Among the O–donor ligands, the most frequently found are those originating from a solvent used, i.e., H_2_O, methanol (CH_3_OH), *N,N*–dimethylformamide (DMF), dimethylsulfoxide (DMSO) or tetrahydrofuran (THF) (Figure 3). In certain cases, oxygen atoms were also provided in combination with nitrogen atoms by N^O–donor co-ligands such as salicylhydroxamic acid (H_3_shi), pyridin–3–ol (pyr3OH), di(2–pyridyl)ketone oxime (Hpko) and 3–pyridylmethanol (3pym) (Figure 4).

**Figure 5:**
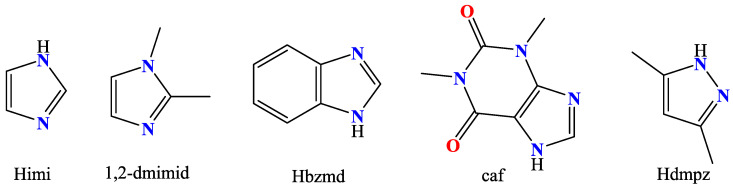
The syntax formula of imidazole derivatives: Himi = imidazole; 1,2–dmimid = 1,2–dimethylimidazole; Hbzmd = 1H–benzimidazole; caf = caffeine and Hdmpz = 3,5–dimethylpyrazole.

**Figure 6:**
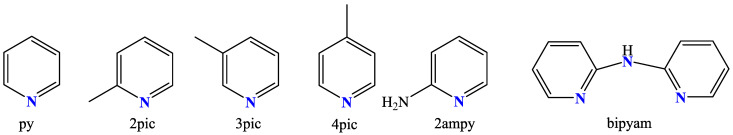
The syntax formula of pyridine derivatives: py = pyridine; 2pic = 2–picoline; 3pic = 3–picoline; 4pic = 4–picoline; 2ampy = 2–aminopyridine and bipyam = 2,2′–bipyridylamine.

**Figure 7:**
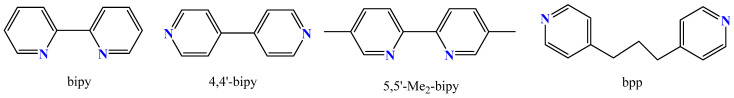
The syntax formula of bipyridine derivatives: bipy = 2,2′–bipyridine; 4,4′–bipy = 4,4′–bipyridine; 5,5′–Me_2_–bipy = 5,5′–dimethyl–2,2′–bipyridine and bpp = 1,3–bis(4–pyridyl)propane.

**Figure 8:**
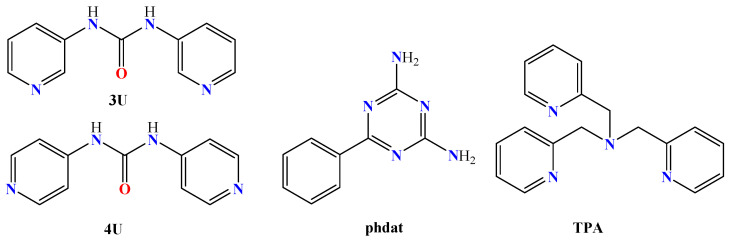
The syntax formula of the co-ligands: 3U = 1,3–dipyridin–3–ylurea; 4U = 1,3–dipyridin–4–ylurea; phdat = 2,4–diamine–6–phenyl–1,3,5–triazine and TPA = tris(2–pyridyl)amine.

**Figure 9:**
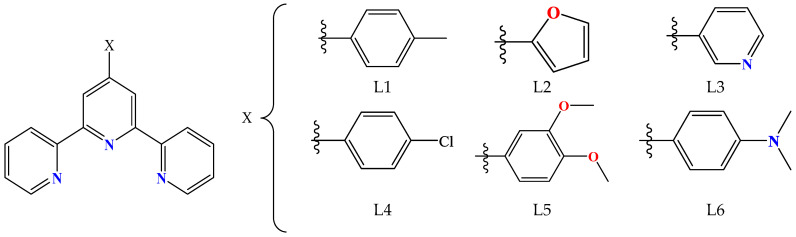
The syntax formula of 2,2′:6′,2″–terpyridine derivatives: L1 = 4′–(4–tolyl)–2,2′:6′,2″–terpyridine; L2 = 4′–(furan–2–yl)–2,2′:6′,2″–terpyridine; L3 = 4′–(pyridin–3–yl)–2,2′:6′,2″–terpyridine; L4 = 4′–(4–chlorophenyl)–2,2′:6′,2″–terpyridine; L5 = 4′–(3,4–dimethoxyphenyl)–2,2′:6′,2″–terpyridine and L6 = 4′–(4–dimethylaminophenyl)–2,2′:6′,2″–terpyridine.

**Figure 10:**
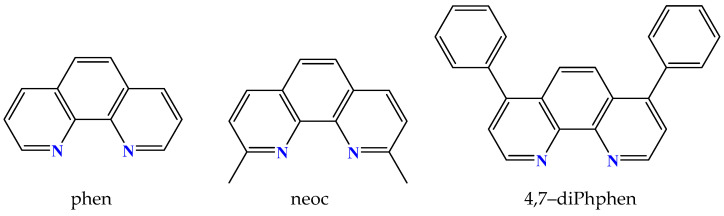
The syntax formula of phenanthroline derivatives: phen = 1,10–phenanthroline; neoc = neocuproine = 2,9–dimethyl–1,10–phenanthroline and 4,7–diPhphen = 4,7–diphenyl–1,10–phenanthroline.

**Figure 11:**
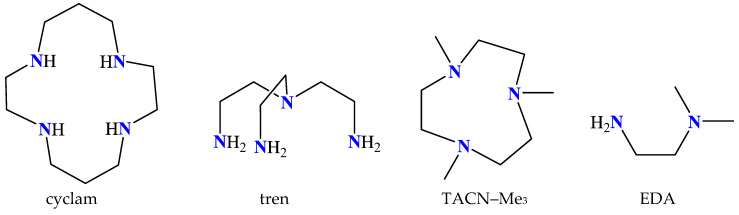
The syntax formula of the nitrogen–donors: cyclam = 1,4,8,11–tetraazacyclotetradecane; TACN–Me_3_ = 1,4,7–trimethyl–1,4,7–triazacyclononane; tren = tris(2–aminoethyl)amine and EDA = N,N–dimethylethane–1,2–diamine.

The nitrogen–donor co-ligands were found in most of the reported metal complexes under study. Their choice was mainly based on their structures, since in many these co-ligands contained single or fused aromatic rings which may have led to better stabilization of the structure due to the formation of π–π interactions with the naphthalene or pyrene rings of the acetato ligands, and on their potential synergism regarding other properties, such as photochemical and biological applications. The nitrogen–donor co-ligands may be categorized into the following:

(i) Imidazole derivatives, including imidazole (Himi) and compounds containing a 5-membered heterocyclic aromatic ring, such as 1,2–dimethylimidazole (1,2–dmimid), 1H–benzimidazole (Hbzmd), caffeine (caf) and 3,5–dimethylpyrazole (Hdmpz) (Figure 5);
(ii) Pyridine derivatives, including pyridine (py) and compounds containing a 6-membered heterocyclic aromatic ring, such as 2–picoline (2pic), 3–picoline (3pic), 4–picoline (4pic), 2–aminopyridine (2ampy) and 2,2′–bipyridylamine (bipyam) (Figure 6), or 2,2′–bipyridine derivatives including 2,2′–bipyridine (bipy), 4,4′–bipyridine (4,4′–bipy), 5,5′–dimethyl–2,2′–bipyridine (5,5′–Me_2_–bipy) and 1,3–bis(4–pyridyl)propane (bpp) (Figure 7), as well as 1,3–dipyridin–3–ylurea (3U), 1,3–dipyridin–4–ylurea (4U), 2,4–diamine–6–phenyl–1,3,5–triazine (phdat) and tris(2–pyridyl)amine (TPA) (Figure 8);
(iii) 2,2′:6′,2″–terpyridine derivatives, including 4′–(4–tolyl)–2,2′:6′,2″–terpyridine (L1), 4′–(furan–2–yl)–2,2′:6′,2″–terpyridine (L2), 4′–(pyridin–3–yl)–2,2′:6′, 2″–terpyridine (L3), 4′–(4–chlorophenyl)–2,2′:6′,2″–terpyridine (L4), 4′–(3,4–dimethoxyphenyl)–2,2′:6′,2″–terpyridine (L5) and 4′–(4–dimethylaminophenyl)–2,2′:6′,2″–terpyridine (L6) (Figure 9);
(iv) Phenanthroline derivatives, including 1,10–phenanthroline (phen) and its derivatives 2,9–dimethyl–1,10–phenanthroline (neocuproine, neoc) and 4,7–diphenyl–1,10–phenanthroline (4,7–diPhphen) (Figure 10);
(v) Alicyclic or aliphatic nitrogen–donors, such as 1,4,8,11–tetraazacyclotetradecane (cyclam), 1,4,7–trimethyl–1,4,7–triazacyclononane (TACN–Me_3_), tris(2–aminoethyl)amine (tren) and *N,N*–dimethylethane–1,2–diamine (EDA) (Figure 11).

**Figure 12:**
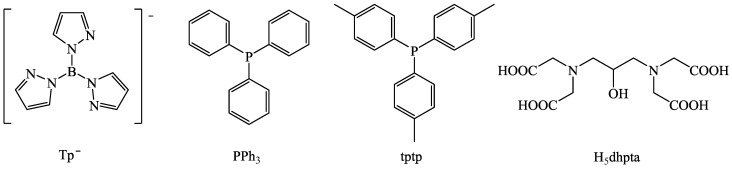
The syntax formula of the co–ligands: Tp^−^ = hydrotrispyrazolylborate; PPh_3_ = triphenylphosphine; tptp = tri(p–tolyl)phosphine and H_5_dhpta = 1,3–diamino–2–hydroxypropane–*N,N,N*′*,N*′–tetraacetic acid.

The use of the phosphines triphenylphosphine (PPh_3_) and tri(p–tolyl)phosphine (tptp) was also noted in some cases, while the hydrotrispyrazolylborate (Tp^−^) ligand and the polydentante 1,3–diamino–2–hydroxypropane–*N,N,N*′*,N*′–tetraacetic acid (H_5_dhpta) were noticed each in one structure (Figure 12). Diverse alkyl and aryl ions such as *n*–butyl (*n*–Bu), methyl (Me) and phenyl (Ph), as well as CO, were used as carbon atom donors.

### 2.3. Metal Ions of the Reported Complexes

**Figure 13:**
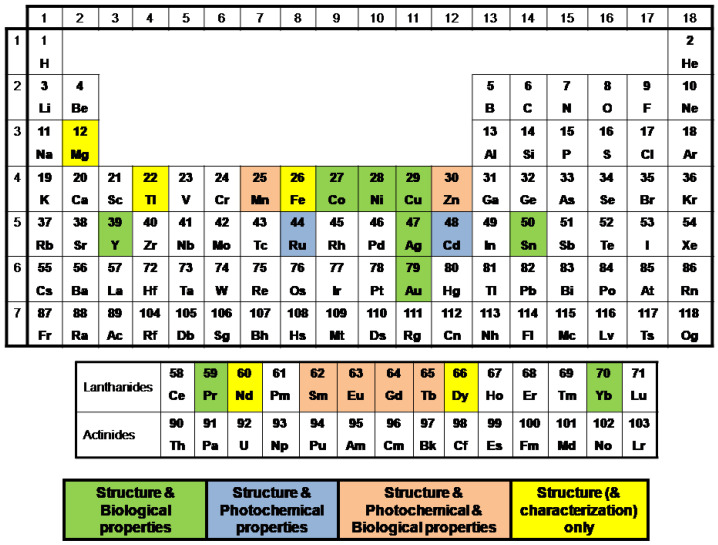
Periodic table of elements showing the metals participating in the coordination compounds with 1–naphthylacetato, 2–naphthylacetato, 1–pyreneacetato and naproxen ligands. (Color code: green = metal ions in characterized complexes studied for their biological properties; blue = metal ions in characterized complexes studied for their photochemical properties; orange = metal ions in characterized complexes studied for their photochemical and biological properties and yellow = metal ions in characterized complexes without any further studies).

Among the existing metal ions, a significant number of metal ions have been used to prepare coordination compounds with 1–naphthylacetato, 2–naphthylacetato, 1–pyreneacetato and naproxen ligands (Figure 13). The common first row transition metal ions were used in the majority of the reported metal complexes, i.e., Ti(IV), Mn(II/III), Fe(III), Co(II), Ni(II), Cu(II) and Zn(II). Coordination compounds with the second and third row transition metal ions Y(III), Ru(II/III), Ag(I), Cd(II) and Au(I) were also isolated and studied. The complexes of most lanthanide(III) ions (i.e., Pr(III), Nd(III), Sm(III), Eu(III), Gd(III), Tb(III), Dy(III), Ho(III) and Yb(III)) have also been found in the literature. From the s- and p-block of the periodic table, few Mg(II) and Sn(IV) compounds have also been reported.

All of these metal complexes were structurally characterized mainly using single-crystal X-ray crystallography and some basic spectroscopic and physicochemical techniques. Most of the lanthanide(III) complexes were studied for their photochemical properties, while many transition metal complexes, especially of the NSAID naproxen, were monitored for their potential biological potency.

## 3. Structures of the Complexes

The main features of the structures of the reported complexes are the coordination mode of the carboxylato ligands under study and the nuclearity of the resultant metal complexes, as well as the geometry around the central metal ions and the connectivity between them in the non-mononuclear compounds.

For the purposes of the present study, the reported complexes were categorized based on (i) the metal nuclearity and (ii) the coordination mode of the acetato ligands. Most of the complexes were either mononuclear or dinuclear. Among the polynuclear complexes, structures for trinuclear, tetranuclear, hexanuclear and polymeric complexes have been reported in the literature. Considering the acetato ligands, diverse (either one or more than one) coordination modes have been observed in each complex.

### 3.1. Coordination of the Carboxylato Ligands

**Figure 14:**
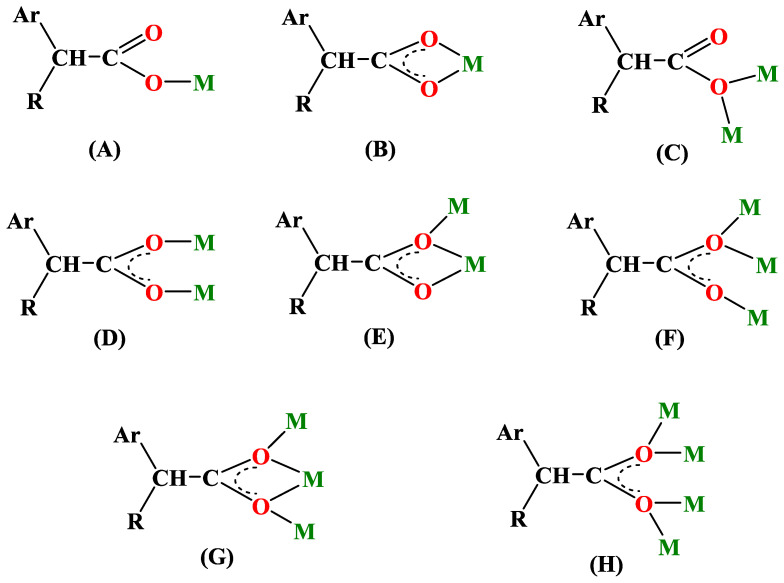
General coordination modes of the carboxylato ligands. (Ar = naphthalene or pyrene ring, R = H or Me). (**A**) Monodentate (κ–O). (**B**) Bidentate chelating (κ–O,O′). (**C**) Bidentate bridging two metal ions (µ_2_–O,O). (**D**) Bidentate bridging two metal ions (µ_2_–O,O′). (**E**) Tridentate bridging (µ_2_–O,O,O′). (**F**) Tridentate bridging three metal ions (µ_3_–O,O,O′). (**G**) Tetradentate bridging three metal ions (µ_3_–O,O,O′,O′). (**H**) Tetradentate bridging four metal ions (µ_4_–O,O,O′,O′).

Concerning the coordination of the four acids studied herein in their coordination compounds, their carboxylate group is deprotonated. The potential coordination to the metal ions may be summarized to the different binding modes which are typical for carboxylato ligands, shown in Figure 14.

They may be bound in the following ways: (i) monodentately via a carboxylato oxygen atom (κ–O) (Figure 14A), (ii) chelating bidentately via the two carboxylato oxygen atoms (κ–O,O′) (Figure 14B), (iii) bridging bidentately via one carboxylato oxygen atom (µ–O,O) forming a monoatomic bridge (Figure 14C), (iv) bidentately bridging two metal ions via the two carboxylato oxygen atoms (µ–O,O′) forming a M–O–C–O–M’ bridge (Figure 14D), (v) tridentately bridging two metal ions via the two carboxylato oxygen atoms (µ_2_–O,O,O′) forming a M–O–C–O′ chelate ring and a M–O–M’ bridge (Figure 14E), (vi) tridentately bridging three metal ions via the two carboxylato oxygen atoms (µ_3_–O,O,O′) forming a M–O–M’ bridge and a M’–O–C–O–M’’ bridge (Figure 14F), (vii) tetradentately bridging three metal ions via the two carboxylato oxygen atoms (µ_3_–O,O,O′,O′) (Figure 14G) and (viii) tetradentately bridging four metal ions via the two carboxylato oxygen atoms (µ_4_–O,O,O′,O′) (Figure 14H). Of the eight potential binding modes, the first six fashions (i)–(vi) were found in the crystal structures reported in the literature and will be presented in the corresponding section.

### 3.2. Mononuclear Complexes

Many of the complexes reported in the literature are mononuclear and contain transition metal ions (Mn(II), Co(II), Ni(II), Cu(II), Zn(II), Ag(I), Cd(II) and Au(I)), lanthanide ions (Gd(III), Eu(III) and s-block (Mg(II)) and p-block metal ions (Sn(II)) [41,42,43,44,45,46,47,48,49,50,51,52,53,54,55,56,57,58,59,60,61,62,63,64].

**Figure 15:**
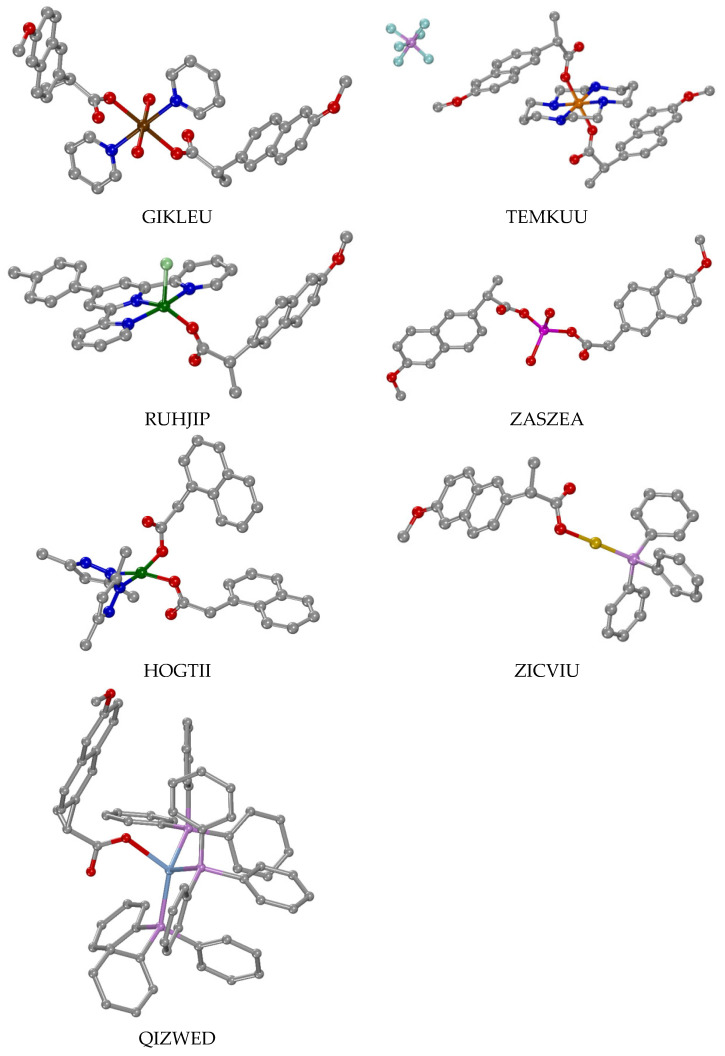
Molecular structures of selected complexes bearing monodentate ligands. GIKLEU = [Mn(NAP–O)_2_(py)_2_(H_2_O)_2_] [42], TEMKUU = [Co(NAP–O)_2_(cyclam)] [44], RUHJIP = [Cu(NAP–O)(L1)Cl] [49], ZASZEA = [Zn(NAP–O)_2_(H_2_O)_2_]·H_2_O [46], ZICVIU = [Au(NAP–O)(PPh_3_)] [51], HOGTII = [Cu(N1A–O)_2_(Hdmpz)_2_] [45] and QIZWED = [Ag(NAP–O)(PPh_3_)_3_]·H_2_O [50]. Hydrogen atoms, disordered atoms and solvate molecules are omitted for clarity reasons. Color code for atoms: C: gray, O: red, N: blue, Mn: brown, Co: orange, P: violet, F: light sea-blue, Cu: green, Cl: light green, Zn: magenta, Au: gold-yellow and Ag: cyan. The figures of the structures were adapted from the corresponding references.

**Figure 16:**
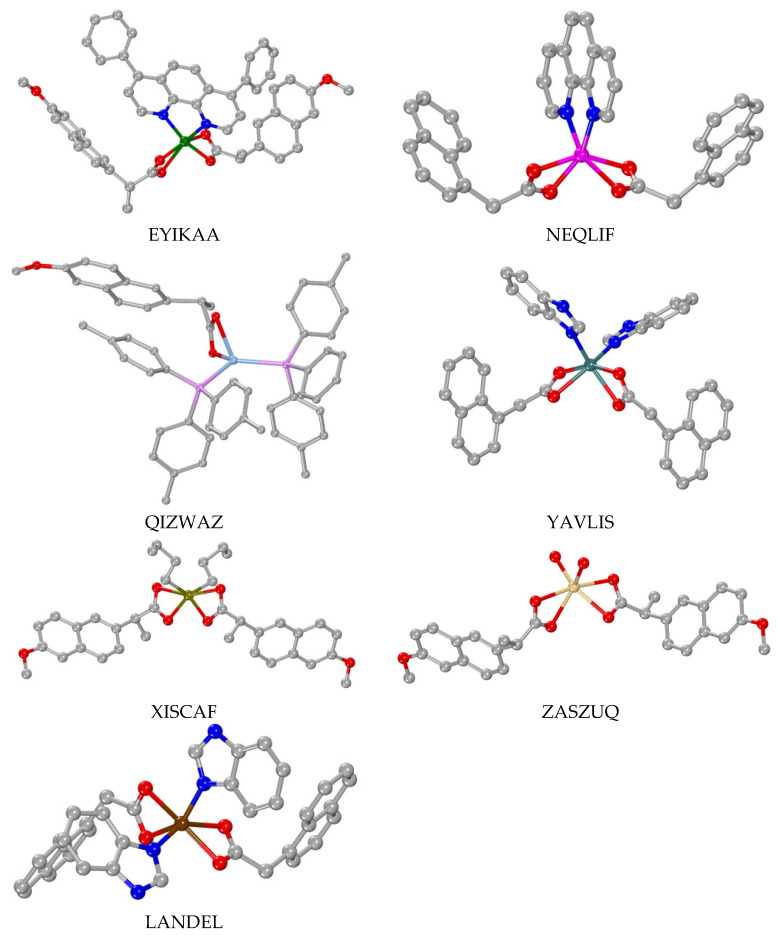
Molecular structures of selected complexes bearing bidentate chelating ligands. EYIKAA = [Cu(NAP–O,O′)_2_(4,7–diPhphen)] [55], NEQLIF = [Zn(N1A–O,O′)_2_(phen)] [57], QIZWAZ = [Ag(NAP–O,O′)(tptp)_2_] [50], YAVLIS = [Ni(N1A–O,O′)_2_(Hbzmd–N3)_2_]·H_2_O [54], XISCAF = [(*n*–Bu)_2_Sn(NAP–O,O′)_2_] [59], ZASZUQ = [Cd(NAP–O,O′)_2_(H_2_O)_2_]·H_2_O [46] and LANDEL= [Mn(N1A–O,O′)_2_(Hbzmd)_2_]·H_2_O [53]. Hydrogen atoms, disordered atoms and solvate molecules are omitted for clarity reasons. Color code for atoms: C: gray, O: red, N: blue, Mn: brown, Cu: green, P: violet, Zn: magenta, Ag: cyan; Cd: yellow, Ni: petrol green and Sn: dark yellow. The figures of the structures were adapted from the corresponding references.

**Figure 17:**
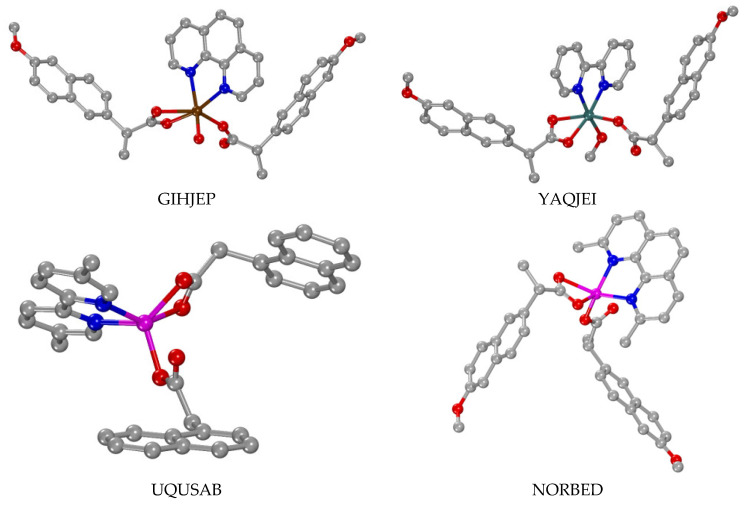
Molecular structures of selected complexes bearing monodentate and bidentate chelating ligands. GIHJEP = [Mn(NAP–O)(NAP–O,O′)(phen)(H_2_O)] [42]; YAQJEI = [Ni(NAP–O)(NAP–O,O′)(bipy)(H_2_O)] [61]; UQUSAB = [Zn(N1A–O)(N1A–O,O′)(5,5′–Me_2_–bipy)] [64] and NORBED = [Zn(NAP–O,O′)_2_(neoc)] [63]. Hydrogen atoms, disordered atoms and solvate molecules are omitted for clarity reasons. Color code for atoms: C: gray, O: red, N: blue, Mn: brown, Zn: magenta and Ni: petrol green. The figures of the structures were adapted from the corresponding references.

In most of the mononuclear complexes, the carboxylato group is monodentately bound to the metal ion (Table 1, part I), representative complexes are shown in Figure 15). However, in many of the reported mononuclear complexes, the carboxylato group of the ligands is bidentately chelating and coordinated to the metal ion (Table 1, part II), complexes shown representatively in Figure 16). In addition, in five complexes [42,61,62,63,64], a combination of these two binding modes was observed; one ligand was monodentately bound and the second one was bidentately chelating and coordinated (Table 1, part III), examples shown in Figure 17). Almost all of these mononuclear complexes were neutral with the exception of the cationic complex [Cu(N1A–O)(EDA)_2_](ClO_4_) [47].

All of these mononuclear complexes are heteroleptic and the coordination spheres of the central metal ions are completed by oxygen atoms of the N1A^−^, N2A^−^ or NAP^−^ ligands and O, N, P or C atoms derived from appropriate co-ligands that served as O–donors (H_2_O), N–donors (Figure 5, Figure 6, Figure 7, Figure 8, Figure 9, Figure 10 and Figure 11), P–donors (triphenylphosphine or tri(p–tolyl)phosphine) or C–donors (*n*–butyl), respectively.

The coordination number (CN) of the central metal ion and subsequently its geometry differs and is dependent on the nature of the metal. It varies from two (for Au(I) in complex [Au(NAP–O)(PPh_3_)] [51]) up to eight (for the lanthanide ions Gd(III) and Eu(III) in co-crystallized complexes [Gd(N1A–O)_2_(phen)_2_(H_2_O)_2_]·[Eu(N1A–O)_2_(phen)_2_(H_2_O)_2_](N1A)_2_·2H_2_O [52]). Considering the geometry around the central metal ions, diverse cases were observed depending on their coordination number. For CN = 2, a linear arrangement of the ligands was observed in complex [Au(NAP–O)(PPh_3_)] [51]. For CN = 4 (coordination sphere: MN_3_O, MOP_3_, MN_2_O_2_, MO_2_P_2_ and MO_4_), distorted tetrahedral geometry was observed for the Zn(II) and Ag(I) ions and distorted square pyramidal geometry was observed for the Cu(II) ions in their complexes. Five-coordinate metal ions (coordination sphere: MN_2_O_3_, MN_3_OCl, MN_4_O and MO_5_) were observed in Cu(II) and Zn (II) complexes bearing a distorted square pyramidal geometry around the metal ions. Distorted octahedral geometry was observed for six-coordinate metal ions resulting from a variety of coordination environments (MC_2_O_4_, MN_4_O_2_, MN_2_O_4_ and MO_6_). In the case of the lanthanide(III) complex, eight-coordination was observed (MN_4_O_4_) resulting in a distorted dodecahedral geometry.

### 3.3. Dinuclear Complexes

Significant also is the number of the dinuclear complexes with 1–naphthylacetato, 2–naphthylacetato, naproxen and 1–pyreneacetato ligands found in the literature (Table 2). Complexes of the first-row transition metal ions Fe(III), Cu(II) and Zn(II) and the second-row transition metal ions Y(III), Ru(II)/Ru(III) and Cd(II) were reported, as well as those of the lanthanide ions Pr(III), Nd(III), Sm(III), Eu(III), Gd(III), Tb(III), Dy(III) and Yb(III) [35,48,65,66,67,68,69,70,71,72,73,74,75,76,77,78,79,80,81,82,83,84,85]. Most of the complexes are neutral, while a few of them are cationic and complex K[Ru_2_(µ_2_–N2A–O,O′)_2_(dhpta)] is an anionic Ru(II) complex [72].

In all of these complexes, the 1–naphthylacetato, 2–naphthylacetato, naproxen and 1–pyreneacetato ligands are the principal bridging ligands and, in some cases, other bridging co-ligands (µ–O and µ–OH) are also present [35,48,65,66,67,68,69,70,71,72,73,74,75,76,77,78,79,80,81,82,83,84]. The exception of [Zn_2_(μ–dmpz)_2_(Hdmpz)_2_(N1A–O)_2_] should be noted, where the dmpz^−1^ (Hdmpz = 3,5–dimethylpyrazole) co-ligands have the bridging role between the two Zn(II) and the N1A^−1^ ligands are terminal monodentate ligands [85].

Regarding the bridging mode of the concerned ligands, three different bridging cases of those shown in Figure 14C–E have been observed either alone or in combination with other κ or µ modes. The number of the oxygen atoms occupying the coordination sites is high and the coordination spheres of the metal ions is mainly completed by O–donors (oxo or hydroxo bridges or solvent ligands such as H_2_O, DMF, DMSO and THF), N–donors (Figure 5, Figure 6, Figure 7, Figure 8, Figure 9, Figure 10 and Figure 11) or P–donors (PPh_3_).

**Figure 18:**
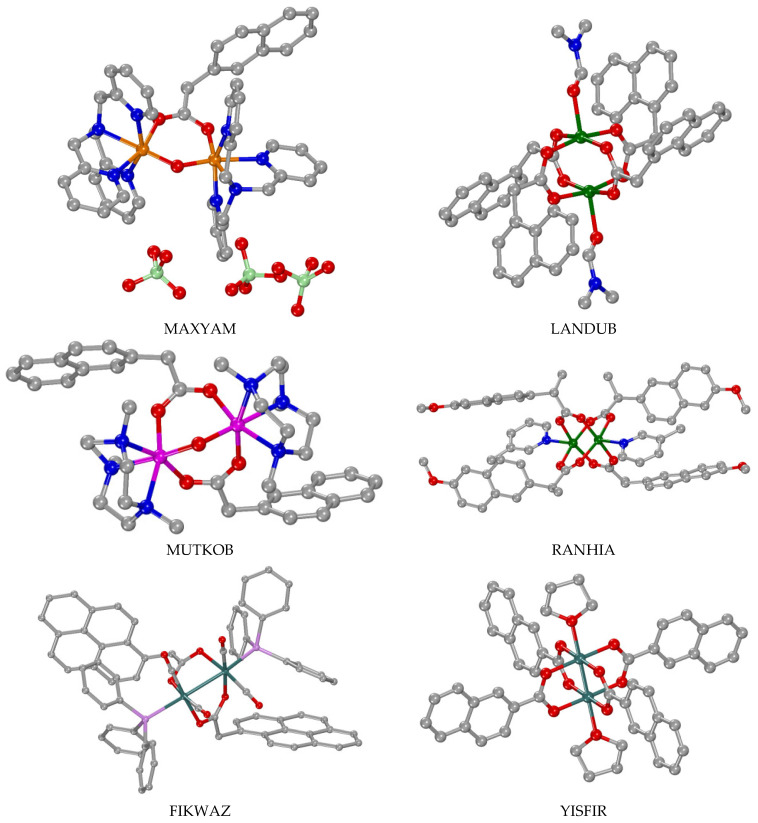
Molecular structures of selected complexes bearing only bidentate bridging (μ–O,O’) ligands. MAXYAM = [Fe_2_(µ_2_–O)(µ_2_–N2A–O,O′)(TPA)_2_](ClO_4_)_3_ [65]; LANDUB = [Cu_2_(µ_2_–N2A–O,O′)_4_(DMF)_2_] [68]; MUTKOB = [Zn_2_(µ_2_–OH)(µ_2_–N2A–O,O′)_2_(TACN–Me_3_)_2_](ClO_4_) [66]; RANHIA = [Cu_2_(μ_2_–NAP–O,O′)_4_(3pic)_2_] [48]; YISFIR = [Ru_2_(µ_2_–N2A–O,O′)_4_(H_2_O)_2_](PF_6_)·THF [71] and FIKWAZ = [Ru_2_(µ_2_–PYA–O,O′)_2_(CO)_4_(PPh_3_)_2_] [35]. Hydrogen atoms, disordered atoms and solvate molecules are omitted for clarity reasons. Color code for atoms: C: gray, O: red, N: blue, Co: orange; Cu: green; Cl: light green; Zn: magenta; P: pink and Ru: petrol green. The figures of the structures were adapted from the corresponding references.

Concerning the coordination mode of the carboxylato ligands in these complexes, five cases/combinations have been observed (Table 2). In many of the dinuclear complexes, the carboxylato ligands are coordinated only in a µ–O,O′ mode (Figure 14D) acting as O–C–O bridges (Table 2, part I) with the number of bridges varying from one to four. In the case of four bridges, the dinuclear complexes (all reported Cu(II) complexes, i.e., [Cu_2_(µ_2_–N2A–O,O′)_4_(DMSO)_2_]·2(HN1A)·2DMSO [67], [Cu_2_(µ_2_–N2A–O,O′)_4_(DMF)_2_] [68], [Cu_2_(μ_2_–NAP–O,O′)_4_(3pic)_2_] [48] and [Cu_2_(μ_2_–NAP–O,O′)_4_(caf)_2_] [69], the Zn(II) complex [Zn_2_(µ_2_–N2A–O,O′)_4_(phdat)_2_] [70] and the mixed-valence Ru(II)/Ru(III) complexes [Ru_2_(µ_2_–N2A–O,O′)_4_(H_2_O)_2_](PF_6_) and [Ru_2_(µ_2_–N1A–O,O′)_4_(THF)_2_](PF_6_) [71]) adopt the paddlewheel arrangement around the metal atoms (Figure 18). For the dinuclear Fe(III) complexes, an additional µ_2_–O bridge was also found [65,66], while in complex [Zn_2_(µ_2_–OH)(µ_2_–N2A–O,O′)_2_(TACN–Me_3_)_2_](ClO_4_), a hydroxo bridge was reported [66].

**Figure 19:**
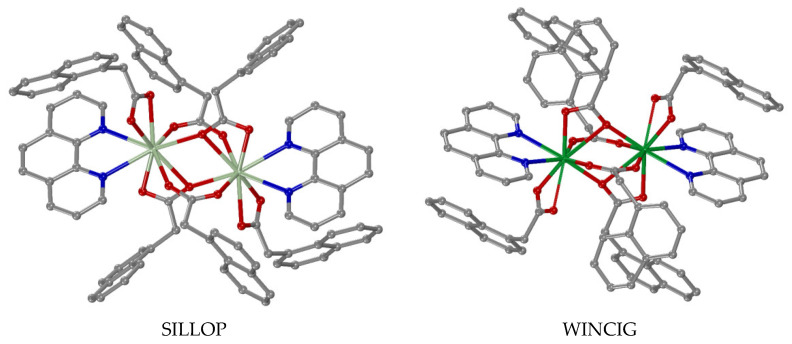
Molecular structures of selected complexes bearing two tridentate bridging (μ–O,O,O′), two bidentate bridging (μ–O,O’) and two bidentate chelating (κ–O,O′) ligands. SILLOP = [Pr_2_(µ_2_–N1A–O,O,O′)_2_(µ_2_–N1A–O,O′)_2_(κ–N1A–O,O′)_2_(phen)_2_]·DMF [73] and WINCIG = [Yb_2_(µ_2_–N1A–O,O,O′)_2_(µ_2_–N1A–O,O′)_2_(κ–N1A–O,O′)_2_(phen)_2_]·DMF [73]. Hydrogen atoms, disordered atoms and solvate molecules are omitted for clarity reasons. Color code for atoms: C: gray, O: red, N: blue, Yb: green and Pr: light green. The figures of the structures were adapted from reference [73].

**Figure 20:**
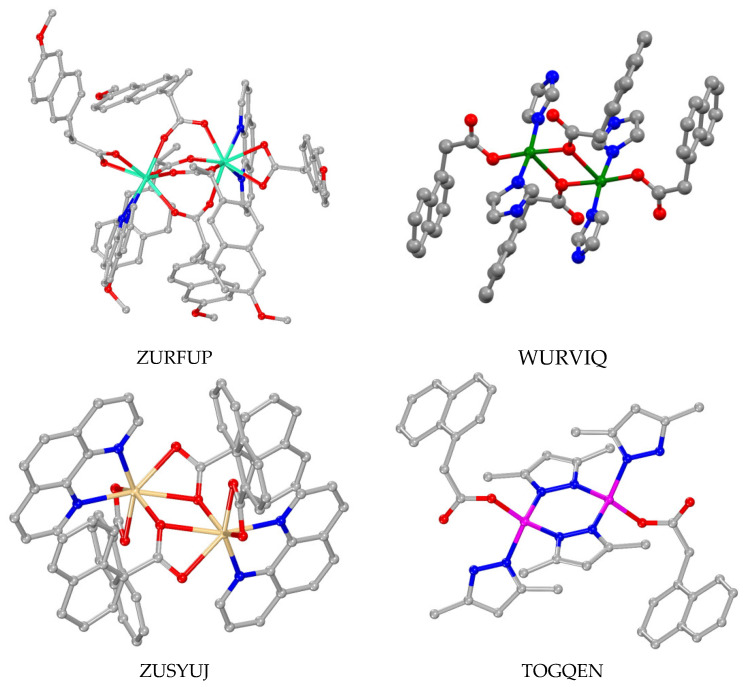
Molecular structures of selected complexes with coordination modes III–VI of Table 2. ZURFUP = [Dy_2_(µ_2_–NAP–O,O′)_4_(NAP–O,O′)_2_(phen)_2_] (mode III: bidentate bridging (μ–O,O’) and bidentate chelating) [82]; WURVIQ = [Cu_2_(µ_2_–N1A–O,O)_2_(N1A–O)_2_(Himi)_4_] (mode IV: bidentate bridging (μ–O,O) and monodentate binding) [83]; ZUSYUJ = [Cd_2_(µ_2_–N1A–O,O,O′)_2_(κ–N1A–O,O′)_2_(phen)_2_] (mode V: tridentate bridging (μ–O,O,O′) and bidentate chelating) [84] and TOGQEN = [Zn_2_(μ–dmpz)_2_(Hdmpz)_2_(N1A–O)_2_] (mode VI: monodentate binding, bridging from other co-ligand) [85]. Hydrogen atoms, disordered atoms and solvate molecules are omitted for clarity reasons. Color code for atoms: C: gray, O: red, N: blue, Cd: yellow; Cu: green; Dy: light green and Zn: magenta. The figures of the structures were adapted from the corresponding references.

Another significant finding is the number of the lanthanide(III) complexes of the general formula [M_2_(µ_2_–N1A–O,O,O′)_2_(µ_2_–N1A–O,O′)_2_(κ–N1A–O,O′)_2_(phen)_2_]·DMF [73,74,75,76,77,78,79,80,81], whereby they exhibit three different binding modes; two of the four N1A ligands are tridentate-bound forming µ_2_–O,O,O′ bridges, two are bidentate in a µ_2_–O,O′ bridging mode and the other two ones are in the bidentate chelating (κ–O,O′) mode (Table 2, part II) (Figure 19). For the lanthanide complexes [Gd_2_(µ_2_–NAP–O,O′)_4_(NAP–O,O′)_2_(phen)_2_] and [Dy_2_(µ_2_–NAP–O,O′)_4_(NAP–O,O′)_2_(phen)_2_], a combination of four µ_2_–O,O′ bridging and two bidentate chelating naproxen ligands was reported [82] (Table 2, part III) (Figure 20). There are also two unique examples: (i) [Cu_2_(µ_2_–N1A–O,O)_2_(N1A–O)_2_(Himi)_4_] [83] bearing a combination of two µ_2_–N1A–O,O monoatomic bridges and two monodentate N1A ligands (Table 2, part IV) and (ii) [Cd_2_(µ_2_–N1A–O,O,O′)_2_(κ–N1A–O,O′)_2_(phen)_2_] [84], having two tridentate µ_2_–O,O,O′ bridging and two bidentate chelating N1A ligands (Table 2, part V) (Figure 20).

The coordination number of the metal ions varies from five to six in the case of the transition metal ions and from eight to nine in the case of the lanthanide ions, and the metal ions adapt the corresponding geometries.

### 3.4. Polynuclear Complexes

Few crystal structures of polynuclear metal complexes with 1–naphthylacetato, 2–naphthylacetato and naproxen ligands were reported in the literature with the number of the metal ions being three, four and six (Table 3). In all of these complexes except complex NIHVEI, these ligands have a bridging role always following the µ–O,O′ bidentate bridging motif (Table 3) [59,84,86,87,88,89], while in some cases they are found in the µ_2_–O,O,O′ tridentate bridging mode and monodentate terminal fashion as in complex FUVZED [84] or in the bidentate bridging µ_2_–O,O mode as in complex OMOLIN [88]. In complex [Μn_6_(µ_3_–NAP–O,O,O′)(µ_2_–Hsal–O,O′)(µ_2_–shi–N,O)_5_(py)_6_], the naproxen ligand is in a rather rare µ_3_–O,O,O′ coordination mode (Figure 14(F)) bridging three manganese ions [90].

**Figure 21:**
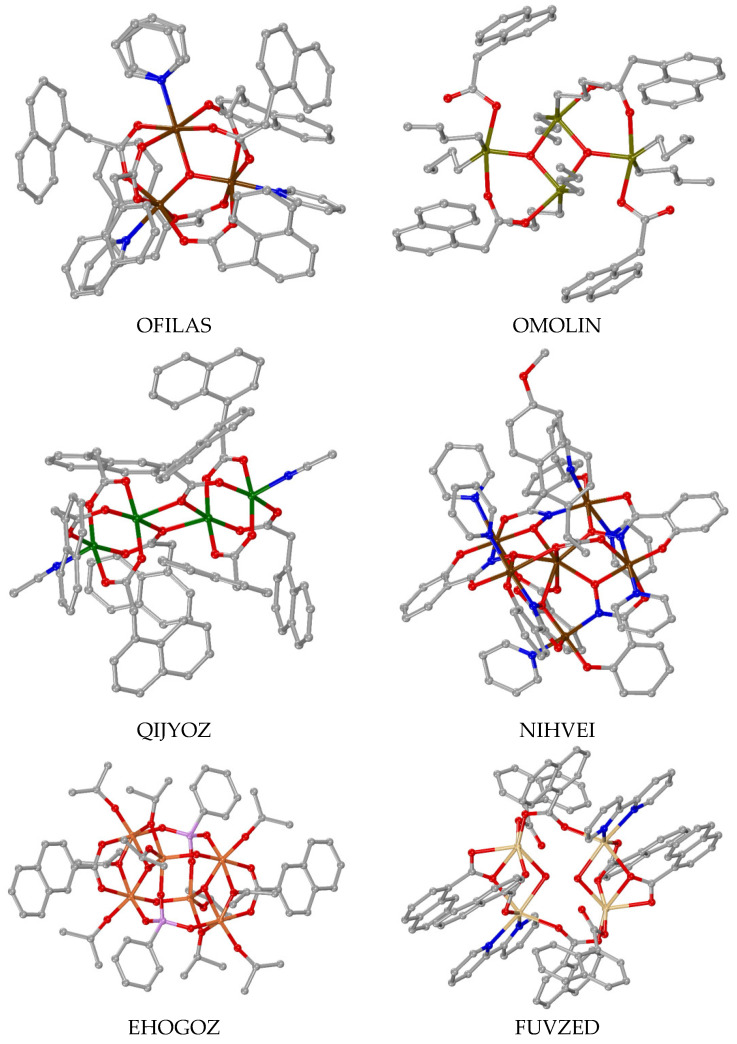
Molecular structures of selected complexes of Table 3. OFILAS = [Mn_3_(µ_3_–O)(µ_2_–N1A–O,O′)_6_(py)_3_] [86]; OMOLIN = {[(*n*–Bu)_2_Sn]_2_(µ_2_–N1A–O,O′)(µ_2_–N1A–O,O) (µ_3_–O)}_2_ [88]; QIJYOZ = [Cu_4_(µ_2_–N1A–O,O′)_6_(µ_2_–N1A–O,O,O′)_2_(CH_3_CN)_2_] [87]; NIHVEI = [Μn_6_(µ_3_–NAP–O,O,O′)(µ_2_–Hsal–O,O′)(µ_2_–shi–N,O)_5_(py)_6_] [90]; EHOGOZ = [Ti_6_(μ_3_–O)_2_(μ_2_–O)_2_(μ_3_–phenyl–phosphonato)_2_(μ_2_–isopropoxo)_4_(isopropoxo)_6_(μ_2_–N2A–O,O′)_2_] [89] and FUVZED = [Cd_4_(µ_2_–N1A–O,O′)_4_(µ_2_–N1A–O,O,O′)_2_(N1A–O)_2_(µ_2_–H_2_O)_2_(bipy)_2_] [84]. Color code for atoms: C: gray, O: red, N: blue, Mn: brown; Sn: dark yellow; Cu: green; Ti: orange; P: pink and Cd: yellow. The figures of the structures were adapted from the corresponding references.

With the exception of [(Me_3_Sn)_4_(µ_2_–NAP–O,O′)_4_] [59] which has only naproxen bridges, the co-existence of other bridging ligands is observed and they contributed to higher than two nuclearity. Diverse bridging co-ligands in diverse modes were also reported: (i) oxo ligands bridging three metal ions (µ_3_–O) as in the trinuclear mixed-valence manganese complex [Mn_3_(µ_3_–O)(µ_2_–N1A–O,O′)_6_(py)_3_] [86], the tetranuclear complex {[(*n*–Bu)_2_Sn]_2_(µ_2_–N1A–O,O′)(N1A–O,O′)(µ_3_–O)}_2_ [88] and the hexanuclear complex [Ti_6_(μ_3_–O)_2_(μ_2_–O)_2_(μ_3_–phenyl–phosphonato)_2_(μ_2_–isopropoxo)_4_(isopropoxo)_6_(μ_2_–N2A–O,O′)_2_] where it also exists in the µ_2_–O mode [89], (ii) aqua ligands bridging two Cd(II) ions in [Cd_4_(µ_2_–N1A–O,O′)_2_(µ_2_–N1A–O,O,O′)_2_(µ_2_–H_2_O)_2_(N1A–O)_2_(bipy)_2_] (bipy = 2,2′–bipyridine) [84], (iii) μ_3_–phenyl–phosphonato and (iv) μ_2_–isopropoxo ligands in the hexanuclear complex EHOGOZ [89], and (v) (µ_2_–Hsal–O,O′) and (vi) (µ_2_–shi–N,O) ligands in the complex NIHVEI [90] (Figure 21).

Considering the structural features of these seven complexes, different arrangements of the metal ions were found resulting from differences in the nuclearity and the nature of metal ions and co-ligands. More specifically, the three manganese ions in the complex [Mn_3_(µ_3_–O)(µ_2_–N1A–O,O′)_6_(py)_3_] are located in a triangular arrangement with the µ_3_–O atom in the center of the triangle [86]. In the tetranuclear copper(II) complex [Cu_4_(µ_2_–N1A–O,O′)_6_(µ_2_–N1A–O,O,O′)_2_(MeCN)_2_], each pair of copper(II) ions adapted the paddlewheel arrangement of the type [Cu_2_(µ_2_–N1A–O,O′)_3_(µ_2_–N1A–O,O,O′)(CH_3_CN)] and are joined via the two tridentate µ_2_–N1A–O,O,O′ ligands providing a dimer structure of dimers [87]. The tetranuclear Cd(II) complex [Cd_4_(µ_2_–N1A–O,O′)_4_(µ_2_–N1A–O,O,O′)_2_(µ_2_–H_2_O)_2_(bipy)_2_] could be described as a dimer of dimers since two µ_2_–N1A–O,O′ ligands behave as bridges between the two dinuclear moieties of the type [Cd_2_(µ_2_–N1A–O,O′)(µ_2_–N1A–O,O,O′)(µ_2_–H_2_O)(bipy)] [84]. [(Me_3_Sn)_4_(µ_2_–NAP–O,O′)_4_] could be characterized as a 16–metallocoronate–4 complex since a cyclic arrangement of the Sn(IV) ions bridged by the naproxen ligands was observed [59]. The arrangement of the tin ions in complex {[(*n*–Bu)_2_Sn]_2_(µ_2_–N1A–O,O′)(µ_2_–N1A–O,O)(µ_3_–O)}_2_ is circular with two µ_2_–N1A–O,O′ and two µ_2_–N1A–O,O bridges forming a [Sn–O–C–O–Sn–O] repeating unit which is further stabilized by two encapsulated µ_3_–O atoms [88].

In the case of the hexanuclear titanium complex EHOGOZ, the structure could be described as a dimer of trimers of the type [Ti_3_(μ_3_–O)(μ_2_–isopropoxo)_2_(isopropoxo)_3_(μ_2_–N2A–O,O′)] which are held together by two μ_2_–O and two μ_3_–phenyl–phosphonato bridging ligands [89]. The hexanuclear complex [Μn_6_(µ_3_–NAP–O,O,O′)(µ_2_–Hsal–O,O′)(µ_2_–shi–N,O)_5_(py)_6_] is better described as a metallacrown (MC) complex Mn(II)(µ_3_–NAP–O,O,O′)(µ_2_–Hsal–O,O′)[15–MC_Mn(III)N(shi)_–5](py)_6_. In this complex, five Mn(III) and five triply deprotonated shi^3–^ bridging ligands are located in a close circular arrangement with five [Mn–N–O] repeating units forming a 15-membered MC ring of the type [15–MC–5] that can encapsulate the sixth Mn(II) ion which is further stabilized by the µ_3_–naproxen–O,O,O′ and µ_2_–salicylato–O,O′ bridges with the MC Mn(III) ions [90].

### 3.5. Polymeric Complexes

**Figure 22:**
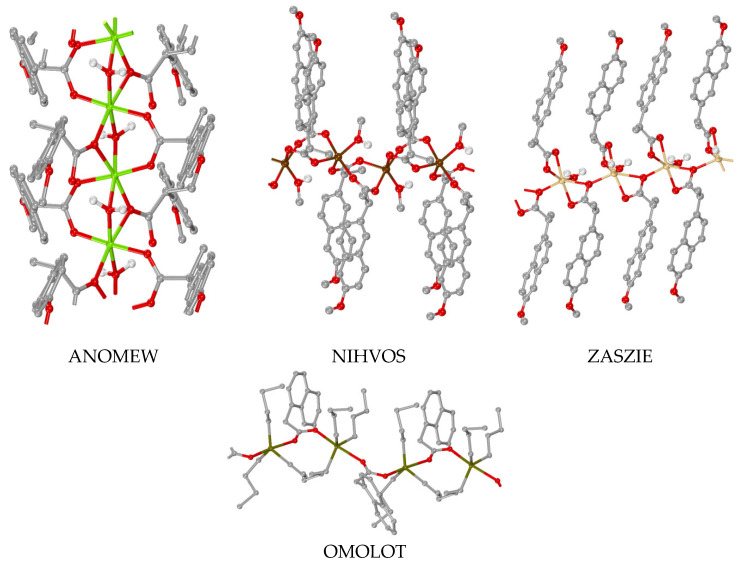
Molecular structures of selected complexes of Table 4 bearing 1–naphthylacetato and naproxen ligands as bridging ligands. ANOMEW = [Mg(µ_2_–NAP–O,O)(µ_2_–NAP–O,O′)(µ_2_–H_2_O)]_n_ [41]; NIHVOS = [Mn(µ_2_–NAP–O,O′)_2_(CH_3_OH)]_n_ [90]; ZASZIE = [Cd(NAP–O,O′)(µ_2_–NAP–O,O,O′)(H_2_O)]_n_ [46] and OMOLOT = [(*n*–Bu)_3_Sn(µ_2_–N1A–O,O,O′)]_n_ [88]. Color code for atoms: C: gray, O: red, Mg: light green, Mn: brown; Cd: yellow and Sn: dark yellow. The figures of the structures were adapted from the corresponding references.

The polymeric complexes containing 1–naphthylacetato and naproxen ligands are summarized in Table 4 and may be categorized in two groups: (I) the polymeric complexes that are polymerized via the 1–naphthylacetato and naproxen ligands [41,46,59,88,90,91,92,93] (Table 4, part I), Figure 22) and (II) the polymeric complexes that are polymerized via other co-ligands, and the 1–naphthylacetato and naproxen ligands are just found in the coordination sphere [84,94,95] (Table 4, part II). Most of the reported polymeric complexes bear a monomeric complex as the basis of the polymer with the exception of complexes FUVZIH and CAVHOA which have a trinuclear Cd(II) and a tetranuclear Ag(I) complex, respectively, as the repeating unit of the polymeric structure.

In the first group of the complexes, where the 1–naphthylacetato and naproxen ligands are the polymerizing linkers (Table 4, part I), the ligands are in either bidentate µ_2_–O,O′ or tridentate µ_2_–O,O,O′ bridging modes. Only in the case of complex [Mg(µ_2_–NAP–O,O)(µ_2_–NAP–O,O′)(µ_2_–H_2_O)]_n_, three different linkers were found, i.e., bidentate µ_2_–NAP–O,O, bidentate µ_2_–NAP–O,O′ and µ_2_–H_2_O [41]. Besides the role of the polymerizing linker, there is a bidentate chelating naproxen ligand in complex [Cd(NAP–O,O′)(µ_2_–NAP–O,O,O′)(H_2_O)]_n_ [46] and two bidentate µ_2_–O,O′ naproxen ligands which are forming bridges between the Ag(I) of the repeating tetranuclear unit in complex CAVHOA [93].

In the second group of the complexes, i.e., complexes polymerized via the other co–ligands (Table 4, part II), the 1–naphthylacetato and naproxen ligands are bound in the central metal ion either monodentately as in complexes [Zn(NAP–O)_2_(µ–3U)]_n_ and [Zn(NAP–O)_2_(µ–4U)]_n_ [94] and [Ag(N1A–O)(µ–bpp–*N,N*′)]_n_ [84] or in a bidentate chelating mode as in complex [Cd(µ_3_–pyr3O)(N1A–O,O′)(H_2_O)]_n_ [95]. The polymerization in these complexes takes place via the 1,3–dipyridin–3–ylurea, 1,3–dipyridin–4–ylurea, pyridin–3–ol and 1,3–bis(4–pyridyl)propane ligands, respectively. In the case of complex FUVZIH, the basic unit is a trinuclear Cd(II) complex where every two of the six 1–naphthylacetato ligands are found in three different binding modes in the trinuclear complex (µ_2_–N1A–O,O′, µ_2_–N1A–O,O,O′ and κ–N1A–O,O′), while the polymerization takes place via the 4,4′–bipyridine ligands [84].

## 4. Spectroscopic and Physicochemical Characterization of the Metal Complexes

Besides the X-ray crystal structures, the reported metal complexes were also studied and characterized using various (spectroscopic and physicochemical) techniques, and their use mainly depended on the nature of the metal ion. The most often reported spectroscopic techniques are IR, NMR, photochemical (UV–vis, fluorescence), EPR and Mössbauer spectroscopies. Other techniques employed for the characterization of the complexes include thermal analysis, magnetic measurements and electrochemistry.

### 4.1. IR Spectroscopy

In most cases, the IR spectra of the reported complexes were discussed as a preliminary method to assess the presence, the deprotonation and the possible coordination mode of the carboxylato ligands. In the IR spectra of the complexes, two characteristic bands of the carboxylato ligands were observed: the band located in the range 1530–1645 cm^−1^ was attributed to the antisymmetric stretching vibration (*ν*_asym_(COO)) of the deprotonated carboxylato group, while the second band located in the region 1370–1480 cm^−1^ was assigned to the symmetric stretching vibration (*ν*_sym_(COO)) of the deprotonated carboxylato group (Table S1). Their difference (= *ν*_asym_(COO) − *ν*_sym_(COO)) is known as the parameter Δ*ν*(COO) and is often used to estimate the potential coordination mode of carboxylato ligands [96]. The Δ*ν*(COO) value of each complex is compared with that of the deprotonated carboxylato ligand (usually in the form of an alkali salt) and may suggest (i) a monodentate (asymmetric) coordination, if Δ*ν*(COO)_complex_ > Δ*ν*(COO)_salt_ or (ii) a bidentate binding mode when Δ*ν*(COO)_complex_ < Δ*ν*(COO)_salt_ [97]. The limits of these values often depend on the nature of the metal ion. In addition, in the case of more than one coordination mode of the ligands (e.g., monodentate and bidentate bridging), two values for Δ*ν*(COO) may be observed. In most cases, characteristic bands concerning the presence of the corresponding co-ligands, e.g., the out-of-plane ρ(C–H) vibrations of the respective nitrogen–donor co-ligands, have also been observed and reported [42,43,47,48,49,50,56,59,60,61,63,71,73,75,90,93].

### 4.2. NMR Spectroscopy

NMR spectroscopy was mainly applied to diamagnetic complexes, i.e., Zn(II), Ag(I) and Sn(IV) complexes. In most cases, the ^1^H and ^13^C NMR spectra were recorded in solutions of the complexes. In the reported ^1^H and ^13^C NMR spectra of Ag(I) [50,93], Zn(II) [63] and Sn(IV) [59] with NAP ligands and of complex [Ru_2_(µ_2_–PYA–O,O′)_2_(CO)_4_(PPh_3_)_2_] [35], all of the expected signals of the NAP or PYA ligands and the respective co-ligands were present and slightly shifted downfield or upfield (compared with free HNAP or HPYA) as a result of their coordination to the metal ions. In general, the results were in good agreement with the determined crystal structures and proposed structures of the complexes. The absence of any additional signals related to other species revealed the stability of the complexes in solution.

In few cases, the ^31^P or ^119^Sn NMR spectra were also recorded for complexes bearing P–donor co-ligands or for tin complexes, respectively. In particular, the ^31^P{^1^H} NMR spectrum of [Ru_2_(µ_2_–PYA–O,O′)_2_(CO)_4_(PPh_3_)_2_] showed a signal characteristic for the PPh_3_ axial ligands [35]. Especially for the Sn(IV) complexes [59], the ^119^Sn NMR spectra were also recorded and gave significant information regarding the environment of Sn(IV) ions. Based on the values of the ^119^Sn NMR chemical shifts (and according to the related literature [98]), penta-coordination of Sn was suggested for five complexes (δ = −190 to −90 ppm), i.e., ([(Me_3_Sn)_4_(NAP)_4_], [((*n*–Bu)_3_Sn)_4_(NAP)_4_], [(Ph_3_Sn)(NAP)]_n_, [((PhCH_2_)_3_Sn)(NAP)]_n_ and {[Me_2_Sn(NAP)]_2_O}_2_, and the hexa-coordination of Sn was proposed for three complexes (for δ values in the range from −400 to −210 ppm), namely [((n–Bu)_2_Sn)(NAP)_2_], [(Ph_2_Sn)(NAP)_2_] and [((PhCH_2_)_2_Sn)(NAP)_2_]; these conclusions were also confirmed by the reported X-ray crystal structures [59].

### 4.3. Photochemical Properties

In order to study the photochemical behavior of the reported complexes, their electronic (UV–vis) and fluorescence spectra were recorded.

The UV–vis spectra of the transition metal complexes were recorded in solution and in solid state and were used to check whether the complexes retained their stability in solution and across time. In most of the reported cases, the discussion was focused on the visible area of the spectrum in order to characterize the bands assigned to d–d transitions of the centered metal ions.

For Co(II)–naproxen complexes, the bands assigned to ^4^Τ_1g_(F) → ^4^T_2g_, ^4^Τ_2g_(F) → ^4^A_2g_ and ^4^Τ_1g(F)_ → ^4^T_1g_(P) transitions were found in the ranges 735–740 nm, 535–570 nm and 445–450 nm, respectively [43]. For the Ni(II)–naproxen complexes, the bands attributed to the ^3^A_2g_ → ^3^T_2g_, ^3^A_2g_ → ^3^T_1g_ and ^3^A_2g_ → ^3^T_1g_(P) transitions were observed in the regions 930–1015 nm, 520–730 nm and 390–403 nm, respectively [61]. For mixed-valence manganese(II/III) complexes, the d–d transition band related to Mn(III) ions was observed at 620–635 nm [90]. For Fe(III) complexes, the band related to the d–d transitions of Fe(III) ions was found at 700–730 nm [65,66]. In the case of Cu(II) complexes, the d–d transition band of Cu(II) ions was found in the range 612–730 nm [49,56], which is dependent on the geometry of the coordination sphere around copper(II) ions [99].

Considering the photoluminescence properties of the reported complexes, the fluorescence emission spectra of complexes containing either d^10^ metal ions (i.e., Zn(II) and Cd(II)) or lanthanide(III) ions were recorded with diverse excitation wavelengths.

The emission bands recorded for complexes [Zn(NAP)_2_(H_2_O)_2_], [Cd(NAP)_2_(H_2_O)_2_] and [Cd(NAP)_2_(H_2_O)]_n_ at 360 nm (λ_excitation_ = 300 nm), 356 nm (λ_excitation_ = 280 nm) and 355 nm (λ_excitation_ = 280 nm), respectively, were assigned to intraligand (π–π*) fluorescence emissions, as the spectra of free HNAP presented emission bands at 347 nm and 372 nm (for λ_ex_ = 280 nm) [46]. Similarly, the emission bands of complexes [Cd_2_(N1A)_4_(phen)_2_] (λ_max,emission_ = 421 nm and ~575 nm for λ_excitation_ = 370 nm), [Cd_4_(N1A)_8_(bipy)_2_(H_2_O)_2_] (λ_max,emission_ = 421 nm for λ_ex_ = 370 nm), [Cd_3_(N1A)_6_(4,4′–bipy)_2_]_n_ (λ_max,emission_ = 421 nm and ~570 nm for λ_excitation_ = 345 nm) [84] and [Zn_2_(N2A)_4_(phdat)_2_] (λ_max,emission_ = 404 nm for λ_excitation_ = 304 nm) [70] were assigned to the intraligand π–π* transition of coordinated 1–naphthylacetato [84] or 2–naphthylacetato ligands [70] (λ_max,emission_ = 420 nm for λ_excitation_ = 345 nm). For the lanthanide(III) complexes, a series of emission bands were observed in the region 540–700 nm and were mainly assigned to the f–f transitions of the respective lanthanide(III) ions (Table 5) [52,73,78].

In addition, the static emission spectra of 1–naphthylacetic acid, 2–naphthylacetic acid and their Ru(III) and Mn(II/III) complexes were also reported. The Ru(III) complexes K[Ru_2_(N1A)_2_(dhpta)] and K[Ru_2_(N2A)_2_(dhpta)] exhibited fluorescence and phosphorescence in the regions 300–400 nm and 400–550 nm, respectively, when excited at 295 nm, and showed very short fluorescence lifetimes (36 ps and 540 fs, respectively) [72]. Complexes [Mn_3_(µ_3_–O)(µ_2_–N1A–O,O′)_6_(py)_3_] and [Mn_3_(µ_3_–O)(µ_2_–N2A–O,O′)_6_(py)_3_] showed fluorescence with higher fluorescence lifetimes (in the range 13–48 ns, depending on the solvent used) [86].

### 4.4. EPR Spectroscopy

EPR spectra were recorded for few Cu(II) and Co(II) naproxen complexes in powder samples as well as in frozen solution. In general, a comparison of the EPR spectra between powder samples and solutions was useful to reveal that the complexes retained their structure in solution.

The EPR spectra of powder samples of [Cu(NAP)_2_(bipy)] and [Cu(NAP)_2_(phen)] [56] and [Cu(NAP)(L1–L6)Cl] [49] showed signals with g_||_ > g_⊥_ > 2 which are close to the expected values for distorted octahedral geometry around Cu(II) ions [100]. Furthermore, the EPR spectrum of the DMSO solution of [Cu(NAP)_2_(bipy)] was similar to the powder sample verifying the geometry around Cu(II) ions, and the hyperfine pattern was resolved and gave the following: A_||_ = 400 MHz, A_⊥_ = 20 MHz, g_⊥_ = 2.06 and g_||_ = 2.26 [56]. The EPR spectrum of the dinuclear complex [Cu_2_(NAP)_4_(caf)_2_] was similar to other symmetric dimeric copper(II) species with g_⊥_ = 2.04 and g_||_ = 2.23 [69].

The EPR spectra of complex [Co(NAP)_2_(py)_2_(H_2_O)_2_] were recorded in frozen DMSO solution and in powder samples which showed similar signals (a derivative centered at g = 4.8 and a broad peak at g =1.9) at low temperatures (T < 25 K) [43], typical for high-spin Co(II) ions.

### 4.5. Mössbauer Spectroscopy

Mössbauer spectroscopy was involved in the characterization of one Sn(IV) complex. More specifically, the ^119^Sn Mössbauer spectrum of [(*n*–Bu)_2_Sn(NAP–O,O′)_2_] was recorded in solid state and it was typical for Sn complexes. The isomer shift (δ) value (1.40 mm s^−1^) showed the oxidation state of Sn(IV) and the value of the quadrupole splitting parameter (D) was 3.44 mm s^−1^, being typical for *trans*-R_2_Sn distorted octahedral complexes [60].

### 4.6. Thermal Behavior

The thermal behavior and stability of reported Cu(II), Zn(II), Ag(I) and Cd(II) complexes were mainly examined using thermogravimetric analysis (TGA) and differential thermogravimetric analysis (DTA). In addition, the decomposition steps were also investigated. The reported complexes were stable up to temperatures varying between 122 and 294 °C and this was followed by their decomposition up to three steps (Table 6). During these steps, the gradual loss of the ligands took place resulting in the formation of the corresponding metal oxide as the final product. In most cases, the mass losses found experimentally from the respective thermogravimetric curves were in good agreement with the theoretically calculated mass losses [45,47,48,70,84,85,87,93].

### 4.7. Magnetic Measurements

The magnetic properties of the paramagnetic metal complexes were often studied and reported. In most cases, especially for the mononuclear complexes, room-temperature (RT) magnetic measurements were adequate to prove their monomeric nature. For the mononuclear Co(II), Ni(II) and Cu(II) complexes as well as the dinuclear Cu(II) complexes bearing a paddlewheel arrangement of the ligands, the observed μ_eff_ values at RT were close to the spin-only (theoretically expected) values and further variable temperature (VT) magnetic measurements were not conducted [43,49,56,61,69].

VΤ magnetic measurement studies were performed for a series of polynuclear manganese(II/III), iron(III), copper(II), ruthenium(III) and lanthanide(III) complexes with 1–naphthylacetato and 2–naphthylacetato ligands. For all of the reported Mn(II/III), Fe(III), Cu(II) and Ru(III) complexes, the molecular susceptibility χ_M_ decreased upon lowering the temperature and below a temperature it increased up to a plateau value. This behavior is typical for antiferromagnetic interactions between the metal ions. The data were further fitted with appropriate equations and the corresponding g and *J* parameters were calculated as cited in Table 7. In all cases, the calculated parameters were in good agreement with previously reported data concerning complexes with similar structural motifs [65,66,72,86,87]. On the other hand, the VT magnetic behavior of the three lanthanide(III) complexes [Ln_2_(µ_2_–NAP–O,O′)_4_(NAP–O,O′)_2_(phen)_2_] (Ln = Dy, Gd and Er) may be attributed to a single-molecule magnet (SMM) behavior [82].

### 4.8. Electrochemical Behavior

In order to study the electrochemical behavior of the complexes, the cyclic voltammograms of the complexes were recorded in solution. More specifically, the cyclic voltammograms of Mn(II/III) [86], Fe(III) [65,66] and Ru(III) [72] with 1–naphthylacetato and 2–naphthylacetato ligands and Co(II) [43], Ni(II) [61] and Cu(II) [49,55] complexes with naproxen ligands were recorded in diverse solvents and the redox potentials of the observed waves were studied.

In the case of the polynuclear complexes bearing 1–naphthylacetato or 2–naphthylacetato ligands, two distinct processes were observed: (i) the reduction and oxidation of the naphthalene moiety took place at extreme potentials providing rather quasi-reversible waves (E_pc_ < −1.5 V, E_pa_ > +1.0 V), while (ii) the metal-centered redox processes (M(II) ↔ M(III)) were observed at rather close-to-zero potentials resulting in quasi-reversible or irreversible waves (Table 8).

In the cases of the mononuclear metal(II)-naproxen complexes, irreversible or quasi-reversible waves attributed to one-electron processes were observed. In all cases, the redox process [M(II)] ↔ [M(I)] was successfully assigned to E_pc_ and E_pa_ at potentials depending on the nature of metal ions (Table 9) [43,55,61].

Furthermore, five metal(II)–naproxen complexes (namely [Ni(H_2_O)_6_](NAP)_2_·2H_2_O, [Cu(NAP)_2_(H_2_O)_3_]·H_2_O, [Zn(NAP)_2_(H_2_O)_2_]·H_2_O, [Cd(NAP)_2_(H_2_O)_2_]·H_2_O and [Cd(NAP)_2_(H_2_O)]_n_ were checked for potential ferroelectric properties, since they crystallized in polar space groups which is a condition for ferroelectricity [46]. The properties examined for these complexes were remnant polarization (P_r_) (with values of 0.004–0.022 µC/cm^2^), coercive field (E_c_) (values of 0.077–1.04 kV/cm) and saturation spontaneous polarization (P_s_) (in the range of 0.006–0.046 µC/cm^2^); in combination with the low leakage currents (<10^−8^ A/cm^2^), these may confirm the ferroelectricity of the complexes.

## 5. Biological Activity

Many of the reported coordination compounds were screened in vitro for their potential biological activity. Therefore, the potential anticancer activity of selected complexes was monitored by determining their cytotoxicity against diverse cancer cell lines. The antibacterial activity of many complexes was tested against diverse microorganisms. The antioxidant capacity of many naproxen complexes was investigated against free radicals and via the inhibition of relevant enzymes. Few reports were found concerning other activities such as catechol oxidase-like activity and antimalarial activity. Complimentary to these studies, the interaction and the affinity of the reported complexes for certain biomacromolecules, i.e., DNA and albumins, were also evaluated in vitro.

### 5.1. Anticancer Activity of the Complexes

In order to evaluate the potential anticancer potency of the reported compounds, their cytotoxic efficacy was monitored in vitro against the following cancer cell lines: 4T1 (human breast cancer), A–549 (human lung carcinoma), Colo205 (human adenocarcinoma), HEK–293 (human embryonic kidney), HeLa (cervical cancer), HepG2 (human hepatoblastoma), HT–29 (human colorectal adenocarcinoma), MCF–7 (breast cancer), MDA–MB–231 (human breast cancer), MDA–MB–453 (human breast adenocarcinoma), MDA–MB–468 (human breast cancer), NCI–H460 (human large-cell lung carcinoma), HMLER cells (immortalized and transformed via retroviral expression of SV40 large T oncogene, hTERT and HrasV12) and HMLER–shEcad cells (HMLER cells subjected to Ecadherin silencing by short hairpin RNA interference), as well as against the 3T3–L1 normal cells from mouse fibroblasts. The cytotoxicity of the complexes was evaluated in comparison to their corresponding ligands and diverse reference compounds including 5–fluorouracil, capecitabin, carboplatin, cisplatin, doxorubicin and salinomycin.

The results were expressed as the concentration that inhibits the survival of 50% of the cells (IC_50_, in μM). The reported complexes (Table 10) were more active than their components and in most cases were even more active than some of the reference compounds used [44,49,50,51,62,88,90,93], which launched more elaborate studies regarding the mechanism of cancer cell death. Among the tested complexes, the most active ones were found: (i) [Μn_6_(NAP)(Hsal)(shi)_6_(py)_6_] (IC_50_ = 9.6 ± 0.3 μM) [90], [Cu(NAP)(L1)Cl] (IC_50_ = 1.51 ± 0.15 μM) [49], [Ag(NAP)(tptp)_2_] (IC_50_ = 2.2 ± 0.2 μM) and [Ag(NAP)(PPh_3_)_3_](H_2_O) (IC_50_ = 0.7 ± 0.1 μM) [50] against MCF–7 cells, (ii) [Co(NAP)_2_(cyclam)] [44] and [Au(NAP–O)(PPh_3_)] [51] against HMLER cells (IC_50_ = 0.183–0.43 μM) and HMLER–shEcad cells (IC_50_ = 0.063–0.11 μM) and (iii) {[(*n*–Bu)_2_Sn]_2_(N1A)_2_(O)}_2_ against HeLa, HepG2 and Colo205 cells (IC_50_ = 0.100–1.805 μM) [88] and the polymeric complex [(*n*–Bu)_3_Sn(N1A)]_n_ against all cell lines tested (IC_50_ = 0.104–0.361 μM) [88].

More elaborate studies concerning the potential anticancer activity of the aforementioned compounds were also conducted leading to noteworthy results. In vitro experiments concerning the combinatory activity of [Μn_6_(NAP)(Hsal)(shi)_6_(py)_6_] with the well-known chemotherapeutic drugs irinotecan, cisplatin, paclitaxel and 5–fluorouracil against MCF–7, A549 and HeLa cell lines have shown better cytotoxic activity of each combination checked in comparison to its free components, revealing that a combination of drugs may have superior activity [90].

The association of cytotoxic effects of several reported complexes on the cancer cell lines with apoptosis was studied using diverse techniques (e.g., cell morphology studies, DNA laddering and Annexin V–FITC/PI double staining). Complexes [Cu(NAP)(L1)Cl], [Cu(NAP)(L2)Cl] and [Cu(NAP)(L3)Cl] can initiate early apoptosis on MCF–7 cells with [Cu(NAP)(L3)Cl] being the most active complex that induced apoptosis up to 84% of the cancer cells [49]. The silver(I)–naproxen complexes [Ag(NAP)(PPh_3_)_3_](H_2_O) and [Ag(NAP)(tptp)_2_] may also induce apoptosis on MCF–7 cells as proposed by internucleosomal fragmentation studies [50]. The polymeric silver(I)–naproxen complex [Ag_4_(NAP)_4_(2pic)_2_]_n_ may also trigger early apoptosis leading to increased HT–29 cell death which is associated with mitochondrial membrane destruction [93]. The cytotoxic activity of complex [Ni(NAP)_2_(phen)(H_2_O)] may also be attributed to apoptosis as revealed by Annexin V–FITC/PI double staining studies [62].

### 5.2. Antibacterial Activity of the Complexes

The potential antibacterial activity of the reported compounds was evaluated in vitro against diverse microorganisms. According to the literature [48,63,73,78,83,92], the antimicrobial activity of the compounds was tested against five Gram(−) bacteria (*Escherichia coli, Klebsiella pneumoniae, Proteus mirabilis, Pseudomonas aeruginosa* and *Salmonella typhi*), seven Gram(+) bacteria (*Bacillus cereus, Bacillus subtilis, Enterococcus faecalis, Listeria monocytogenes, Micrococcus luteus, Staphylococcus aureus* and *Methicillin Resistant Staphylococcus aureus*) and one yeast (*Candida albicans*). In most cases, the diameter of the inhibition zone (IZ) of the development of the microorganisms was measured for diverse concentrations of the compounds, while in fewer cases the minimum inhibitory concentration (MIC) was determined (Table S2, Supplementary Materials). In the cases of IZ measurements, a strong inhibitory effect was expected for IZ >20 mm, while IZ values in the range of 10–20 mm and <10 mm were assessed as having moderate and low antibacterial activities, respectively [83]. In the cases of MIC values, the lower the MIC values were, the better the antibacterial activity was.

In general, the antimicrobial activity of the reported Cu(II) and Zn(II) naproxen complexes was higher than free naproxen but significantly low when compared with reference compounds such as Erythromycin, Gentamycin, Vancomycin and Amphotericin B, employed in the experiments. In particular, the MIC values derived for complexes [Cu_2_(NAP)_4_(3pic)_2_] and [Cu(NAP)_2_(H_2_O)(4pic)_2_] were 256 μg/mL against *Enterococcus faecalis* and 128 μg/mL against *Candida albicans*, and the activity of the complexes was much lower than the respective reference compounds Vancomycin (MIC = 2 μg/mL) and Amphotericin B (MIC = 0.0625 μg/mL) [48]. For the six reported Zn(II)–naproxen complexes ([Zn_2_(NAP)_4_], [Zn(NAP)_2_(phen)], [Zn(NAP)_2_(neoc)], [Zn(NAP)_2_(2ampy)_2_], [Zn(NAP)_2_(Himi)_2_] and [Zn(NAP)_2_(1,2–dmimid)_2_]), the inhibition zones for treatment with 8.5 mM against diverse bacteria were much shorter (Table S2) than the reference compounds Erythromycin and Gentamycin, revealing rather low activity [63]. Similarly, the reported complexes bearing 1–naphthylacetato ligands were more active than free 1–naphthylacetic acid, but not as active as the reference compounds tested (Table S2) [73,78,83,92].

### 5.3. Antioxidant Activity of the Complexes

The reported coordination compounds of the NSAID naproxen were evaluated in vitro for their potential antioxidant activity. These studies included the scavenging of free radicals such as 1,1–diphenyl–picrylhydrazyl (DPPH), hydroxyl and 2,2′–azino–bis(3–ethylbenzothiazoline–6–sulfonic acid) (ABTS) radicals, the inhibition of the enzyme soybean lipoxygenase (LOX) and the mimicking of the enzyme superoxide dismutase (SOD).

Free radicals have unpaired electron(s) which may be transferred to neighboring molecules activating chain reactions with undesired effects in organisms such as inflammations or even cancer [101]. The antioxidant activity is often related with the donation of electrons in order to neutralize the radicals. Antioxidants are used to stop radical chain reactions either by scavenging free radicals or by inhibiting their production. The benefit of the antioxidants to organisms is the decrease in inflammations which are caused by radical-induced damages [101]. NSAIDs such as naproxen are compounds with inflammatory activity and may obviously act as radical scavengers contributing to antioxidant activity and probably to anticancer activity [102].

#### 5.3.1. Scavenging of DPPH Radicals

DPPH is a stable radical used in EPR spectroscopy [103] and as a trap to inhibit and study radical-mediated reactions in the laboratory [104]. DPPH is the most common radical used to check in vitro the radical scavenging of compounds [104]. DPPH scavengers may additionally exhibit anticancer, anti-ageing and anti-inflammatory activity and could be potential agents used for the treatment of rheumatoid arthritis and inflammation [11,105].

**Figure 23:**
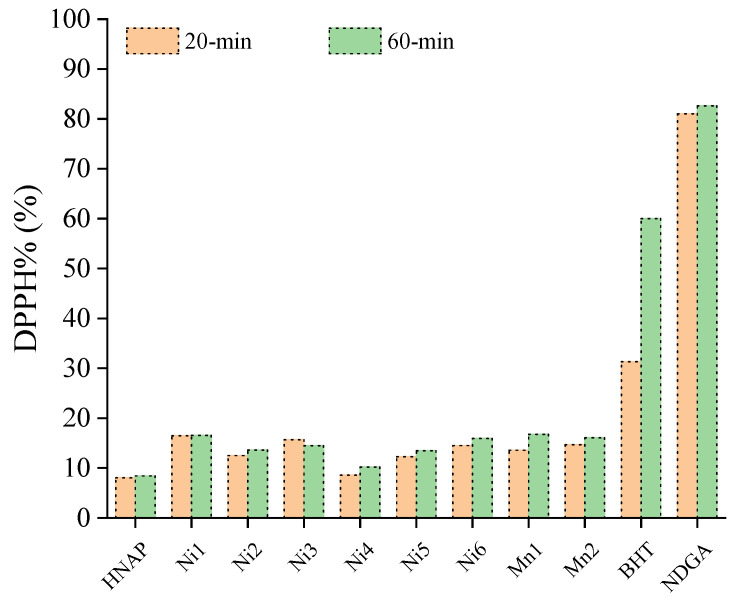
DPPH scavenging activity (DPPH%) of naproxen and its reported complexes after 20 min and 60 min incubation. (Codes of compounds: HNAP = naproxen; Ni1 = [Ni(NAP)_2_(CH_3_OH)_4_]; Ni2 = [Ni(NAP)_2_(bipy)(CH_3_OH)]; Ni3 = [Ni(NAP)_2_(phen)(H_2_O)]; Ni4 = [Ni(NAP)_2_(bipyam)]; Ni5 = [Ni(NAP)_2_(Hpko)_2_]; Ni6 = [Ni(NAP)_2_(py)_2_(H_2_O)_2_]; Mn1 = [Mn(NAP)_2_(py)_2_(H_2_O)_2_] and Mn2 = [Mn(NAP)_2_(phen)(H_2_O)].).

A series of Mn(II), Co(II), Ni(II) and Cu(II) complexes of naproxen were tested for their potency to scavenge in vitro the DPPH radicals and the results were compared with the reference compounds nordihydroguairetic acid (NDGA) and butylated hydroxytoluene (BHT). All complexes were more active DPPH scavengers than the corresponding free naproxen (Table S3). The DPPH scavenging activity of the complexes was time-independent because no significant differences in the activity were observed after 20 min and 60 min of incubation (Table S3, Figure 23) [42,43,61].

**Figure 24:**
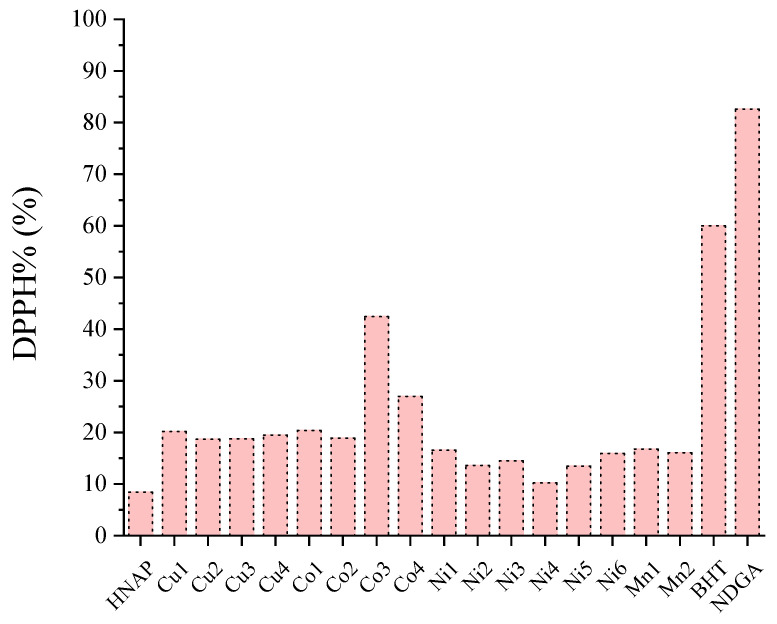
DPPH radicals’ scavenging activity (DPPH%) of naproxen and its reported complexes after 60 min treatment. (Codes of compounds: HNAP = naproxen; Cu1 = [Cu_2_(NAP)_4_(H_2_O)_2_]; Cu2 = [Cu(NAP)_2_(py)_2_(H_2_O)]; Cu3 = [Cu(NAP)_2_(phen)]·H_2_O; Cu4 = [Cu(NAP)_2_(bipy)]·H_2_O; Co1 = [Co(NAP)_2_(CH_3_OH)_4_]; Co2 = [Co(NAP)_2_(py)_2_(H_2_O)_2_]; Co3 = [Co(NAP)_2_(phen)(H_2_O)_2_]; Co4 = [Co(NAP)_2_(bipy)(H_2_O)_2_]; Ni1 = [Ni(NAP)_2_(CH_3_OH)_4_]; Ni2 = [Ni(NAP)_2_(bipy)(CH_3_OH)]; Ni3 = [Ni(NAP)_2_(phen)(H_2_O)]; Ni4 = [Ni(NAP)_2_(bipyam)]; Ni5 = [Ni(NAP)_2_(Hpko)_2_]; Ni6 = [Ni(NAP)_2_(py)_2_(H_2_O)_2_]; Mn1 = [Mn(NAP)_2_(py)_2_(H_2_O)_2_] and Mn2 = [Mn(NAP)_2_(phen)(H_2_O)].).

In general, the extent of the DPPH scavenging of the complexes was mostly dependent on the nature of the metal ion; among the reported naproxen complexes, the Cu(II) and Co(II) complexes exhibited relatively higher activities which were in all cases significantly lower than the activities of the reference compounds BHT and NDGA (Table S3, Figure 24). The best DPPH scavenger among the reported naproxen complexes was [Co(NAP)_2_(phen)(H_2_O)_2_] (DPPH% = 42.42 ± 0.13%) [42,43,61].

The co-ligands (i.e., their existence and their nature) do not seem to influence the activity of the complexes against DPPH radicals which are rather low. In conclusion, the DPPH scavenging activity of the reported metal–naproxen complexes is generally low or, in few cases, moderate.

#### 5.3.2. Scavenging of Hydroxyl Radicals

Hydroxyl radicals are very active radicals and may launch successive radical reactions leading to the production of the dangerous reactive oxygen species (ROS) which may induce damages to tissues and inflammations. Therefore, they bear a significant role in radical chemistry, although they have a rather short life. However, compounds that can initially inhibit these successive ROS reactions by scavenging hydroxyl radicals may prove to be important antioxidant factors [11,106].

**Figure 25:**
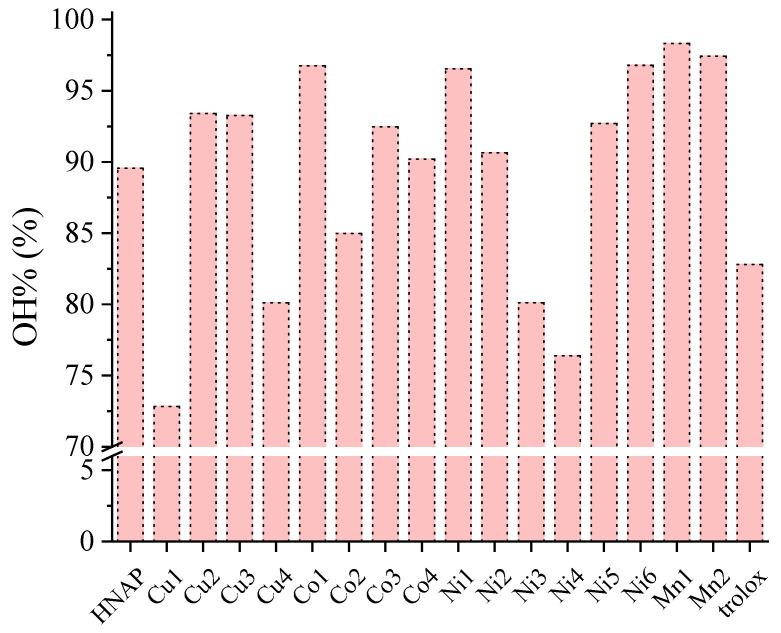
Hydroxyl radicals’ scavenging activity (OH%) of naproxen and its reported complexes. (Codes of compounds: HNAP = naproxen; Cu1 = [Cu_2_(NAP)_4_(H_2_O)_2_]; Cu2 = [Cu(NAP)_2_(py)_2_(H_2_O)]; Cu3 = [Cu(NAP)_2_(phen)]·H_2_O; Cu4 = [Cu(NAP)_2_(bipy)]·H_2_O; Co1 = [Co(NAP)_2_(CH_3_OH)_4_]; Co2 = [Co(NAP)_2_(py)_2_(H_2_O)_2_]; Co3 = [Co(NAP)_2_(phen)(H_2_O)_2_]; Co4 = [Co(NAP)_2_(bipy)(H_2_O)_2_]; Ni1 = [Ni(NAP)_2_(CH_3_OH)_4_]; Ni2 = [Ni(NAP)_2_(bipy)(CH_3_OH)]; Ni3 = [Ni(NAP)_2_(phen)(H_2_O)]; Ni4 = [Ni(NAP)_2_(bipyam)]; Ni5 = [Ni(NAP)_2_(Hpko)_2_]; Ni6 = [Ni(NAP)_2_(py)_2_(H_2_O)_2_]; Mn1 = [Mn(NAP)_2_(py)_2_(H_2_O)_2_] and Mn2 = [Mn(NAP)_2_(phen)(H_2_O)].).

The ability of Mn(II), Co(II), Ni(II) and Cu(II) complexes of naproxen to scavenge hydroxyl radicals was evaluated in vitro and compared with the reference compound 6–hydroxy–2,5,7,8–tetramethylchromane–2–carboxylic acid (trolox). Almost all of the reported complexes exhibited very high hydroxyl scavenging activity which was even higher than the reference compound trolox (Figure 25 and Table S4). Most of the reported complexes were better scavengers of hydroxyl radicals than free naproxen (Figure 25) [42,43,61].

Among the complexes, those bearing nitrogen–donor co-ligands were significantly active, with the manganese(II) complexes [Mn(NAP)_2_(py)_2_(H_2_O)_2_] (OH% = 98.31 ± 0.67%) and [Mn(NAP)_2_(phen)(H_2_O)] (OH% = 97.42 ± 0.83%) being the most potent hydroxyl scavengers among the reported compounds. In particular, in the case of Co(II) and Ni(II) complexes, the complexes not bearing nitrogen–donor co-ligands, i.e., [Co(NAP)_2_(CH_3_OH)_4_] (OH% = 96.75 ± 0.30%) and [Ni(NAP)_2_(CH_3_OH)_4_] (OH% = 96.53 ± 0.32%) were also very potent [42,43,61].

#### 5.3.3. ABTS Radical Scavenging

The cationic radical of ABTS can often react with antioxidants [107] and is used as a marker of the antioxidant capacity of foods [108] and the total antioxidant activity of radical scavengers [109]. Within context, the ability of the compounds to scavenge ABTS radicals is often determined as a supplementary experiment to hydroxyl and DPPH radicals [11] and is compared with the reference compound trolox.

**Figure 26:**
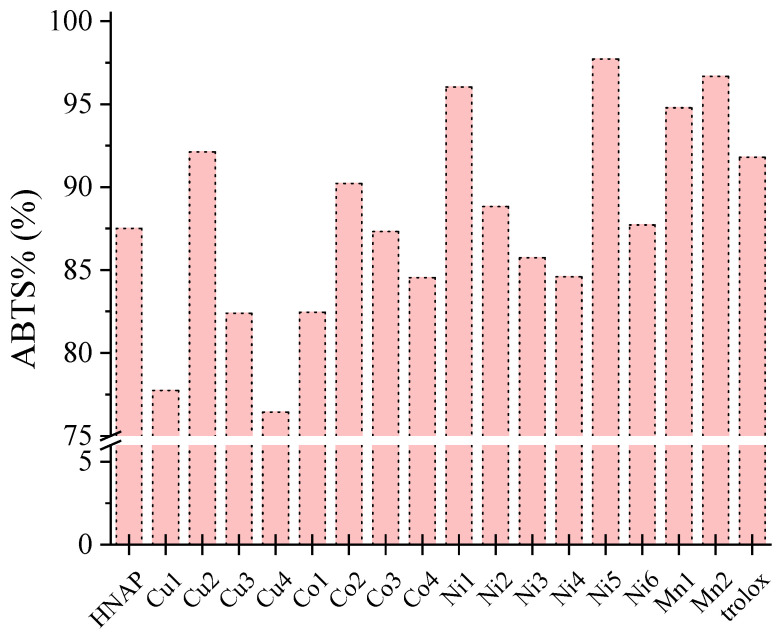
ABTS radicals’ scavenging activity (ABTS%) of naproxen and its reported complexes. (Codes of compounds: HNAP = naproxen; Cu1 = [Cu_2_(NAP)_4_(H_2_O)_2_]; Cu2 = [Cu(NAP)_2_(py)_2_(H_2_O)]; Cu3 = [Cu(NAP)_2_(phen)]·H_2_O; Cu4 = [Cu(NAP)_2_(bipy)]·H_2_O; Co1 = [Co(NAP)_2_(CH_3_OH)_4_]; Co2 = [Co(NAP)_2_(py)_2_(H_2_O)_2_]; Co3 = [Co(NAP)_2_(phen)(H_2_O)_2_]; Co4 = [Co(NAP)_2_(bipy)(H_2_O)_2_]; Ni1 = [Ni(NAP)_2_(CH_3_OH)_4_]; Ni2 = [Ni(NAP)_2_(bipy)(CH_3_OH)]; Ni3 = [Ni(NAP)_2_(phen)(H_2_O)]; Ni4 = [Ni(NAP)_2_(bipyam)]; Ni5 = [Ni(NAP)_2_(Hpko)_2_]; Ni6 = [Ni(NAP)_2_(py)_2_(H_2_O)_2_]; Mn1 = [Mn(NAP)_2_(py)_2_(H_2_O)_2_] and Mn2 = [Mn(NAP)_2_(phen)(H_2_O)].).

The in vitro ABTS scavenging ability of Mn(II), Co(II), Ni(II) and Cu(II) complexes of naproxen was reported and the results are summarized in Table S5 and in Figure 26. Many of the complexes were significantly active towards ABTS radicals and were more potent than the reference compound trolox. Among the reported complexes, the best ABTS scavengers (Figure 26) were two nickel(II) complexes, i.e., [Ni(NAP)_2_(CH_3_OH)_4_] (ABTS% = 96.03 ± 0.43%), [Ni(NAP)_2_(Hpko)_2_] (ABTS% = 97.73 ± 0.27%) and the manganese(II) complexes [Mn(NAP)_2_(py)_2_(H_2_O)_2_] (ABTS% = 94.78 ± 0.36%) and [Mn(NAP)_2_(phen)(H_2_O)] (ABTS% = 96.67 ± 0.68%) [42,43,61].

#### 5.3.4. LOX Inhibitory Activity of the Complexes

LOX is the enzyme involved in a procedure occurring via carbon-centered radicals (lipoxygenation) [110]. The inhibition of LOX activity is often related with total antioxidant activity and free radical scavenging [111]. The ability of naproxen and its Ni(II) and Ag(I) complexes [50,61] to inhibit the activity of LOX was expressed as the concentration of the compound that inhibits the activity of the enzyme by 50% (IC_50_) and was compared to the reference compounds caffeic acid and *cisplatin* (Table S6).

**Figure 27:**
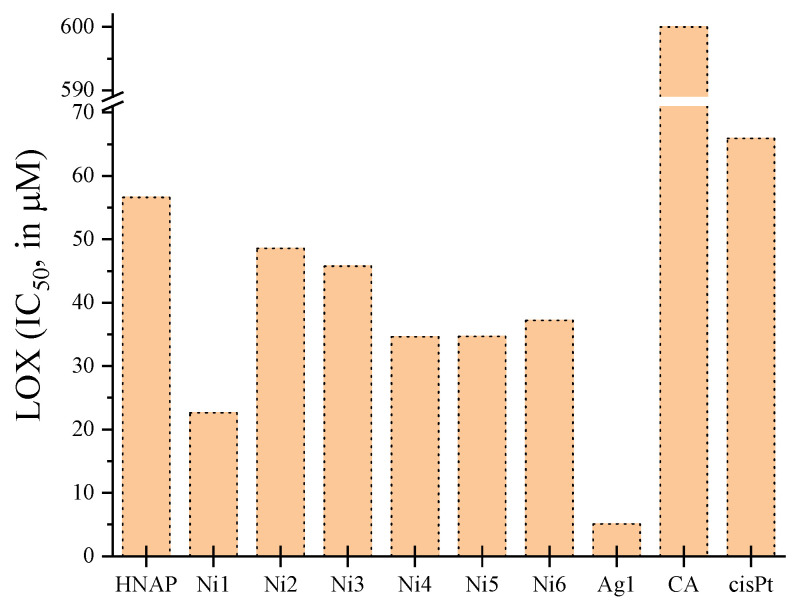
LOX inhibitory activity (IC_50_ in µM) of naproxen and its reported complexes. (Codes of compounds: HNAP = naproxen; Ni1 = [Ni(NAP)_2_(CH_3_OH)_4_]; Ni2 = [Ni(NAP)_2_(bipy)(CH_3_OH)]; Ni3 = [Ni(NAP)_2_(phen)(H_2_O)]; Ni4 = [Ni(NAP)_2_(bipyam)]; Ni5 = [Ni(NAP)_2_(Hpko)_2_]; Ni6 = [Ni(NAP)_2_(py)_2_(H_2_O)_2_]; Ag1 = [Ag(PPh_3_)_3_(NAP)](H_2_O); CA = caffeic acid and cisPt = cisplatin.).

All of the Ni(II) and Ag(I) complexes tested were better LOX-inhibitors than free naproxen and especially than the reference compounds used in each case (Figure 27). Among the complexes tested, the silver(I) complexes were the most potent compounds with [Ag(NAP)(PPh_3_)_3_](H_2_O) showing a very low IC_50_ value (=5.1 µM).

#### 5.3.5. SOD-like Activity of the Complexes

SOD is the enzyme that scavenges superoxide radicals and transforms them to oxygen and H_2_O_2_. Among the proposed mechanisms of anti-inflammatory activity of the NSAIDs, the inhibition of the generation of superoxide radicals [112] and the elimination of superoxide anions produced by nucleophiles [113] have also been considered. The SOD-like activity was reported for two Cu(II)–naproxen complexes (Table 11). The two reported complexes [Cu_2_(NAP)_4_]_n_ and [Cu(NAP)_2_(3pym)_2_]_n_ displayed significant SOD-like activity which was comparable to that of the enzyme [114].

### 5.4. Other Biological Activities

Other biological activities monitored for selected metal complexes include the catechol oxidase-like activity, the antimalarial activity and the reaction with linoleic acid.

In order to check the potential catalytic efficacy of two Cu(II)–naproxen complexes, i.e., the dinuclear [Cu_2_(NAP)_4_(3pic)_2_] and the mononuclear complex [Cu(NAP)_2_(4pic)_2_(H_2_O)], the catalytic two-electron oxidation of 3,5–di–*tert*–butylcatechol to the corresponding quinone 3,5–di–*tert*–butylquinone via the complexes was thoroughly studied [48]. The obtained results (kinetic parameters) revealed that the complexes possessed noteworthy catechol oxidase activity at room temperature. Especially, the catechol oxidase activity of the dinuclear complex [Cu_2_(NAP)_4_(3pic)_2_] was significant when it was compared with the turnover number for the catechol oxidase enzyme [48].

A series of Zn(II)–naproxen complexes were checked for their in vitro antimalarial potency [63]. More specifically, six Zn(II)–naproxen complexes were monitored for the inhibition of the in vitro formation of β–hematin and the mononuclear complexes [Zn(NAP)_2_(phen)] and [Zn(NAP)_2_(neoc)], which both bear a phenanthroline-based co-ligand, and were the only active compounds. In particular, complex [Zn(NAP)_2_(neoc)] was the most active among the tested complexes, showing an important inhibition of β–hematin formation in rather low concentrations (in the range 0.6–1 mg/mL) with efficacy > 75% which is comparable with the reference compounds Chloroquine and Amodiaquine [63].

The influence of complex [(*n*–Bu)_2_Sn(NAP)_2_] upon the peroxidation of linoleic acid was also monitored. The interaction of the complex with linoleic acid (an essential component of the cell membrane) resulted in increased amounts of hydroperoxylinoleic acid. Such a reaction could either decrease the quantity of linoleic acid in the cell membrane or even totally remove it from the membrane leading to serious damage and dysfunction of the membrane, which is important in the case of cytotoxicity studies [60].

### 5.5. Interaction of the Compounds with Biomacromolecules

Complimentary with biological activity studies, the interaction of bioactive compounds with biomacromolecules is often studied in order to evaluate the biological potency of the compounds and to check possible mechanistic pathways or biological targets. Overall, the most commonly studied biomacromolecules are DNA and albumins.

#### 5.5.1. Interaction of the Reported Complexes with DNA

DNA is among the biomolecular targets for a series of drugs including antibacterial, antiviral and anticancer agents [115], because it can regulate genetic information and may affect the replication of the cell and the protein expression. DNA is involved in diverse mechanisms of action for such drugs, e.g., drugs such as methotrexate inhibit the nucleotide synthesis, others such as doxorubicin inhibit the activity of the topoisomerase enzyme and Pt-based drugs may intervene in double-strand DNA, further blocking its replication [116]. Metal-based drugs with labile ligands (e.g., *cisplatin*) may bind covalently to DNA bases via vacant coordination sites [117], while those containing non-labile ligands remain stable and may interact noncovalently with DNA by intercalation, groove-binding and/or electrostatic interaction [117,118]. In addition, the cleavage of DNA may also be a result of its interaction with metal complexes being able to act as chemical nucleases [118] and may occur simultaneously with tight DNA binding [119,120]. With DNA being a possible target of photodynamic therapy, the changes in the DNA helix induced by irradiation in the presence of metal complexes are also interesting to study [121,122,123].

Considering the reported metal complexes with the considered ligands, many metal–naproxen complexes have been studied for their interaction with calf thymus (CT) DNA, which is the most common form of linear DNA studied. The interaction of the complexes with CT DNA was studied using diverse techniques (UV–vis spectroscopy, cyclic voltammetry, viscosity measurements and fluorescence emission spectroscopy) aiming to determine the mode of this interaction and to calculate the DNA-binding constants (K_b_). Most of the reported metal–naproxen complexes were proposed to interact with CT DNA by intercalation [42,43,49,50,56,60,61,90], i.e., via insertion of the aromatic ligands in between the DNA nucleobases which are further stabilized by the development of π–π stacking interactions.

**Figure 28:**
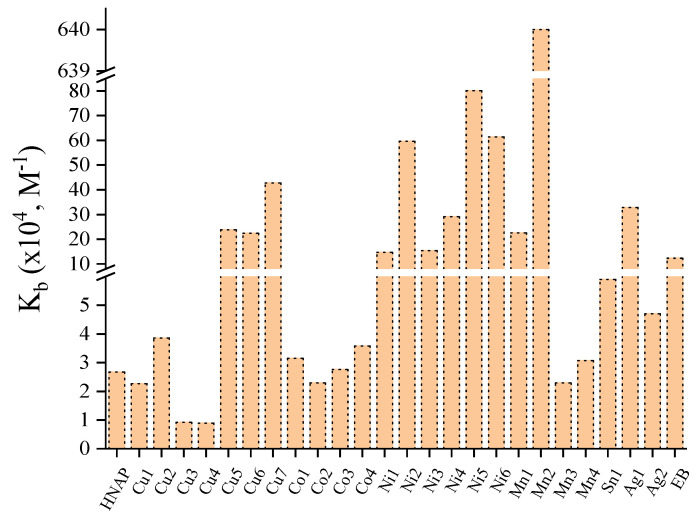
DNA–binding constants (K_b_) for naproxen and its reported complexes. (Codes of compounds: HNAP = naproxen; Cu1 = [Cu_2_(NAP)_4_(H_2_O)_2_]; Cu2 = [Cu(NAP)_2_(bipy)]; Cu3 = [Cu(NAP)_2_(phen)]; Cu4 = [Cu(NAP)_2_(py)_2_(H_2_O)]; Cu5 = [Cu(L1)(NAP)Cl]; Cu6 = [Cu(L2)(NAP)Cl]; Cu7 = [Cu(L3)(NAP)Cl]; Co1 = [Co(NAP)_2_(CH_3_OH)_4_]; Co2 = [Co(NAP)_2_(py)_2_(H_2_O)_2_]; Co3 = [Co(NAP)_2_(phen)(H_2_O)_2_]; Co4 = [Co(NAP)_2_(bipy)(H_2_O)_2_]; Ni1 = [Ni(NAP)_2_(CH_3_OH)_4_]; Ni2 = [Ni(NAP)_2_(bipy)(CH_3_OH)]; Ni3 = [Ni(NAP)_2_(phen)(H_2_O)]; Ni4 = [Ni(NAP)_2_(bipyam)]; Ni5 = [Ni(NAP)_2_(Hpko)_2_]; Ni6 = [Ni(NAP)_2_(py)_2_(H_2_O)_2_]; Mn1 = [Μn(NAP)_2_(CH_3_OH)]_n_; Mn2 = [Mn(NAP)_2_(phen)(H_2_O)]; Mn3 = [Mn(NAP)_2_(py)(H_2_O)_2_]; Mn4 = [Μn_6_(NAP)(Hsal)(shi)_6_(py)_6_]; Sn1 = [(*n*–Bu)_2_Sn(NAP–O,O′)_2_]; Ag1 = [Ag(PPh_3_)_3_(NAP)](H_2_O); Ag2 = [Ag(tptp)_2_(NAP)] and EB = ethidium bromide.).

The DNA-binding constants of the reported complexes were calculated using the Wolfe–Shimer equation [124] and their values are summarized in Table S7. The K_b_ values of the reported complexes were found in the range of 10^4^–10^6^ M^−1^, revealing a tight interaction with CT DNA. Most of the complexes were found to be tighter DNA binders than the classic DNA intercalator ethidium bromide (EB) (K_b_ = 1.23 × 10^5^ M^−1^) [125]. This fact was important for the employment of EB competitive displacement studies which were used to confirm the intercalative interaction with DNA. On the basis of K_b_ values (Table S7, Figure 28), the Ni(II) complexes were found to be the tightest DNA binders with all K_b_ values being higher than 10^5^ M^1^ [61], while the highest K_b_ value was reported for complex [Mn(NAP)_2_(phen)(H_2_O)] (K_b_ = 6.40 × 10^6^ M^−1^) [42].

In few cases, the supercoiled pBR322 plasmid DNA (pDNA) was used to study the interaction of circular DNA with complexes by gel electrophoretic experiments. More specifically, the interaction of [Cu(NAP)(L1)(Cl)], [Cu(NAP)(L2)(Cl)] and [Cu(NAP)(L3)(Cl)] with supercoiled pDNA revealed their high nuclease activity which resulted in the formation of nicked circular DNA or even linear DNA [49].

Furthermore, molecular docking calculations with DNA (PDB ID: 1BNA) were performed for a series of metal complexes in order to theoretically check the mode of DNA interaction and the DNA site where the interaction takes place. Molecular docking calculations with DNA were employed for complexes [Cu(NAP)(L1)(Cl)], [Cu(NAP)(L2)(Cl)] and [Cu(NAP)(L3)(Cl)] [49] and [(*n*–Bu)_2_Sn(NAP)_2_] [60], and the theoretical results were found to be in good agreement with the experimental findings regarding the mode of interaction and the relative affinity of the DNA interaction.

#### 5.5.2. Interaction of the Reported Complexes with Albumins

Serum albumin (SA) is a multifunctional protein in blood serum. Its abundance is related with its important biological role, since it is responsible for the acid–base equilibrium in organisms, for the maintenance of osmotic pressure in blood and for the transportation of small molecules of hormones, fatty acids, ions and drugs through the bloodstream [126,127]. Additionally, albumins also have antioxidant and anticoagulant properties which are taken into consideration for potential clinical and pharmaceutical biochemical applications [128,129]. The most commonly studied albumins are human serum albumin (HSA) and its homolog—bovine serum albumin (BSA). The structures of both albumins have been characterized by crystallography. Their three domains (I, II and III) are divided in two subdomains (A and B) [130]. Sudlow’s site 1 (or drug site I) in subdomain IIA and Sudlow’s site 2 (or drug site II) in subdomain IIIA are the most important sites to host drugs and metal ions [130]. The intense fluorescence emission bands observed for both albumins, when their solutions are excited at 295 nm, are attributed to the presence of tryptophan residues; HSA has one tryptophan residue at position 214 and BSA has two tryptophans at positions 134 and 212 [130,131].

**Figure 29:**
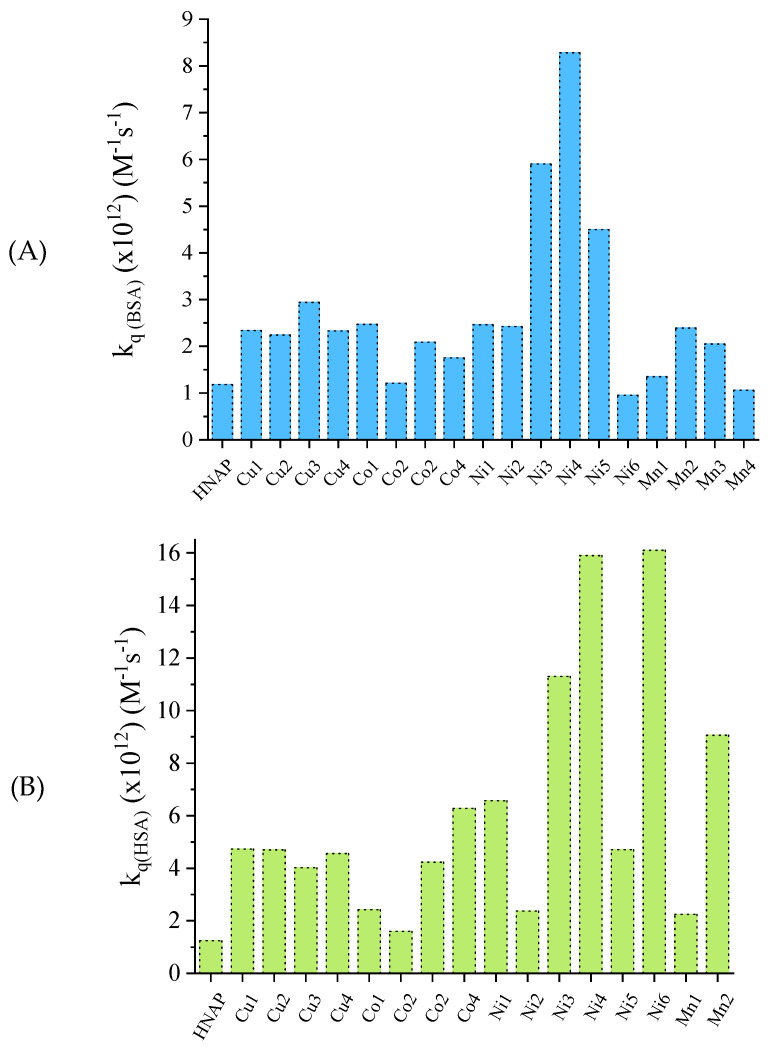
Albumin−quenching constants (k_q_) for reported metal complexes. (**A**) For BSA. (**B**) For HSA. (Codes of compounds: HNAP = naproxen; Cu1 = [Cu_2_(NAP)_4_(H_2_O)_2_]; Cu2 = [Cu(NAP)_2_(bipy)]; Cu3 = [Cu(NAP)_2_(phen)]; Cu4 = [Cu(NAP)_2_(py)_2_(H_2_O)]; Co1 = [Co(NAP)_2_(CH_3_OH)_4_]; Co2 = [Co(NAP)_2_(py)_2_(H_2_O)_2_]; Co3 = [Co(NAP)_2_(phen)(H_2_O)_2_]; Co4 = [Co(NAP)_2_(bipy)(H_2_O)_2_]; Ni1 = [Ni(NAP)_2_(CH_3_OH)_4_]; Ni2 = [Ni(NAP)_2_(bipy)(CH_3_OH)]; Ni3 = [Ni(NAP)_2_(phen)(H_2_O)]; Ni4 = [Ni(NAP)_2_(bipyam)]; Ni5 = [Ni(NAP)_2_(Hpko)_2_]; Ni6 = [Ni(NAP)_2_(py)_2_(H_2_O)_2_]; Mn1 = [Μn(NAP)_2_(CH_3_OH)]_n_; Mn2 = [Mn(NAP)_2_(py)_2_(H_2_O)_2_]; Mn3 = [Mn(NAP)_2_(phen)(H_2_O)] and Mn4 = [Μn_6_(NAP)(Hsal)(shi)_6_(py)_6_].).

The interaction of the reported metal–naproxen complexes with both albumins was monitored by fluorescence emission quenching titrations [42,43,56,61,90]. The observed quenching was an indication of the noncovalent interaction of the compounds with the albumins [132]. The SA quenching constants (k_q_) were calculated using the Stern–Volmer equation and plots [133]. The k_q_ values of the reported compounds for both albumins (Tables S8 and S9) are significantly higher than the value of 10^10^ M^−1^ s^−1^, suggesting the existence of a static quenching mechanism [134], which is confirmation of the noncovalent interaction of the compounds with the albumins. Among the reported complexes, the Ni(II)–naproxen complexes presented the highest k_q_ values for both albumins (Figure 29), i.e., [Ni(NAP)_2_(phen)(H_2_O)] and [Ni(NAP)_2_(bipyam)] for BSA and [Ni(NAP)_2_(bipyam)] and [Ni(NAP)_2_(py)_2_(H_2_O)_2_] for HSA [61].

**Figure 30:**
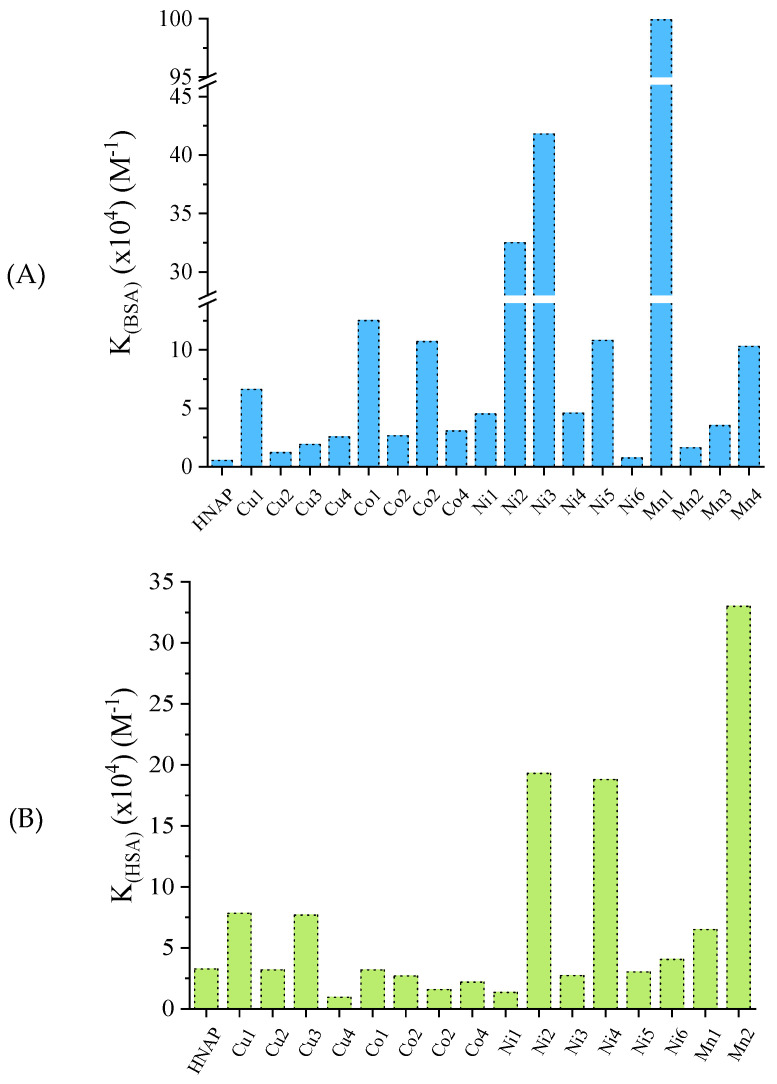
Albumin−binding constants (K) for reported metal complexes. (**A**) For BSA. (**B**) For HSA. (Codes of compounds: HNAP = naproxen; Cu1 = [Cu_2_(NAP)_4_(H_2_O)_2_]; Cu2 = [Cu(NAP)_2_(bipy)]; Cu3 = [Cu(NAP)_2_(phen)]; Cu4 = [Cu(NAP)_2_(py)_2_(H_2_O)]; Co1 = [Co(NAP)_2_(CH_3_OH)_4_]; Co2 = [Co(NAP)_2_(py)_2_(H_2_O)_2_]; Co3 = [Co(NAP)_2_(phen)(H_2_O)_2_]; Co4 = [Co(NAP)_2_(bipy)(H_2_O)_2_]; Ni1 = [Ni(NAP)_2_(CH_3_OH)_4_]; Ni2 = [Ni(NAP)_2_(bipy)(CH_3_OH)]; Ni3 = [Ni(NAP)_2_(phen)(H_2_O)]; Ni4 = [Ni(NAP)_2_(bipyam)]; Ni5 = [Ni(NAP)_2_(Hpko)_2_]; Ni6 = [Ni(NAP)_2_(py)_2_(H_2_O)_2_]; Mn1 = [Μn(NAP)_2_(CH_3_OH)]_n_; Mn2 = [Mn(NAP)_2_(py)_2_(H_2_O)_2_]; Mn3 = [Mn(NAP)_2_(phen)(H_2_O)] and Mn4 = [Μn_6_(NAP)(Hsal)(shi)_6_(py)_6_].).

The SA-binding constants (K) of the reported complexes were determined using the Scatchard equation and respective plots [133]. The K values of the reported compounds for both albumins (Tables S8 and S9) are higher than that of free naproxen and are of the 10^4^–10^6^ M^−1^ magnitude [42,43,56,61,90]. These values are indicative of tight binding to both albumins which may be considered reversible, when compared with the highest noncovalent binding constant of value 10^15^ M^−1^ [135]. This reversible binding may allow the release of the bioactive compounds when approaching their biological targets. Among the reported complexes, the manganese(II)–naproxen complexes presented the highest K values for both albumins (Figure 30), i.e., [Μn(NAP)_2_(CH_3_OH)]_n_ for BSA [90] and [Mn(NAP)_2_(py)_2_(H_2_O)_2_] for HSA [42].

## 6. Conclusions and Perspectives

As shown in the discussion, the presence of naproxen, 1–naphthylacetato, 2–naphthylacetato and 1–pyreneacetato ligands has generated a lot of interesting complexes concerning their structures and their photochemical and in vitro biological properties. The characterization of the complexes was achieved using X-ray crystallography and diverse physicochemical and spectroscopic techniques.

Among the metal ions participating in these complexes, most of them belong either to the first row transition metals (Ti(IV), Mn(II/III), Fe(III), Co(II), Ni(II), Cu(II) and Zn(II)) or to lanthanides (Pr(III), Nd(III), Sm(III), Eu(III), Gd(III), Tb(III), Dy(III), Ho(III) and Yb(III)). Of course, there are some metal ions from second and third row transition metals (Y(III), Ru(II/III), Ag(I), Cd(II) and Au(I)) as well as from the s-block (Mg(II)) and p-block (Sn(IV)) of the periodic table. Indeed, the number of so-far-reported metal ions participating in such complexes is significant, leaving, however, opportunities and perspectives for the remaining transition metal ions that could also be taken into consideration for the formation of novel coordination compounds. The role and number of the co-ligands are important, since they contribute to the stabilization of the complexes and may offer, in most cases, synergism in the reported biological properties. Most of the co-ligands are oxygen–donors or heterocyclic nitrogen–donors.

Considering the nuclearity of the reported coordination compounds, most of them are mononuclear and dinuclear complexes. A few examples of trinuclear, tetranuclear and hexanuclear complexes have also been reported, where the ligands under study have mainly played an important bridging role. The reported polymeric complexes could be categorized to those owing their polymeric structure to bridges formed by the ligands under study and to compounds where naphthylacetato and naproxen ligands are terminal and do not contribute to the polymeric nature of the compounds.

The naproxen, 1–naphthylacetato, 2–naphthylacetato and 1–pyreneacetato ligands have been found in diverse coordination modes, either in one discrete mode or in a combination of two or even three different fashions. Monodentate and bidentate chelating modes and their combination were observed in the cases of mononuclear complexes. In the case of dinuclear, polynuclear and polymeric complexes, the concerned ligands were found in the bidentate (μ–O,O′) bridging mode either alone or in combination with the tridentate (μ–O,O,O′) bridging or bidentate chelating mode, in bidentate (μ–O,O) bridging fashion in combination with monodentate coordination or in the tridentate (μ–O,O,O′) bridging mode in combination with bidentate chelating binding. In the polynuclear complexes, the co-existence of other bridges such as µ–O or µ–*shi*–N,O contributed further to the formation of the polynuclear metallocyclic structures.

Among the physicochemical and spectroscopic techniques used, IR spectroscopy provided information regarding the coordination mode of the carboxylato ligands and the co-ligands. NMR, EPR and Mössbauer spectroscopies were also employed in cases where the nature of the metal ions allowed for their application. The photochemical properties of the complexes were focused on the study of the UV–vis spectra and the photoluminescence properties. The role and the choice of the naphthalene ring were crucial for these properties since the fluorescence properties of the transition metal complexes were assigned to intraligand π–π* transitions, while, for the lanthanide(III) complexes, they were attributed to f–f transitions. Thermogravimetric studies, magnetic measurements and cyclic voltammetry studies revealed their thermal, magnetic and electrochemical behavior, respectively.

The potential biological properties of selected reported complexes are significant. Selected complexes showed in vitro noteworthy cytotoxic potency towards diverse cancer cell lines which, in most cases, were even better than the reference compounds employed. The reported studies concerning the possible mechanism of cytotoxicity presented by selected complexes revealed their ability to induce apoptosis on studied cancer cell lines. The in vitro antibacterial activity of the selected complexes was evaluated against diverse microorganisms. The in vitro antioxidant activity of naproxen complexes was examined regarding free radical scavenging, the inhibition of the LOX enzyme and the SOD-like activity of selected compounds, and revealed their antioxidant potency. In addition, there are unique reports concerning the catalytic oxidation of 3,5–di–*tert*–butylcatechol to 3,5–di–*tert*–butylquinone, the in vitro antimalarial potency of a series of Zn(II)–naproxen complexes and the influence of a Sn(IV)–naproxen complex upon the peroxidation of linoleic acid. The interaction and affinity of selected naproxen complexes with CT DNA and serum albumins were studied in the context of their influence on biomacromolecules. All of these data have revealed the potential biological relevance of the so-far-reported coordination compounds of naproxen and 1– or 2–naphthylacetato ligands and may open novel pathways regarding biological activities and mechanisms.

In conclusion, naproxen, 1–naphthylacetic acid, 2–naphthylacetic and 1–pyreneacetic acid may serve as interesting ligands in their coordination compounds as their binding to metal ions has resulted in diverse interesting structures bearing noteworthy spectroscopic, magnetic and biological properties. The research concerning these ligands and possible derivatives may be focused on the use of unexplored metal ions for novel complexes, the formation of novel organic derivatives that may serve as ligands in future complexes by introducing modification in the naphthalene or pyrene rings and the in-depth investigation of the reported properties in order to explore potential mechanistic pathways and to reveal differentiated activities using more elaborate experimental and theoretical studies.

## Figures and Tables

**Table 1:** Mononuclear complexes of 1–naphthylacetato, 2–naphthylacetato and naproxen ligands. Coordination mode of the ligands, CCDC name of the complex, coordination sphere and geometry of the metal ions.

Complex	CCDC Name	Coordination Sphere of Ion M	Geometry of Ion M ^a^	Reference
**I: Monodentate coordination of the ligands**				
[Mg(NAP–O)_2_(H_2_O)_4_]	ANOMIA	MO_6_	Oh ^a^	[41]
[Mn(NAP–O)_2_(py)_2_(H_2_O)_2_] ^b^	GIKLEU	MN_2_O_4_	Oh	[42]
[Co(NAP–O)_2_(py)_2_(H_2_O)_2_]	KATSUV	MN_2_O_4_	Oh	[43]
[Co(NAP–O)_2_(cyclam)]	TEMKUU	MN_4_O_2_	Oh	[44]
[Cu(N1A–O)_2_(Hdmpz)_2_]	HOGTII	MN_2_O_2_	Spl	[45]
[Cu(NAP–O)_2_(H_2_O)_3_]·H_2_O	ZASZAW	MO_5_	Spy	[46]
[Cu(N1A–O)(EDA)_2_](ClO_4_)	TUFTUL	MN_4_O	Spy	[47]
[Cu(NAP–O)_2_(H_2_O)(4pic)_2_]	RANHOG	MN_2_O_3_	Spy	[48]
[Cu(NAP–O)(L1)Cl]	RUHJIP	MN_3_OCl	Spy	[49]
[Cu(NAP–O)(L2)Cl]	BUSYEV	MN_3_OCl	Spy	[49]
[Zn(NAP–O)_2_(H_2_O)_2_]·H_2_O	ZASZEA	MO_4_	Td	[46]
[Ag(NAP–O)(PPh_3_)_3_]·H_2_O	QIZWED	MOP_3_	Td	[50]
[Au(NAP–O)(PPh_3_)]	ZICVIU	MOP	L	[51]
[Gd(N1A–O)_2_(phen)_2_(H_2_O)_2_]·[Eu(N1A–O)_2_(phen)_2_(H_2_O)_2_]·(N1A)_2_·2H_2_O	HOFMEW	Co-crystallized/MN_4_O_4_	Dh	[52]
**II: Bidentate chelating coordination of the ligand**				
[Mn(N1A–O,O′)_2_(Hbzmd)_2_]·H_2_O	LANDEL	MN_2_O_4_	Oh	[53]
[Ni(N1A–O,O′)_2_(Hbzmd–N3)_2_]·H_2_O	YAVLIS	MN_2_O_4_	Oh	[54]
[Cu(NAP–O,O′)_2_(4,7–diPhphen)]	EYIKAA	MN_2_O_4_	Oh	[55]
[Cu(NAP–O,O′)_2_(bipy)]·H_2_O	LEBVIZ	MN_2_O_4_	Oh	[56]
[Cu(NAP–O,O′)_2_(phen)]·H_2_O	LEBVOF	MN_2_O_4_	Oh	[56]
[Zn(N1A–O,O′)_2_(phen)]	NEQLIF, NEQLIF01	MN_2_O_4_	Oh	[57,58]
[Ag(NAP–O,O′)(tptp)_2_]	QIZWAZ	MO_2_P_2_	Td	[50]
[Cd(NAP–O,O′)_2_(H_2_O)_2_]·H_2_O	ZASZUQ	MO_6_	Oh	[46]
[(*n*–Bu)_2_Sn(NAP–O,O′)_2_]	XISCAF, XISCAF01	MC_2_O_4_	Oh	[59,60]
**III: Monodentate + bidentate chelating coordination of the ligands**				
[Mn(NAP–O)(NAP–O,O′)(phen)(H_2_O)]	GIHJEP	MN_2_O_4_	Oh	[42]
[Ni(NAP–O)(NAP–O,O′)(bipy)(H_2_O)]	YAQJEI	MN_2_O_4_	Oh	[61]
[Ni(NAP–O)(NAP–O,O′)(phen)(H_2_O)]	YAQJAE, GADYET	MN_2_O_4_	Oh	[61,62]
[Zn(NAP–O,O′)_2_(neoc)]	NORBED	MN_2_O_3_	Spy–TB	[63]
[Zn(N1A–O)(N1A–O,O′)(5,5′–Me_2_–bipy)]	UQUSAB	MN_2_O_3_	Spy–TB	[64]

^a^ Dh = dodecahedral; L = linear; Oh = octahedral; Spl = square planar; Spy = square pyramidal; TB = trigonal bipyramidal; Td = tetrahedral; ^b^ 4pic = 4–picoline; 4,7–diPhphen = 4,7–diphenyl–1,10–phenanthroline; 5,5′–Me_2_–bipy = 5,5′–dimethyl–2,2′–bipyridine; bipy = 2,2′–bipyridine; cyclam = 1,4,8,11–tetraazacyclotetradecane; EDA = *N,N*–dimethylethane–1,2–diamine; Hbzmd = 1H–benzimidazole; Hdmpz = 3,5–dimethylpyrazole; L1 = 4′–(4–tolyl)–2,2′:6′,2″–terpyridine; L2 = 4′–(furan–2–yl)–2,2′:6′,2″–terpyridine; *n*–Bu = *n*–butyl; neoc = neocuproine = 2,9–dimethyl–1,10–phenanthroline; phen = 1,10–phenanthroline; py = pyridine; tptp = tri(p–tolyl)phosphine.

**Table 2:** Dinuclear complexes of 1–naphthylacetato, 2–naphthylacetato, naproxen and 1–pyreneacetato ligands. Coordination mode of the ligands, CCDC name of the complex, oxidation state and coordination sphere of the metal ions.

Complex	CCDC Name	Metal Ions	Coord.Sphere	Reference
**I: Bidentate bridging (μ–O,O′)**				
[Fe_2_(µ_2_–O)(µ_2_–N2A–O,O′)(tren)_2_](BPh_4_)(NO_3_)_2_	MAXXUF	Fe(III)_2_	MN_3_O_2_	[65]
[Fe_2_(µ_2_–O)(µ_2_–N2A–O,O′)(TPA)_2_](ClO_4_)_3_	MAXYAM	Fe(III)_2_	MN_3_O_2_	[65]
[Fe_2_(µ_2_–O)(µ_2_–N2A–O,O′)_2_(Tp)_2_]	MAXYEQ	Fe(III)_2_	MN_3_O_3_	[65]
[Fe_2_(µ_2_–O)(µ_2_–N2A–O,O′)_2_(TACN–Me_3_)_2_](PF_6_)_2_	MUTKIV	Fe(III)_2_	MN_3_O_3_	[66]
[Cu_2_(µ_2_–N2A–O,O′)_4_(DMSO)_2_]·2(HN2A)·2DMSO	IXAFOC	Cu(II)_2_	MO_5_	[67]
[Cu_2_(µ_2_–N2A–O,O′)_4_(DMF)_2_]	LANDUB	Cu(II)_2_	MO_5_	[68]
[Cu_2_(μ_2_–NAP–O,O′)_4_(3pic)_2_]	RANHIA	Cu(II)_2_	MNO_4_	[48]
[Cu_2_(μ_2_–NAP–O,O′)_4_(caf)_2_]	XIRCOQ	Cu(II)_2_	MNO_4_	[69]
[Zn_2_(µ_2_–OH)(µ_2_–N2A–O,O′)_2_(TACN–Me_3_)_2_](ClO_4_)	MUTKOB	Zn(II)_2_	MN_3_O_3_	[66]
[Zn_2_(µ_2_–N2A–O,O′)_4_(phdat)_2_]	TEWNEQ	Zn(II)_2_	MNO_4_	[70]
[Ru_2_(µ_2_–N2A–O,O′)_4_(H_2_O)_2_](PF_6_)·THF	YISFIR	Ru(II)/Ru(III)	MO_5_	[71]
[Ru_2_(µ_2_–N1A–O,O′)_4_(THF)_2_](PF_6_)·THF	YISFUD	Ru(II)/Ru(III)	MO_5_	[71]
K[Ru_2_(µ_2_–N2A–O,O′)_2_(dhpta)]	KEJKOA	Ru(III)_2_	MNO_5_	[72]
[Ru_2_(µ_2_–PYA–O,O′)_2_(CO)_4_(PPh_3_)_2_]	FIKWAZ	Ru(I)_2_	MC_2_PO_2_	[35]
**II: Tridentate bridging (μ–O,O,O′) + bidentate bridging (μ–O,O′) + bidentate chelating (** **κ–O,O′** **)**				
[Y_2_(µ_2_–N1A–O,O,O′)_2_(µ_2_–N1A–O,O′)_2_(κ–N1A–O,O′)_2_(phen)_2_]·DMF	LULCEB	Y(III)_2_	MN_2_O_7_	[73]
[Pr_2_(µ_2_–N1A–O,O,O′)_2_(µ_2_–N1A–O,O′)_2_(κ–N1A–O,O′)_2_(phen)_2_]·DMF	SILLOP	Pr(III)_2_	MN_2_O_7_	[73,74]
[Nd_2_(µ_2_–N1A–O,O,O′)_2_(µ_2_–N1A–O,O′)_2_(κ–N1A–O,O′)_2_(phen)_2_]	JOSJUZ	Nd(III)_2_	MN_2_O_7_	[75]
[Sm_2_(µ_2_–N1A–O,O,O′)_2_(µ_2_–N1A–O,O′)_2_(κ–N1A–O,O′)_2_(phen)_2_]·DMF	TIPBEA	Sm(III)_2_	MN_2_O_7_	[73,76]
[Eu_2_(µ_2_–N1A–O,O,O′)_2_(µ_2_–N1A–O,O′)_2_(κ–N1A–O,O′)_2_(phen)_2_]·DMF	SILLIJ	Eu(III)_2_	MN_2_O_7_	[73,77]
[GdTb(µ_2_–N1A–O,O,O′)_2_(µ_2_–N1A–O,O′)_2_(κ–N1A–O,O′)_2_(phen)_2_]	YUNFUJ	Gd(III)Tb(III)	MN_2_O_7_	[78]
[Gd_2_(µ_2_–N1A–O,O,O′)_2_(µ_2_–N1A–O,O′)_2_(κ–N1A–O,O′)_2_(phen)_2_]·DMF	SILMIK	Gd(III)_2_	MN_2_O_7_	[73,79]
[Tb_2_(µ_2_–N1A–O,O,O′)_2_(µ_2_–N1A–O,O′)_2_(κ–N1A–O,O′)_2_(phen)_2_]·DMF	SILMEG	Tb(III)_2_	MN_2_O_7_	[73,78,80]
[Yb_2_(µ_2_–N1A–O,O,O′)_2_(µ_2_–N1A–O,O′)_2_(κ–N1A–O,O′)_2_(phen)_2_]·DMF	WINCIG	Yb(III)_2_	MN_2_O_7_	[73,81]
**III: Bidentate bridging (μ–O,O′) + bidentate chelating (** **κ–O,O′)**				
[Gd_2_(µ_2_–NAP–O,O′)_4_(NAP–O,O′)_2_(phen)_2_]	ZURGAW	Gd(III)_2_	MN_2_O_6_	[82]
[Dy_2_(µ_2_–NAP–O,O′)_4_(NAP–O,O′)_2_(phen)_2_]	ZURFUP	Dy(III)_2_	MN_2_O_6_	[82]
**IV: Bidentate bridging (μ–O,O) + monodentate (** **κ–O)**				
[Cu_2_(µ_2_–N1A–O,O)_2_(N1A–O)_2_(Himi)_4_]	WURVIQ	Cu(II)_2_	MN_2_O_3_	[83]
**V: Tridentate bridging (μ–O,O,O′) + bidentate chelating (** **κ–O,O′)**				
[Cd_2_(µ_2_–N1A–O,O,O′)_2_(κ–N1A–O,O′)_2_(phen)_2_]	ZUSYUJ	Cd(II)_2_	MN_2_O_4_	[84]
**VI: Monodentate binding, bridging from other co-ligand**				
[Zn_2_(μ–dmpz)_2_(Hdmpz)_2_(N1A–O)_2_]	TOGQEN	Zn(II)	MN_3_O	[85]

3pic = 3–picoline; caf = caffeine; DMF = N,N–dimethylformamide; DMSO = dimethylsulfoxide; Hdmpz = 3,5–dimethylpyrazole; H_5_dhpta = 1,3–diamino–2–hydroxypropane–*N,N,N*′*,N*′–tetraacetic acid; Himi = imidazole; phdat = 2,4–diamine–6–phenyl–1,3,5–triazine; phen = 1,10–phenanthroline; TACN–Me_3_ = 1,4,7–trimethyl–1,4,7–triazacyclononane; THF = tetrahydrofuran; Tp = hydrotrispyrazolylborate; TPA = tris(2–pyridyl)amine and tren = tris(2–aminoethyl)amine.

**Table 3:** Polynuclear complexes of 1–naphthylacetato, 2–naphthylacetato and naproxen ligands. Coordination mode of the ligands and CCDC name of the complex.

Complex	CCDC Name	Reference
[Mn_3_(µ_3_–O)(µ_2_–N1A–O,O′)_6_(py)_3_]	OFILAS	[86]
[Cu_4_(µ_2_–N1A–O,O′)_6_(µ_2_–N1A–O,O,O′)_2_(CH_3_CN)_2_]	QIJYOZ	[87]
[Cd_4_(µ_2_–N1A–O,O′)_4_(µ_2_–N1A–O,O,O′)_2_(N1A–O)_2_(µ_2_–H_2_O)_2_(bipy)_2_]	FUVZED	[84]
[(Me_3_Sn)_4_(µ_2_–NAP–O,O′)_4_]	COFTOJ	[59]
{[(*n*–Bu)_2_Sn]_2_(µ_2_–N1A–O,O′)(µ_2_–N1A–O,O)(µ_3_–O)}_2_	OMOLIN	[88]
[Ti_6_(μ_3_–O)_2_(μ_2_–O)_2_(μ_3_–phenyl–phosphonato)_2_(μ_2_–isopropoxo)_4_(isopropoxo)_6_(μ_2_–N2A–O,O′)_2_]	EHOGOZ	[89]
[Μn_6_(µ_3_–NAP–O,O,O′)(µ_2_–Hsal–O,O′)(µ_2_–shi–N,O)_5_(py)_6_]	NIHVEI	[90]

bipy = 2,2′–bipyridine; H_2_sal = salicylic acid; H_3_shi = salicylhydroxamic acid and py = pyridine.

**Table 4:** Polymeric complexes of 1–naphthylacetato and naproxen ligands. Coordination mode of the ligands, CCDC name of the complex and polymerization linkers.

Complex	CCDC Name	Polymerized *via*	Reference
**Part I: As bridging ligands**			
[Mg(µ_2_–NAP–O,O)(µ_2_–NAP–O,O′)(µ_2_–H_2_O)]_n_	ANOMEW	µ_2_–O,O, µ_2_–O,O′, µ_2_–H_2_O	[41]
[Mn(µ_2_–NAP–O,O′)_2_(CH_3_OH)]_n_	NIHVOS	µ_2_–O,O′	[90]
[Co(µ_2_–N1A–O,O′)_2_(H_2_O)_2_]_n_	MEQHAS	µ_2_–O,O′	[91,92]
[Cd(NAP–O,O′)(µ_2_–NAP–O,O,O′)(H_2_O)]_n_	ZASZIE	µ_2_–O,O,O′	[46]
[(Ph_3_Sn)(µ_2_–NAP–O,O′)]_n_	COFTID	µ_2_–O,O′	[59]
[(*n*–Bu)_3_Sn(µ_2_–N1A–O,O,O′)]_n_	OMOLOT	µ_2_–O,O,O′	[88]
[Ag_4_(µ_2_–NAP–O,O′)_2_(µ_3_–NAP–O,O,O′)_2_(2pic)_2_]_n_	CAVHOA	µ_2_–O,O,O′	[93]
**Part II: As coordinated ligands, other bridges**			
[Zn(NAP–O)_2_(µ–3U)]_n_	OMALIA	3U	[94]
[Zn(NAP–O)_2_(µ–4U)]_n_	OMALEW	4U	[94]
[Cd_3_(µ_2_–N1A–O,O′)_2_(µ_2_–N1A–O,O,O′)_2_(µ_2_–4,4′–bipy)_2_(κ–N1A–O,O′)_2_]_n_	FUVZIH	4,4′–bipy	[84]
[Cd(µ_3_–pyr3O)(N1A–O,O′)(H_2_O)]_n_,	MIRSAI	pyr3O	[84]
[Ag(N1A–O)(µ–bpp–N,N′)]_n_	VIGDIA	bpp	[95]

2pic = 2–picoline; 3U = 1,3–dipyridin–3–ylurea; 4U = 1,3–dipyridin–4–ylurea; 4,4′–bipy = 4,4′–bipyridine; bpp = 1,3–bis(4–pyridyl)propane and pyr3OH = pyridin–3–ol.

**Table 5:** Luminescence data for reported complexes.

Complex	*λ*_excitation_ (nm)	*λ_max,_*_emission_ (nm) (Transition)	Reference
[Eu_2_(N1A)_6_(phen)_2_]·2DMF	322	581 (^5^D_0_→^7^F_0_), 593 (^5^D_0_→F_1_), 618 (^5^D_0_→^7^F_2_), 651 (^5^D_0_→^7^F_3_), 694 (^5^D_0_→^7^F_4_)	[73]
[Sm_2_(N1A)_6_(phen)_2_]·2DMF	335	566 (^4^G_5/2_→^6^H_5*/*2_), 594 (^4^G_5/2_→^6^H_7*/*2_), 617 (^4^G_5/2_→^6^H_7*/*2_), 648 (^4^G_5/2_→^6^H_9*/*2_), 680 (^4^G_5/2_→^6^H_11*/*2_)	[73]
[Tb_2_(N1A)_6_(phen)_2_]·2DMF	345	545 (^5^D_4_→^7^F_5_), 594 (^5^D_4_→^7^F_4_), 617 (^5^D_4_→^7^F_3_), 675 (^5^D_4_→^7^F_2_)	[73]
[Eu(N1A)_2_(phen)_2_(H_2_O)_2_](N1A)_2_·2H_2_O	332	580 (^5^D_0_→^7^F_0_), 592 (^5^D_0_→^7^F_1_), 617 (^5^D_0_→^7^F_2_), 674 (^5^D_0_→^7^F_3_), 697 (^5^D_0_→^7^F_4_)	[52]
[Gd_2_(N1A)_6_(phen)_2_]	351	490 (^5^D_4_→^7^F_6_), 593 (^5^D_4_→^7^F_4_), 615 (^5^D_4_→^7^F_3_), 645 (^5^D_4_→^7^F_2_)	[78]
[Tb_2_(N1A)_6_(phen)_2_].DMF	359	490 (^5^D_4_→^7^F_5_), 594 (^5^D_4_→^7^F_4_), 615 (^5^D_4_→^7^F_3_)	[78]

DMF = N,N-dimethylformamide; phen = 1,10-phenanthroline.

**Table 6:** Data regarding the thermal behavior and stability of reported complexes.

Compounds	Stable Until (°C)	Steps	Temperature per Step	Reference
[Cu_2_(NAP)_4_(3pic)_2_]	161	two	I: 130–177 II: 177–455	[48]
[Cu(NAP)_2_(H_2_O)(4pic)_2_]	122	two	I: 30–173 II: 173–461	[48]
[Cu_4_(N1A)_8_(CH_3_CN)_2_]	175.7	three	I: 175.7–185.2 II: 252.8–266.9 III: 493.8–587.5	[87]
[Cu(N1A)(EDA)_2_](ClO_4_)	Not given	two	I: 162–355 II: 355–616	[47]
[Cu(N1A)_2_(Hdmpz)_2_]	180	two	I: 188.4–270.4 II: 290.7–433.1	[45]
[Zn_2_(dmpz)_2_(Hdmpz)_2_(N1A)_2_]	180	three	I: 188.4–282.8 II: 285.4–422.6 III: 437.7–606.4	[85]
[Zn_2_(N2A)_4_(phdat)_2_]	208	three	I + II + III: 208–586	[70]
[Ag_4_(NAP)_4_(2pic)_2_]_n_	Not given	two	I: 30–204 II: 204–557	[93]
[Cd_2_(N1A)_4_(phen)_2_]	267	two	I + II: 267–483	[84]
[Cd_4_(N1A)_8_(bipy)_2_(H_2_O)_2_]	130	two	I: 130–190 II: 230–452	[84]
[Cd_3_(N1A)_6_(4,4′–bipy)_2_]_n_	294	one	294–449	[84]

2pic = 2–picoline; 3pic = 3–picoline; 4pic = 4–picoline; 4,4′–bipy = 4,4′–bipyridine; bipy = 2,2′–bipyridine; EDA = *N,N*–dimethylethane–1,2–diamine; Hdmpz = 3,5–dimethylpyrazole; phdat = 2,4–diamine–6–phenyl–1,3,5–triazine and phen = 1,10–phenanthroline.

**Table 7:** Magnetic parameters derived from the fitting to temperature-dependent molar susceptibility of reported complexes.

Complex	*J* (cm^−1^)	g	Reference
[Mn_3_(µ_3_–O)(µ_2_–N1A–O,O′)_6_(py)_3_]	−7.5 ^a^, −5.0 ^b^	2.06	[86]
[Mn_3_(µ_3_–O)(µ_2_–N2A–O,O′)_6_(py)_3_]	−7.0 ^a^, −4.9 ^b^	2.06	[86]
[Fe_2_(µ_2_–O)(µ_2_–N2A–O,O′)_2_(TACN–Me_3_)_2_](PF_6_)_2_	−105		[66]
[Fe_2_(µ_2_–O)(µ_2_–N2A–O,O′)(tren)_2_](BPh_4_)(NO_3_)_2_ [Fe_2_(µ_2_–O)(µ_2_–N2A–O,O′)(TPA)_2_](ClO_4_)_3_ [Fe_2_(µ_2_–O)(µ_2_–N2A–O,O′)_2_(Tp)_2_]	−130 ± 10		[66]
[Cu_4_(N1A)_8_(CH_3_CN)]	2*J*_1_ = −295 ^c^, 2*J*_2_ = −38 ^c^	2.28	[87]
K[Ru_2_(µ_2_–N1A–O,O′)_2_(dhpta)]	−581	2.1	[72]
K[Ru_2_(µ_2_–N2A–O,O′)_2_(dhpta)]	−378	2.1	[72]

H_5_dhpta = 1,3–diamino–2–hydroxypropane–N,N,N′,N′–tetraacetic acid; py = pyridine; TACN–Me_3_ = 1,4,7–trimethyl–1,4,7–triazacyclononane; Tp^–^ = hydrotrispyrazolylborate; TPA = tris(2–pyridyl)amine and tren = tris(2–aminoethyl)amine. ^a^ Value of the Mn(II)–Mn(III) interaction. ^b^ Value of the Mn(III)–Mn(III) interaction. ^c^ Value for different Cu(II)–Cu(II) interactions.

**Table 8:** Redox potentials (in V) and process for complexes bearing 1–naphthylacetato or 2–naphthylacetato ligands.

Complex		Naphthalene Moiety		Metal-Centered		Reference
	Solvent	Reduction (E_pc_)	Oxidation (E_pa_)	Reduction (E_pc_)	Oxidation (E_pa_)	
[Fe_2_O(N2A)(tren)_2_](BPh_4_)(NO_3_)_2_	CH_3_CN	−1.78	+1.46	−0.60 ^a^		[65]
[Fe_2_O(N2A)(TPA)_2_](ClO_4_)_3_	CH_3_CN	−2.20, −2.40	+1.50	−1.20 ^a^	+0.83 ^b^	[65]
[Fe_2_O(N2A)_2_(Tp)_2_]	CH_3_CN	−2.05	+1.60	−1.07 ^a^	+1.30 ^b^	[65]
[Fe_2_O(N2A)_2_(TACN–Me_3_)_2_](PF_6_)_2_	CH_3_CN	−1.9	+1.65	−0.75 ^a,c^		[66]
[Mn_3_O(N1A)_6_(py)_3_]	CH_2_Cl_2_		+1.05	−0.70 ^c,e^	0.02 ^c,d^ −0.61	[86]
[Mn_3_O(N2A)_6_(py)_3_]	CH_2_Cl_2_		+1.03	−0.61 ^c,e^	0.09 ^c,d^ −0.57 ^c,d^	[86]
K[Ru_2_(N1A)_2_(dhpta)]	CH_3_CN DMF		+1.29	−1.05 ^f^, −1.36 ^g^ −1.27 ^f^, −1.73 ^g^	0.63 ^c,h^ 0.53 ^c,h^	[72]
K[Ru_2_(N2A)_2_(dhpta)]	CH_3_CN DMF		+1.30	−1.05 ^f^, −1.34 ^g^ −1.28 ^f^, −1.71 ^g^	0.64 ^c,f^ 0.54 ^c,f^	[72]

H_5_dhpta = 1,3–diamino–2–hydroxypropane–N,N,N′,N′–tetraacetic acid; py = pyridine; TACN–Me_3_ = 1,4,7–trimethyl–1,4,7–triazacyclononane; Tp^−^ = hydrotrispyrazolylborate; TPA = tris(2–pyridyl)amine and tren = tris(2–aminoethyl)amine. ^a^ [Fe(III)] → [Fe(II)] process. ^b^ [Fe(II)] → [Fe(III)] process. ^c^ provided value for E_1/2_. ^d^ [Mn(II)] → [Mn(III)]. ^e^ [Mn(III)] → [Mn(II)]. ^f^ [Ru(III)Ru(III)] → [Ru(II)Ru(III)]. ^g^ [Ru(II)Ru(III)] → [Ru(II)Ru(II)]. ^h^ [Ru(III)Ru(III)] → [Ru(IV)Ru(III)].

**Table 9:** Cathodic and anodic potentials (in mV) for the redox couples [M(II)]/[M(I)] (M = Co, Ni and Cu) (E_pc1_, E_pa1_) for complexes bearing naproxen ligands.

Complex	E_pc_^a^	E_pa_^b^	Reference
[Co(NAP)_2_(MeOH)_4_]	–1109 ^a^	–13 ^b^	[43]
[Co(NAP)_2_(py)_2_(H_2_O)_2_]	–775	–25 ^b^	[43]
[Co(NAP)_2_(phen)(H_2_O)_2_]	–1296	+53 ^b^	[43]
[Co(NAP)_2_(bipy)(H_2_O)_2_]	–1065	–56 ^b^	[43]
[Ni(NAP)_2_(MeOH)_4_]	–607	–384	[61]
[Ni(NAP)_2_(bipy)(CH_3_OH)]	–544	–304	[61]
[Ni(NAP)_2_(phen)(H_2_O)]	–485	–307	[61]
[Ni(NAP)_2_(bipyam)]	–531	–314	[61]
[Ni(NAP)_2_(Hpko)_2_]	–473	–362	[61]
[Ni(NAP)_2_(py)_2_(H_2_O)_2_]	–524	–314	[61]
[Cu(NAP)_2_(4,7–dphphen)]	–355	Not provided	[55]

4,7–diPhphen = 4,7–diphenyl–1,10–phenanthroline; bipy = 2,2′–bipyridine; bipyam = 2,2′–bipyridylamine; Hpko = di(2–pyridyl)ketone oxime; phen = 1,10–phenanthroline and py = pyridine. ^a^ E_pc_ refers to [M(II)] → [M(I)] process. ^b^ E_pa_ refers to [M(I)] → [M(II)] process.

**Table 10:** In vitro cytotoxicity of reported compounds (IC_50_, in μM) against diverse cancer cell lines and normal cells (3T3–L1) after treatment for 48 h.

Compound	Cell Lines		Reference
HNAP	MCF–7: >160 HeLa: >160 3T3–L1: >250	A549: >160 MDA–MB–453: >100 HT–29: >100	[49,90,93]
[Μn_6_(NAP)(Hsal)(shi)_6_(py)_6_]	MCF–7: 9.6 ± 0.3 HeLa: 30.1 ± 1.3	A549: 69.3 ± 4.0	[90]
[Μn(NAP)_2_(CH_3_OH)]_n_	MCF–7: 62.0 ± 2.5 HeLa: >160	A549: >160	[90]
[Cu(NAP)(L1)Cl]	MCF–7: 1.51 ± 0.15		[49]
[Cu(NAP)(L2)Cl]	MCF–7: 31.03 ± 1.2		[49]
[Cu(NAP)(L3)Cl]	MCF–7: 10.40 ± 0.3		[49]
[Ag_4_(NAP)_4_(2pic)_2_]_n_	A549: 74.08 ± 1.05 3T3–L1: 224.87 ± 2.60	MDA–MB–453: 39.77 ± 1.95 HT–29: 29.96 ± 0.84	[49]
[Ag(NAP)(PPh_3_)_3_](H_2_O)	MCF–7: 0.7 ± 0.1		[50]
[Ag(NAP)(tptp)_2_]	MCF–7: 2.2 ± 0.2		[50]
[Ni(NAP)_2_(phen)(H_2_O)]	HepG2: >1000 HT 29: 35.50 ± 1.94	HEK–293: 198.5 ± 35.45 (72h)	[62]
[Co(NAP)_2_(cyclam)]	HMLER: 0.43 ± 0.05	HMLER–shEcad: 0.11 ± 0.03	[44]
[Au(NAP)(PPh_3_)]	HMLER: 0.183 ± 0.001 MDA–MB–231: 7.77 ± 0.41 4T1: 10.08 ± 0.86	HMLER–shEcad: 0.063 ± 0.006 MDA–MB–468: 6.48 ± 1.43	[51]
{[(*n*–Bu)_2_Sn]_2_(N1A)_2_(O)}_2_	MCF–7: 37.61 HeLa: 1.805 HepG2: 0.802	Colo205: 0.100 NCI–H460: 67.29	[88]
[(*n*–Bu)_3_Sn(N1A)]_n_	MCF–7: 0.301 HeLa: 0.361 HepG2: 0.127	Colo205: 0.104 NCI–H460: 0.188	[88]
H_3_shi	MCF–7: >160 HeLa: >160	A549: >160	[90]
PPh_3_	MCF–7: 67.4 ± 13.9		[50]
tptp	MCF–7: 26.5 ± 2.8		[50]
5–Fluorouracil	HMLER: 41.05 ± 5.30	HMLER–shEcad: 49.10 ± 5.94	[51]
Capecitabin	HMLER: >100	HMLER–shEcad: >100	[51]
Carboplatin	MCF–7: 26.83 HepG2: 0.613 A549: 39.43 ± 0.76 NCI–H460: 62.13 HT–29: 47.15 ± 2.80 HMLER: 67.31 ± 2.80	HeLa: 24.78 Colo205: 0.531 MDA–MB–453: 56.73 ± 1.24 HMLER–shEcad: 72.39± 7.99 3T3–L1: 43.20 ± 1.35	[3,51,88]
Cisplatin	MCF–7: 8.0 ± 0.7 HepG2: 23.71 ± 1.52 HT 29: 69.13 ± 1.88	HEK–293: 46.81 ± 2.79 HMLER–shEcad: 5.64 ± 0.30 HMLER: 2.56 ± 0.02	[49,50,51,62]
Doxorubicin	MCF–7: 10.90		[49]
Salinomycin	HMLER: 11.43 ± 0.42	HMLER–shEcad: 4.23 ± 0.35	[44,51]

2pic = 2–picoline; cyclam = 1,4,8,11–tetraazacyclotetradecane; H_2_sal = salicylic acid; H_3_shi = salicylhydroxamic acid; L1 = 4′–(4–tolyl)–2,2′:6′,2″–terpyridine; L2 = 4′–(furan–2–yl)–2,2′:6′,2″–terpyridine; L3 = 4′–(pyridin–3–yl)–2,2′:6′,2″–terpyridine; *n*–Bu = *n*–butyl; PPh_3_ = triphenylphosphine; py = pyridine and tptp = tri(p–tolyl)phosphine.

**Table 11:** SOD-like activity (IC_50_, in μM) of reported Cu(II)–naproxen complexes.

Compound	IC_50_ (μM)	Reference
[Cu_2_(NAP)_4_]_n_	0.3	[114]
[Cu(NAP)_2_(3pym)_2_]_n_	0.4	[114]
SOD enzyme	0.04–0.7	[114]

3pym = 3–pyridylmethanol; SOD = superoxide dismutase.

## Data Availability

Data sharing not applicable.

## Supplementary Materials

The following supporting information can be downloaded at https://www.mdpi.com/article/10.3390/molecules28052171/s1: Table S1: Characteristic bands in the IR spectra of selected complexes; Table S2: Anti-bacterial activity data (inhibition zone (IZ), in mm) of reported complexes for diverse concentrations; Table S3: DPPH scavenging activity (DPPH%, in %) of naproxen and its reported Cu(II), Co(II), Ni(II) and Mn(III) complexes for 20-min and 60-min incubation; Table S4: Hydroxyl radical scavenging activity (OH%, in %) of naproxen and its reported Cu(II), Co(II), Ni(II) and Mn(III) complexes; Table S5: ABTS radical scavenging activity (ABTS%, in %) of naproxen and its Cu(II), Co(II), Ni(II) and Mn(III) reported complexes. Table S6: In vitro inhibition of soybean lipoxygenase (LOX) activity (IC_50_, in μM) of naproxen and its reported Ni(II) and Ag(I) complexes; Table S7: DNA-binding constants (K_b_) for naproxen and its reported complexes; Table S8: BSA-quenching (k_q_) and binding constants (K) for naproxen and its reported complexes; Table S9: HSA-quenching (k_q_) and binding constants (K) for naproxen and its reported complexes [42,43,47,48,49,50,56,59,60,61,63,71,73,75,78,83,90,92,93,125].

## Abbreviations

1,2–dmimid = 1,2–dimethylimidazole; 2ampy = 2–aminopyridine; 2pic = 2–picoline; 3pic = 3–picoline; 3pym = 3–pyridylmethanol; 3U = 1,3–dipyridin–3–ylurea; 4,4′–bipy = 4,4′–bipyridine; 4,7–diPhphen = 4,7–diphenyl–1,10–phenanthroline; 4pic = 4–picoline; 4U = 1,3–dipyridin–4–ylurea; 5,5′–Me_2_–bipy = 5,5′–dimethyl–2,2′–bipyridine; ABTS = 2,2′–azino–bis(3–ethylbenzothiazoline–6–sulfonic acid); BHT = butylated hydroxytoluene; bipy = 2,2′–bipyridine; bipyam = 2,2′–bipyridylamine; bpp = 1,3–bis(4–pyridyl)propane; BSA = bovine serum albumin; caf = caffeine; CCDC = Cambridge Crystallographic Data Centre; CN = coordination number; COX = cyclooxygenase; CT = calf thymus; cyclam = 1,4,8,11–tetraazacyclotetradecane; DMF = *N,N*–dimethylformamide; DMSO = dimethylsulfoxide; DPPH = 1,1–diphenyl–picrylhydrazyl; DTA = differential thermogravimetric analysis; EB = ethidium bromide; EDA = *N,N*–dimethylethane–1,2–diimine; H_2_sal = salicylic acid; H_3_shi = salicylhydroxamic acid; H_5_dhpta = 1,3–diamino–2–hydroxypropane–N,N,N′,N′–tetraacetic acid; Hbzmd = 1H–benzimidazole; Hdmpz = 3,5–dimethylpyrazole; Himi = Imidazole; HN1A = 1–naphthylacetic acid; HN2A = 2–naphthylacetic acid; HNA = naphthylacetic acid; HNAP = naproxen, 6–methoxy–α–methyl–2–naphthaleneacetic acid; Hpko = di(2–pyridyl)ketone oxime; HPYA = 1–pyreneacetic acid; HSA = human serum albumin; IC_50_ = concentration that inhibits the survival of 50% of the cells/microorganisms; IZ = inhibition zone; K = SA-binding constant; K_b_ = DNA-binding constant; k_q_ = SA-quenching constant; L1 = 4′–(4–tolyl)–2,2′:6′,2″–terpyridine; L2 = 4′–(furan–2–yl)–2,2′:6′,2″–terpyridine; L3 = 4′–(pyridin–3–yl)–2,2′:6′,2″–terpyridine; L4 = 4′–(4–chlorophenyl)–2,2′:6′,2″–terpyridine; L5 = 4′–(3,4–dimethoxyphenyl)–2,2′:6′,2″–terpyridine; L6 = 4′–(4–dimethylaminophenyl)–2,2′:6′,2″–terpyridine; LOX = lipoxygenase; Me *=* methyl; MIC = minimum inhibitory concentration; *n*–Bu = *n*–butyl; NDGA = nordihydroguairetic acid; neoc = neocuproine, 2,9–dimethyl–1,10–phenanthroline; NSAID = non-steroidal anti-inflammatory drug; PDB = protein database; pDNA = supercoiled pBR322 plasmid DNA; Ph = phenyl; phdat = 2,4–diamine–6–phenyl–1,3,5–triazine; phen = 1,10–phenanthroline; PPh_3_ = triphenylphosphine; py = pyridine; pyr3OH = pyridin–3–ol; RT = room temperature; SA = serum albumin; SOD = superoxide dismutase; TACN–Me_3_ = 1,4,7–trimethyl–1,4,7–triazacyclononane; TGA *=* thermogravimetric analysis; THF = tetrahydrofuran; Tp^–^ = hydrotrispyrazolylborate; TPA = tris(2–pyridyl)amine; tptp = tri(p–tolyl)phosphine; tren = tris(2–aminoethyl)amine; trolox = 6–hydroxy–2,5,7,8– tetramethylchromane–2–carboxylic acid; VT = variable temperature; Δ*ν*(COO) = *ν*_asym_(COO) − *ν*_sym_(COO); *ν*_asym_(COO) = antisymmetric stretching vibration of carboxylato group; *ν*_sym_(COO) = symmetric stretching vibration of carboxylato group.
