# Supplementary material for: Metal Complexes with Naphthalene-Based Acetic Acids as Ligands: Structure and Biological Activity
Source: Molecules. 2023 Feb 26;28(5):2171. doi: 10.3390/molecules28052171 (PMC10005298; doi:10.3390/molecules28052171)
Supplement: Supplementary file 1 [file molecules-28-02171-s001.zip › molecules-2215277-supplementary.pdf]

INDEX

Content	Page
**Table S1**. Characteristic bands in the IR spectra of selected complexes.	3
**Table S2**. Anti-bacterial activity data (inhibition zone (IZ), in mm) of reported complexes for diverse concentrations	5
**Table S3**. DPPH-scavenging activity (DPPH%, in %) of naproxen and its reported Cu(II), Co(II), Ni(II) and Mn(III) complexes for 20-min and 60-min incubation.	7
**Table S4**. Hydroxyl radical scavenging activity (OH%, in %) of naproxen and its reported Cu(II), Co(II), Ni(II) and Mn(III) complexes.	8
**Table S5**. ABTS radical scavenging activity (ABTS%, in %) of naproxen and its Cu(II), Co(II), Ni(II) and Mn(III) reported complexes.	9
**Table S6**. *In vitro* inhibition of soybean lipoxygenase (LOX) activity (IC_{50}, in μM) of naproxen and its reported Ni(II) and Ag(I) complexes.	10
**Table S7**. DNA-binding constants (K_b) for naproxen and its reported complexes.	11
**Table S8**. BSA-quenching (k_q) and binding constants (K) for naproxen and its reported complexes.	13
**Table S9**. HSA-quenching (k_q) and binding constants (K) for naproxen and its reported complexes.	14
**References**	15

Table S1. Characteristic bands in the IR spectra of selected complexes. The values of v_{asym}(COO), v_{sym}(COO) and Δv(COO) (in cm^{-1}) are cited as given in the relevant references.

**Complex**	**v_{asym}(COO)**	**v_{sym}(COO)**	**Δv(COO)**	**Ref.**
I Monodentate binding				
[Mn(NAP-O)$_2$(py)$_2$(H$_2$O)$_2$]	1601	1388	213	1
[Co(NAP-O)$_2$(CH$_3$OH)$_4$]	1604	1398	206	2
[Co(NAP-O)$_2$(py)$_2$(H$_2$O)$_2$]	1605	1389	216	2
[Co(NAP-O)$_2$(phen)(CH$_3$OH)$_2$]	1609	1398	211	2
[Co(NAP-O)$_2$(bipy)(CH$_3$OH)$_2$]	1605	1401	204	2
[Ni(NAP-O)$_2$(CH$_3$OH)$_4$]	1604	1391	213	3
[Ni(NAP-O)$_2$(Hpko-N,N′)$_2$]	1604	1392	212	3
[Ni(NAP-O)$_2$(py)$_2$(H$_2$O)$_2$]	1604	1390	204	3
[Cu(NAP-O)$_2$(bipy)]·H$_2$O	1600	1379	221	4
[Cu(NAP-O)$_2$(phen)]·H$_2$O	1608	1377	231	4
[Cu(NAP-O)$_2$(py)$_2$(H$_2$O)]	1595	1389	206	4
[Cu(NAP-O)$_2$(H$_2$O)(4pic)$_2$]	1591	1380	211	5
[Cu(N1A-O)(EDA)$_2$](ClO$_4$)	1598	1381	217	6
[Cu(NAP-O)(L1-6)Cl]	1556-1564	1370-1398	<200	7
[Zn(NAP-O)$_2$(phen)]			215	8
[Zn(NAP-O)$_2$(neoc)]			230	8
[Zn(NAP-O)$_2$(2ampy)$_2$]			216	8
[Zn(NAP-O)$_2$(Himi)$_2$]			196	8
[Zn(NAP-O)$_2$(1,2-dmimid)$_2$]			229	8
[Ag(NAP-O)(PPh$_3$)$_3$]·H$_2$O	1573	1393	180	9
II Bidentate chelating				
[Ni(NAP-O,O′)$_2$(bipyam)]	1605	1421	184	3
[Ag(NAP-O,O′)(tptp)$_2$]	1560	1393	167	9
[(n-Bu)$_2$Sn(NAP-O,O′)$_2$]	1584	1389	195	10
III Monodentate binding + Bidentate chelating				
[Mn(NAP-O)(NAP-O,O′) (phen)(H$_2$O)]	1604	1426, 1394	178, 201	1
[Ni(NAP-O)(NAP-O,O′)(bipy)(CH$_3$OH)]	1606	1420, 1401	186, 205	3
[Ni(NAP-O)(NAP-O,O′)(phen)(H$_2$O)]	1606	1422, 1400	186, 206	3

IV bridging				
[Mn(μ-NAP-O,O′)$_2$(CH$_3$OH)]$_n$	1566	1393	173	11
[Cu$_2$(μ-NAP-O,O′)$_4$(H$_2$O)$_2$]	1593	1424	169	4
[Cu$_2$(μ-NAP-O,O′)$_4$(3pic)$_2$]	1604	1398	206	5
[Mn$_6$(μ$_3$-NAP-O,O,O′)(μ$_2$-Hsal-O,O′)(μ$_2$-shi-N,O)$_5$(py)$_6$]	1570	1390	180	11
[Ru$_2$(μ$_2$-N1A-O,O′)$_4$(THF)$_2$](PF$_6$).THF	1598	1411	187	12
[Ru$_2$(μ$_2$-N2A-O,O′)$_4$(H$_2$O)$_2$](PF$_6$).THF	1601	1409	192	12
[Ag$_4$(μ-NAP-O,O′)$_4$(2pic)$_2$]$_n$	1531	1379	152	13
[(Me$_3$Sn)$_4$(μ$_2$-NAP-O,O′)$_4$]	1603	1388	215	14
[(Bu$_3$Sn)$_4$(μ$_2$-NAP-O,O′)$_4$]	1644	1439	205	14
[(Ph$_3$Sn)(μ$_2$-NAP-O,O′)]$_n$	1632	1425	207	14
[Y$_2$(μ$_2$-N1A-O,O,O′)$_2$(μ$_2$-N1A-O,O′)$_4$(phen)$_2$].DMF	1563	1425	138	15
[Pr$_2$(μ$_2$-N1A-O,O,O′)$_2$(μ$_2$-N1A-O,O′)$_4$(phen)$_2$].DMF	1566	1380	186	15
[Nd$_2$(μ$_2$-N1A-O,O,O′)$_2$(μ$_2$-N1A-O,O′)$_4$(phen)$_2$]	1545	1401	144	16
[Sm$_2$(μ$_2$-N1A-O,O,O′)$_2$(μ$_2$-N1A-O,O′)$_4$(phen)$_2$].DMF	1545	1425	120	15
[Eu$_2$(μ$_2$-N1A-O,O,O′)$_2$(μ$_2$-N1A-O,O′)$_4$(phen)$_2$].DMF	1556	1422	134	15
[Gd$_2$(μ$_2$-N1A-O,O,O′)$_2$(μ$_2$-N1A-O,O′)$_4$(phen)$_2$].DMF	1563	1425	138	15
[Tb$_2$(μ$_2$-N1A-O,O,O′)$_2$(μ$_2$-N1A-O,O′)$_4$(phen)$_2$].DMF	1562	1422	140	15
[Yb$_2$(μ$_2$-N1A-O,O,O′)$_2$(μ$_2$-N1A-O,O′)$_4$(phen)$_2$].DMF	1578	1425	153	15

1,2-dmimid = 1,2-dimethylimidazole; 2pic = 2-picoline; 4pic = 4-picoline; bipy = 2,2′-bipyridine; bipyam = 2,2′-bipyridylamine; DMF = N,N-dimethylformamide; EDA = N,N-dimethylethane-1,2-diamine; Himi = imidazole; Hpko = di(2-pyridyl)ketone oxime; H$_2$sal = salicylic acid; H$_3$shi = salicylhydroxamic acid; neoc = neocuproine; phen = 1,10-phenantholine; py = pyridine; THF = tetrahydrofuran; tptp = tri(p-tolyl)phosphine.

Table S2. Anti-bacterial activity data (inhibition zone (IZ), in mm) of reported complexes for diverse concentrations.

Compound	C	*BS*	*ML*	*SA*	*EC*	*KP*	*PA*	*PM*	Reference
Naproxen	8.5 mM	NT	NA	NA	NA	NA	NA	NA	8
$[Zn_2(NAP)_4]$	8.5 mM	NT	NA	NA	10.0±1.0	10.0±1.0	NA	8.0±0.6	8
$[Zn(NAP)_2(phen)]$	8.5 mM	NT	16.0±2.1	16.0±1.1	20.0±2.1	20.0±2.5	19.0±1.1	21.0±1.0	8
$[Zn(NAP)_2(neoc)]$	8.5 mM	NT	20.0±1.5	20.0±1.0	7.7±1.2	11.0±1.2	NA	NA	8
$[Zn(NAP)_2(2ampy)_2]$	8.5 mM	NT	12.3±1.2	12.3±0.6	NA	NA	NA	NA	8
$[Zn(NAP)_2(Himi)2]$	8.5 mM	NT	NA	NA	9.7±0.6	9.7±0.6	NA	NA	8
$[Zn(NAP)_2(1,2\text{-dmimid})_2]$	8.5 mM	NT	8.7±0.6	8.3±1.2	NA	NA	NA	NA	8
Erythromycin	8.5 mM	NT	39.0	43.0	18.0	21.0	NA	17.0	8
Gentamycin	8.5 mM	NT	33.0	27.0	28.0	32.0	23.0	28.0	8
HN1A	4 mg/mL	25	NT	19.8	16.0	NT	NT	NT	17
	1.04 mM	8.0-10.0	NT	8.2-9.2	7.0-12.0	NT	NT	NT	15, 18
	2.60 mM	11.0	NT	10.0	14.0	NT	NT	NT	15, 18
	4.16 mM	10.8-14.0	NT	10.8-15.0	16.0-18.0	NT	NT	NT	15, 18
$[Co(N1A)_2(H_2O)_2]_n$	4 mg/mL	23.3	NT	15.5	21.5	NT	NT	NT	17
$[Gd_2(N1A)_6(phen)_2]$	1.04 mM	16.0	NT	11.5	11.0	NT	NT	NT	18
	2.60 mM	17.5	NT	18.0	16.2	NT	NT	NT	18
	4.16 mM	19.0	NT	19.0	19.0	NT	NT	NT	18
$[Tb_2(N1A)_6(phen)_2].DMF$	1.04 mM	17.1	NT	15.5	14.3	NT	NT	NT	18
	2.60 mM	20.8	NT	27.5	24.0	NT	NT	NT	18
	4.16 mM	22.5	NT	26.4	22.8	NT	NT	NT	18
$[Y_2(N1A)_6(phen)_2]\cdot 2DMF$	1.04 mM	9.0	NT	9.2	17.0	NT	NT	NT	15
	2.60 mM	15.0	NT	19.1	18.1	NT	NT	NT	15
	4.16 mM	16.3	NT	18.2	16.5	NT	NT	NT	15

$[Eu_2(N1A)_6(phen)_2]\cdot 2DMF$	1.04 mM	11.0	NT	10.2	13.1	NT	NT	NT	15
	2.60 mM	12.0	NT	16.1	15.0	NT	NT	NT	15
	4.16 mM	12.0	NT	14.5	15.0	NT	NT	NT	15
$[Sm_2(N1A)_6(phen)_2]\cdot 2DMF$	1.04 mM	14.1	NT	16.0	14.0	NT	NT	NT	15
	2.60 mM	15.0	NT	18.1	18.5	NT	NT	NT	15
	4.16 mM	18.9	NT	23.0	19.0	NT	NT	NT	15
$[Tb_2(N1A)_6(phen)_2]\cdot 2DMF$	1.04 mM	15.1	NT	15.5	14.3	NT	NT	NT	15
	2.60 mM	20.8	NT	27.5	24.0	NT	NT	NT	15
	4.16 mM	22.5	NT	26.4	22.8	NT	NT	NT	15
$[Gd_2(N1A)_6(phen)_2]\cdot 2DMF$	1.04 mM	9.2	NT	12.0	10.1	NT	NT	NT	15
	2.60 mM	14.0	NT	15.2	19.8	NT	NT	NT	15
	4.16 mM	16.1	NT	16.9	20.0	NT	NT	NT	15
$[Pr_2(N1A)_6(phen)_2]\cdot 2DMF$	1.04 mM	13.0	NT	12.1	12.0	NT	NT	NT	15
	2.60 mM	15.4	NT	17.2	15.3	NT	NT	NT	15
	4.16 mM	18.2	NT	14.6	14.3	NT	NT	NT	15
$[Yb_2(N1A)_6(phen)_2]$	1.04 mM	10.1	NT	16.1	15.0	NT	NT	NT	15
	2.60 mM	15.1	NT	20.0	21.0	NT	NT	NT	15
	4.16 mM	18.1	NT	20.9	19.2	NT	NT	NT	15
$[Cu_2(N1A)_4(Himi)_4]$	0.01 mg/mL	12.0	NT	NT	13.0	NT	NT	NT	19
	0.05 mg/mL	12.0	NT	NT	15.0	NT	NT	NT	19
	0.10mg/mL	13.0	NT	NT	10.5	NT	NT	NT	19
	0.50 mg/mL	12.0	NT	NT	14.5	NT	NT	NT	19
	1.00 mg/mL	16.0	NT	NT	15.0	NT	NT	NT	19

Abbreviations for bacteria: *BS = Bacillus subtilis; EC = Escherichia coli; KP = Klebsiella pneumoniae; ML = Micrococcus luteus; PA = Pseudomonas aeruginosa; PM = Proteus mirabilis; SA = Staphylococcus aureus*

NA = not active

NT = not tested

Table S3. DPPH-scavenging activity (DPPH%, in %) of naproxen and its reported Cu(II), Co(II), Ni(II) and Mn(III) complexes for 20-min and 60-min incubation. BHT and NDGA were the reference compounds used in the studies. Each experiment was performed at least in triplicate SD < ±10%.

Compound	Code [a]	DPPH%, 20 min	DPPH%, 60 min	Ref
Naproxen	HNAP	8.03±0.32	8.43±0.20	2
$[Cu_2(NAP)_4(H_2O)_2]$	Cu1	not given	20.16±0.23	2
$[Cu(NAP)_2(py)_2(H_2O)]$	Cu2	not given	18.66±0.29	2
$[Cu(NAP)_2(phen)]\cdot H_2O$	Cu3	not given	18.76±0.13	2
$[Cu(NAP)_2(bipy)]\cdot H_2O$	Cu4	not given	19.48±0.12	2
$[Co(NAP)_2(CH_3OH)_4]$	Co1	not given	20.37±0.23	2
$[Co(NAP)_2(py)_2(H_2O)_2]$	Co2	not given	18.90±0.22	2
$[Co(NAP)_2(phen)(H_2O)_2]$	Co3	not given	42.42±0.13	2
$[Co(NAP)_2(bipy)(H_2O)_2]$	Co4	not given	26.98±0.41	2
$[Ni(NAP)_2(CH_3OH)_4]$	Ni1	16.47±0.48	16.53±0.41	3
$[Ni(NAP)_2(bipy)(CH_3OH)]$	Ni2	12.51±0.45	13.61±0.23	3
$[Ni(NAP)_2(phen)(H_2O)]$	Ni3	15.67±0.61	14.48±0.34	3
$[Ni(NAP)_2(bipyam)]$	Ni4	8.56±0.81	10.21±0.63	3
$[Ni(NAP)_2(Hpko)_2]$	Ni5	12.28±0.34	13.45±0.46	3
$[Ni(NAP)_2(py)_2(H_2O)_2]$	Ni6	14.51±0.73	15.91±0.45	3
$[Mn(NAP)_2(py)_2(H_2O)_2]$	Mn1	13.58±076	16.76±0.34	1
$[Mn(NAP)_2(phen)(H_2O)]$	Mn2	14.67±0.37	16.06±0.62	1
BHT	BHT	31.30±0.10	60.00±0.38	2
NDGA	NDGA	81.02±0.18	82.60±0.17	2

[a] Abbreviation of the compounds used in Figures 23 and 24.

BHT = butylated hydroxytoluene; bipy = 2,2′-bipyridine; bipyam = 2,2′-bipyridylamine; HNAP = naproxen; Hpko = di(2-pyridyl)ketone oxime; NDGA = nordihydroguairetic acid; phen = 1,10-phenantholine; py = pyridine.

Table S4. Hydroxyl radical scavenging activity (OH%, in %) of naproxen and its reported Cu(II), Co(II), Ni(II) and Mn(III) complexes. Trolox was the reference compound used in the studies. Each experiment was performed at least in triplicate SD < ±10%.

Compound	Code [a]	OH%	Ref
Naproxen	HNAP	89.55±0.44	2
$[Cu_2(NAP)_4(H_2O)_2]$	Cu1	72.84±0.05	2
$[Cu(NAP)_2(py)_2(H_2O)]$	Cu2	93.40±0.22	2
$[Cu(NAP)_2(phen)]\cdot H_2O$	Cu3	93.26±0.36	2
$[Cu(NAP)_2(bipy)]\cdot H_2O$	Cu4	80.11±0.17	2
$[Co(NAP)_2(CH_3OH)_4]$	Co1	96.75±0.30	2
$[Co(NAP)_2(py)_2(H_2O)_2]$	Co2	84.98±0.35	2
$[Co(NAP)_2(phen)(H_2O)_2]$	Co3	92.46±0.22	2
$[Co(NAP)_2(bipy)(H_2O)_2]$	Co4	90.21±0.19	2
$[Ni(NAP)_2(CH_3OH)_4]$	Ni1	96.53±0.32	3
$[Ni(NAP)_2(bipy)(CH_3OH)]$	Ni2	90.65±0.82	3
$[Ni(NAP)_2(phen)(H_2O)]$	Ni3	80.12±0.75	3
$[Ni(NAP)_2(bipyam)]$	Ni4	76.39±0.20	3
$[Ni(NAP)_2(Hpko)_2]$	Ni5	92.69±0.22	3
$[Ni(NAP)_2(py)_2(H_2O)_2]$	Ni6	96.78±0.45	3
$[Mn(NAP)_2(py)_2(H_2O)_2]$	Mn1	98.31±0.67	1
$[Mn(NAP)_2(phen)(H_2O)]$	Mn2	97.42±0.83	1
Trolox		82.80±0.13	2

[a] Abbreviation of the compounds used in Figure 25.

bipy = 2,2′-bipyridine; bipyam = 2,2′-bipyridylamine; HNAP = naproxen; Hpko = di(2-pyridyl)ketone oxime; phen = 1,10-phenantholine; py = pyridine; trolox = 6-hydroxy-2,5,7,8-tetramethylchromane-2-carboxylic acid.

Table S5. ABTS radical scavenging activity (ABTS%, in %) of naproxen and its Cu(II), Co(II), Ni(II) and Mn(III) reported complexes. Trolox was the reference compound used in the studies. Each experiment was performed at least in triplicate SD < ±10%.

Compound	Code [a]	ABTS%	Ref
Naproxen	HNAP	87.51±0.17	2
$[Cu_2(NAP)_4(H_2O)_2]$	Cu1	77.74±0.39	2
$[Cu(NAP)_2(py)_2(H_2O)]$	Cu2	92.12±0.13	2
$[Cu(NAP)_2(phen)]\cdot H_2O$	Cu3	82.39±0.25	2
$[Cu(NAP)_2(bipy)]\cdot H_2O$	Cu4	76.44±0.20	2
$[Co(NAP)_2(CH_3OH)_4]$	Co1	82.46±0.35	2
$[Co(NAP)_2(py)_2(H_2O)_2]$	Co2	90.22±0.28	2
$[Co(NAP)_2(phen)(H_2O)_2]$	Co3	87.32±0.17	2
$[Co(NAP)_2(bipy)(H_2O)_2]$	Co4	84.54±0.29	2
$[Ni(NAP)_2(CH_3OH)_4]$	Ni1	96.03±0.43	3
$[Ni(NAP)_2(bipy)(CH_3OH)]$	Ni2	88.82±0.60	3
$[Ni(NAP)_2(phen)(H_2O)]$	Ni3	85.74±0.21	3
$[Ni(NAP)_2(bipyam)]$	Ni4	84.59±0.92	3
$[Ni(NAP)_2(Hpko)_2]$	Ni5	97.73±0.27	3
$[Ni(NAP)_2(py)_2(H_2O)_2]$	Ni6	87.72±0.54	3
$[Mn(NAP)_2(py)_2(H_2O)_2]$	Mn1	94.78±0.36	1
$[Mn(NAP)_2(phen)(H_2O)]$	Mn2	96.67±0.68	1
Trolox		91.8±0.17	2

[a] Abbreviation of the compounds used in Figure 26.

bipy = 2,2′-bipyridine; bipyam = 2,2′-bipyridylamine; HNAP = naproxen; Hpko = di(2-pyridyl)ketone oxime; phen = 1,10-phenantholine; py = pyridine; trolox = 6-hydroxy-2,5,7,8-tetramethylchromane-2-carboxylic acid.

Table S6. *In vitro* inhibition of soybean lipoxygenase (LOX) activity (IC_{50}, in μM) of naproxen and its reported Ni(II) and Ag(I) complexes. Caffeic acid and cisplatin were the reference compounds used in the studies. Each experiment was performed at least in triplicate SD < ±10%.

**Compound**	**Code** [a]	**LOX, IC_{50} (μM)**	**Reference**
Naproxen	HNAP	56.61±0.71	3
$[Ni(NAP)_2(CH_3OH)_4]$	Ni1	22.62±0.41	3
$[Ni(NAP)_2(bipy)(CH_3OH)]$	Ni2	48.56±0.08	3
$[Ni(NAP)_2(phen)(H_2O)]$	Ni3	45.77±0.67	3
$[Ni(NAP)_2(bipyam)]$	Ni4	34.63±0.78	3
$[Ni(NAP)_2(Hpko)_2]$	Ni5	34.67±0.28	3
$[Ni(NAP)_2(py)_2(H_2O)_2]$	Ni6	37.22±0.23	3
$[Ag(NAP)(PPh_3)_3](H_2O)$	Ag1	5.1	9
$[Ag(NAP)(tptp)_3](H_2O)$	---	> 30	9
Caffeic acid	CA	600±0.3	3
Cisplatin	cisPt	65.9	9

[a] Abbreviation of the compounds used in Figure 27.

bipy = 2,2′-bipyridine; bipyam = 2,2′-bipyridylamine; HNAP = naproxen; Hpko = di(2-pyridyl)ketone oxime; phen = 1,10-phenantholine; py = pyridine; tptp = tri(p-tolyl)phosphine.

Table S7. DNA-binding constants (K_b) for naproxen and its reported complexes.

**Compound**	**Code** [a]	**K_b (M^{-1})**	**Reference**
Naproxen	HNAP	$2.67(\pm0.22)\times10^4$	4
$[Cu_2(NAP)_4(H_2O)_2]$	Cu1	$2.27(\pm0.51)\times10^4$	4
$[Cu(NAP)_2(bipy)]$	Cu2	$3.86(\pm0.25)\times10^4$	4
$[Cu(NAP)_2(phen)]$	Cu3	$9.20(\pm0.30)\times10^3$	4
$[Cu(NAP)_2(py)_2(H_2O)]$	Cu4	$8.97(\pm0.50)\times10^3$	4
[Cu(NAP)(L1)Cl]	Cu5	$2.38(\pm0.17)\times10^5$	7
[Cu(NAP)(L2)Cl]	Cu6	$2.24(\pm0.25)\times10^5$	7
[Cu(NAP)(L3)Cl]	Cu7	$4.27(\pm0.19)\times10^5$	7
$[Co(NAP)_2(CH_3OH)_4]$	Co1	$3.15(\pm0.57)\times10^4$	2
$[Co(NAP)_2(py)_2(H_2O)_2]$	Co2	$2.29(\pm0.45)\times10^4$	2
$[Co(NAP)_2(phen)(H_2O)_2]$	Co3	$2.76(\pm0.63)\times10^4$	2
$[Co(NAP)_2(bipy)(H_2O)_2]$	Co4	$3.58(\pm0.07)\times10^4$	2
$[Ni(NAP)_2(CH_3OH)_4]$	Ni1	$1.47(\pm0.05)\times10^5$	3
$[Ni(NAP)_2(bipy)(CH_3OH)]$	Ni2	$5.96(\pm0.10)\times10^5$	3
$[Ni(NAP)_2(phen)(H_2O)]$	Ni3	$1.54(\pm0.12)\times10^5$	3
$[Ni(NAP)_2(bipyam)]$	Ni4	$2.91(\pm0.31)\times10^5$	3
$[Ni(NAP)_2(Hpko)_2]$	Ni5	$8.01(\pm0.27)\times10^5$	3
$[Ni(NAP)_2(py)_2(H_2O)_2]$	Ni6	$6.14(\pm0.10)\times10^5$	3
$[Mn(NAP)_2(CH_3OH)]_n$	Mn1	$2.26(\pm0.08)\times10^5$	11
$[Mn(NAP)_2(phen)(H_2O)]$	Mn2	$6.40(\pm0.17)\times10^6$	1
$[Mn(NAP)_2(py)(H_2O)_2]$	Mn3	$2.29(\pm0.13)\times10^4$	1
$[Mn_6(NAP)(Hsal)(shi)_6(py)_6]$	Mn4	$3.07(\pm0.12)\times10^4$	11
$[(n\text{-Bu})_2Sn(NAP)_2]$	Sn1	$5.9(\pm0.6)\times10^4$	10
$[Ag(NAP)(PPh_3)_3](H_2O)$	Ag1	$3.28(\pm0.85)\times10^5$	9
$[Ag(NAP)(tptp)_2]$	Ag2	$4.7(\pm1.8)\times10^4$	9
Ethidium bromide	EB	$1.23(\pm0.07)\times10^5$	20

[a] Abbreviation of the compounds used in Figure 28.

bipy = 2,2′-bipyridine; bipyam = 2,2′-bipyridylamine; EB = ethidium bromide; H_2sal = salicylic acid; H_3shi = salicylhydroxamic acid; HNAP = naproxen; Hpko = di(2-pyridyl)ketone oxime; L1 = 4'–(4–tolyl)–2,2':6',2''–terpyridine; L2 = 4'–(furan–2–yl)–2,2':6',2''–terpyridine; L3 = 4′–(pyridin–3–yl)–2,2′:6′,2′′–terpyridine; *n*–Bu = *n*–butyl; phen = 1,10-phenantholine; PPh_3 = triphenylphosphine; py = pyridine; tptp = tri(p–tolyl)phosphine.

Table S8. BSA-quenching (k_q) and binding constants (K) for naproxen and its reported complexes.

**Compound**	**Code** [a]	**$k_{q(BSA)}$ (M^{-1} s^{-1})**	**$K_{(BSA)}$ (M^{-1})**	**Ref**
Naproxen	HNAP	$1.18(\pm0.06)\times10^{12}$	5.35×10^{3}	4
$[Cu_2(NAP)_4(H_2O)_2]$	Cu1	$2.34(\pm0.15)\times10^{12}$	6.61×10^{4}	4
$[Cu(NAP)_2(bipy)]$	Cu2	$2.24(\pm0.14)\times10^{12}$	1.20×10^{4}	4
$[Cu(NAP)_2(phen)]$	Cu3	$2.94(\pm0.09)\times10^{12}$	1.90×10^{4}	4
$[Cu(NAP)_2(py)_2(H_2O)]$	Cu4	$2.33(\pm0.14)\times10^{12}$	2.55×10^{4}	4
$[Co(NAP)_2(CH_3OH)_4]$	Co1	$2.47(\pm0.16)\times10^{12}$	1.25×10^{5}	2
$[Co(NAP)_2(py)_2(H_2O)_2]$	Co2	$1.21(\pm0.04)\times10^{12}$	2.64×10^{4}	2
$[Co(NAP)_2(phen)(H_2O)_2]$	Co3	$2.09(\pm0.15)\times10^{12}$	1.07×10^{5}	2
$[Co(NAP)_2(bipy)(H_2O)_2]$	Co4	$1.75(\pm0.09)\times10^{12}$	3.06×10^{4}	2
$[Ni(NAP)_2(CH_3OH)_4]$	Ni1	$2.46(\pm0.12)\times10^{12}$	$4.51(\pm0.34)\times10^{4}$	3
$[Ni(NAP)_2(bipy)(CH_3OH)]$	Ni2	$2.42(\pm0.24)\times10^{12}$	$3.25(\pm0.31)\times10^{5}$	3
$[Ni(NAP)_2(phen)(H_2O)]$	Ni3	$5.90(\pm0.32)\times10^{12}$	$4.18(\pm0.34)\times10^{5}$	3
$[Ni(NAP)_2(bipyam)]$	Ni4	$8.28(\pm0.22)\times10^{12}$	$4.59(\pm0.35)\times10^{4}$	3
$[Ni(NAP)_2(Hpko)_2]$	Ni5	$4.50(\pm0.28)\times10^{12}$	$1.08(\pm0.07)\times10^{5}$	3
$[Ni(NAP)_2(py)_2(H_2O)_2]$	Ni6	$9.52(\pm0.43)\times10^{11}$	$7.44(\pm0.55)\times10^{3}$	3
$[Mn(NAP)_2(py)_2(H_2O)_2]$	Mn1	$1.35(\pm0.10)\times10^{12}$	$9.99(\pm0.45)\times10^{5}$	1
$[Mn(NAP)_2(phen)(H_2O)]$	Mn2	$2.39(\pm0.07)\times10^{12}$	$1.61(\pm0.25)\times10^{4}$	1
$[Mn(NAP)_2(CH_3OH)]_n$	Mn3	$2.05(\pm0.05)\times10^{12}$	$3.51(\pm0.12)\times10^{4}$	11
$[Mn_6(NAP)(Hsal)(shi)_6(py)_6]$	Mn4	$1.06(\pm0.03)\times10^{12}$	$1.03(\pm0.04)\times10^{5}$	11
$[Nd_2(N1A)_6(phen)_2]$	-	not reported	$2.41\times10^{4} – 3.03\times10^{4}$	16

[a] Abbreviation of the compounds used in Figures 29 and 30.

Table S9. HSA-quenching (k_q) and binding constants (K) for naproxen and its reported complexes.

**Compound**	**Code** [a]	$k_{q(HSA)}$ (M^{-1} s^{-1})	$K_{(HSA)}$ (M^{-1})	**Ref**
Naproxen	HNAP	1.24(±0.09)×10^{12}	3.27×10^{4}	4
[$Cu_2(NAP)_4(H_2O)_2$]	Cu1	4.73(±0.37)×10^{12}	7.83×10^{4}	4
[$Cu(NAP)_2$(bipy)]	Cu2	4.70 (±0.17)×10^{12}	3.20×10^{4}	4
[$Cu(NAP)_2$(phen)]	Cu3	4.02(±0.21)×10^{12}	7.69×10^{4}	4
[$Cu(NAP)_2(py)_2(H_2O)$]	Cu4	4.56(±0.27)×10^{12}	9.55×10^{3}	4
[$Co(NAP)_2(CH_3OH)_4$]	Co1	2.42(±0.10)×10^{12}	3.20×10^{4}	2
[$Co(NAP)_2(py)_2(H_2O)_2$]	Co2	1.60(±0.07)×10^{12}	2.69×10^{4}	2
[$Co(NAP)_2$(phen)$(H_2O)_2$]	Co3	4.23(±0.27)×10^{12}	1.58×10^{4}	2
[$Co(NAP)_2$(bipy)$(H_2O)_2$]	Co4	6.28(±0.36)×10^{12}	2.19×10^{4}	2
[$Ni(NAP)_2(CH_3OH)_4$]	Ni1	6.57(±0.37)×10^{12}	1.35(±0.11)×10^{4}	3
[$Ni(NAP)_2$(bipy)(CH_3OH)]	Ni2	2.37(±0.29)×10^{12}	1.93(±0.03)×10^{5}	3
[$Ni(NAP)_2$(phen)(H_2O)]	Ni3	1.13(±0.06)×10^{13}	2.73(±0.25)×10^{4}	3
[$Ni(NAP)_2$(bipyam)]	Ni4	1.59(±0.05)×10^{13}	1.88(±0.26)×10^{5}	3
[$Ni(NAP)_2(Hpko)_2$]	Ni5	4.71(±0.13)×10^{12}	3.02(±0.41)×10^{4}	3
[$Ni(NAP)_2(py)_2(H_2O)_2$]	Ni6	1.61(±0.08)×10^{13}	4.05(±0.23)×10^{4}	3
[$Mn(NAP)_2(py)_2(H_2O)_2$]	Mn1	2.25(±0.12)×10^{12}	6.50(±0.30)×10^{4}	1
[$Mn(NAP)_2$(phen)(H_2O)]	Mn2	9.06(±0.26)×10^{12}	3.30(±0.28)×10^{5}	1

[a] Abbreviation of the compounds used in Figures 29 and 30.

References

1 Dimiza, F.; Raptopoulou, C.P.; Psycharis, V.; Papadopoulos, A.N.; Psomas, G. Manganese(II) complexes with the non–steroidal anti–inflammatory drugs naproxen and mefenamic acid. Synthesis, structure, antioxidant capacity, interaction with albumins and DNA. *New J. Chem.*, **2018**, 42, 16666–16681.

2 Dimiza, F.; Papadopoulos, A.N.; Tangoulis, V.; Psycharis, V.; Raptopoulou, C.P.; Kessissoglou, D.P. Psomas, G. Biological evaluation of cobalt(II) complexes with non–steroidal anti–inflammatory drug naproxen. *J. Inorg. Biochem*., **2012**, 107, 54–64.

3 Totta, X.; Hatzidimitriou, A.G.; Papadopoulos, A.N.; Psomas, G. Nickel(II)–naproxen mixed–ligand complexes: Synthesis, structure, antioxidant activity and interaction with albumins and calf–thymus DNA. *New J. Chem*., **2017**, 41, 4478–4492.

4 Dimiza, F.; Perdih, F.; Tangoulis, V.; Turel, I.; Kessissoglou, D.P.; Psomas, G. Interaction of copper(II) with the non–steroidal anti–inflammatory drugs naproxen and diclofenac: Synthesis, Structure, DNA– and albumin–binding. *J. Inorg. Biochem*., **2011**, 105, 476–489.

5 Caglar, S.; Adiguzel, E.; Sariboga, B.; Temel, E.; Buyukgungor, O. Mono and dinuclear copper(II) naproxenato complexes containing 3–picoline and 4–picoline: synthesis, structure, properties, catechol oxidase, and antimicrobial activities. *J. Coord. Chem*., **2014**, 67, 670–683.

6 Sheng, G.; Cheng, X.; You, Z.; Zhu, H. Syntheses, Crystal Structures, and Characterization of Copper(II) and Zinc(II) Complexes Derived from N,N–Dimethylethane–1,2–diamine and Phenylacetic Acid Derivatives. *Synth. React. Inorg., Metal–Org., Nano–Met. Chem*., **2015**, 45, 1273–1277.

7 Mahendiran, D.; Gurumoorthy, P.; Gunasekaran, K.; Kumar, R.S.; Rahiman, A.K. Structural modeling, *in vitro* antiproliferative activity, and the effect of substituents on the DNA fastening and scission actions of heteroleptic copper(II) complexes with terpyridines and naproxen. *New J. Chem*., **2015**, 39, 7895–7911.

8 Ali, H.A.; Fares, H.; Darawsheh, M.; Rappocciolo, E;. Akkawi, M.; Jaber, S. Synthesis, characterization and biological activity of new mixed ligand complexes of Zn(II) naproxen with nitrogen based ligands. *Eur. J. Med. Chem*., **2015**, 89, 67–76.

9 Banti, C.N.; Giannoulis, A.D.; Kourkoumelis, N.; Owczarzak, A.M.; Kubicki, M.; Hadjikakou, S.K. Novel metallo–therapeutics of the NSAID naproxen. Interaction with intracellular components that leads the cells to apoptosis. *Dalton Trans*., **2014**, 43, 6848–6863.

10 Banti, C.N.; Gkaniatsou, E.I.; Kourkoumelis, N.; Manos, M.J.; Tasiopoulos, A.J.; Bakas, T.; Hadjikakou, S.K. Assessment of organotins against the linoleic acid, glutathione and CT–DNA. *Inorg. Chim. Acta*, **2014**, 423, 98–106.

11 Tarushi, A.; Zampakou, M.; Perontsis, S.; Lafazanis, K.; Pantazaki, A.A.; Hatzidimitriou, A.G.; Geromichalos, G.D.; Psomas, G. Manganese(II) complexes of tolfenamic acid or naproxen in polymeric structures or encapsulated in [15–MC–5] manganese(III) metallacrowns: Structure and biological activity. *Inorg. Chim. Acta*, **2018**, 483. 579–592.

12 O'Rourke, N.F. Ronaldson, M. Cameron, T.S. Wang, R. Aquino, M.A.S. Equatorial π–stacking interactions in diruthenium (II,III) tetracarboxylate complexes containing extended π–systems. *J. Mol. Struct.*, **2013**, 1052, 17–23.

13 Caglar, S.; Altay, A.; Harurluoglu, B.; Yeniceri, E.K.K.; Caglar, B.; Sahin, O. Synthesis, structural characterization and evaluation of anticancer activity of polymeric silver(I) complexes based on niflumic acid/naproxen and picoline derivatives. *J. Coord. Chem.*, **2022**, 75, 178–196.

14 Ma, C.; Zhang, B.; Zhang, S.; Zhang, R. Chiral organotin (IV) carboxylates complexes: Syntheses, characterization, and crystal structures with chiral (S)–(+)–6–methoxy–α–methyl–2–naphthaleneaceto acid ligand. *J. Organomet. Chem.*, **2011**, 696, 2165–2171.

15 Liu, Y.; Rong, D.; Xia, H.; Wang, D.; Liu, Y. Luminescence Properties and Antibacterial Activity of $[Ln_2(NNA)_6(Phen)_2]\cdot nC_3H_7NO$ (Ln = Y, Eu, Gd, Pr, Sm, Tb, Yb, n = 0, 1, 2, NAA = 1–Naphthylacetic Acid, Phen = 1,10–Phenanthroline). *Synth. React. Inorg., Metal–Org., Nano–Met. Chem.*, **2008**, 38, 635–641.

16 Xia, H.; Liu, Y.; Lu, G.; Wang, D. Crystal Structure and Interaction With Bovine Serum Albumin of Rare Earth Complex $[Nd(\alpha–C_{10}H_7CH_2COO)_3(C_{12}H_8N_2)]_2$. *Synth. React. Inorg., Metal–Org., Nano–Met. Chem.*, **2012**, 42, 145–153.

17 Liu, Y.; Xia, H.; An, X.; Zhou, L.; Wang, D. The Synthesis, Structure and Antimicrobial Study of Poly[diaqua bis(1–naphthylacetic acid)Cobalt(II) Complex $[Co(C_{12}H_9O_2)_2(H_2O)_2]_n$. *Synth. React. Inorg., Metal–Org., Nano–Met. Chem.*, 2008, 38, 356–361.

18 Liu, Y.; Rong, D.; Xia, H.; Wang, D.; Chen, L. Synthesis, crystal structure, and luminescence properties of $[TbGd(NAA)_6(phen)_2]$ and $[Tb_2(NNA)_6(phen)_2]\cdot 2C_3H_7NO$. *J. Coord. Chem.*, **2009**, 62, 1835–1845.

19 Zhao, G.; Yin, F.; Ge, H.; Li, S. Synthesis, Structure and Antibacterial Properties of *Bis*–Imidazole–*bis*(naphthalene–1–yl–acetato)copper(II), *Asian J. Chem.*, **2014**, 26, 2550–2552.

20 Dimitrakopoulou, A.; Dendrinou–Samara, C.; Pantazaki, A.A.; Alexiou, M.; Nordlander, E.; Kessissoglou, D.P. Synthesis, structure and interactions with DNA of novel tetranuclear, $[Mn_4(II/II/II/IV)]$ mixed valence complexes. *J. Inorg. Biochem.* **2008**, 102, 618–628.
